# The antigen presentation landscape of cytokine-stressed human pancreatic islets

**DOI:** 10.1016/j.celrep.2025.115927

**Published:** 2025-07-19

**Authors:** Padma P. Nanaware, J. Mauricio Calvo-Calle, Sambra D. Redick, Mason W. Tarpley, John Cruz, Cristina C. Clement, Anthony Manganaro, Erandi E. Velarde de la Cruz, Khaja Muneeruddin, Melissa Faulkner, Jennifer P. Wang, Scott A. Shaffer, David M. Harlan, Laura Santambrogio, Sally C. Kent, Lawrence J. Stern

**Affiliations:** 1 Department of Pathology, University of Massachusetts Chan Medical School, Worcester, MA, USA; 2 Program in Molecular Medicine, Diabetes Center of Excellence, University of Massachusetts Chan Medical School, Worcester, MA 01655, USA; 3 Department of Medicine, Diabetes Center of Excellence, University of Massachusetts Chan Medical School, Worcester, MA 01655, USA; 4 Departments of Physiology Biophysics and Radiation Oncology, Weill Cornell Medicine, New York, NY 10065, USA; 5 Department of Biochemistry and Molecular Biotechnology, University of Massachusetts Chan Medical School, Worcester, MA 01655, USA; 6 Senior author; 7 Lead contact

## Abstract

Type 1 diabetes (T1D) arises from T cell-mediated destruction of insulin-secreting pancreatic β cells. Inflammatory triggers have been hypothesized to induce presentation of new epitopes for pathogenic T cells, but the naturally processed MHC-bound peptides presented by primary human islet β cells are largely unknown. We used mass spectrometry to identify native and post-translationally modified self-peptides presented by MHC proteins from human cadaveric islet samples treated *in vitro* with cytokines to identify epitopes in an inflamed pancreas. Of >4,300 islet peptides presented by 60 different MHC molecules, we identified 28 autoimmune epitopes targeted by T cells from patients with T1D, 31 additional epitopes from previously identified autoantigens, and 100 additional candidate autoantigens. The epitopes derive from inflammation, unfolded protein response, and secretory hormone processing pathways. These results identify naturally processed islet peptides targeted by autoimmune T cells in T1D and provide a resource for investigating T1D etiology and progression.

## In brief

Nanaware et al. characterize peptides presented by MHC proteins in inflamed human pancreatic islets and test them for reactivity with CD4 and CD8 T cells from patients with T1D. These results identify naturally processed islet peptides targeted by autoimmune T cells and highlight pathways that generate immune epitopes in disease.

## Graphical Abstract

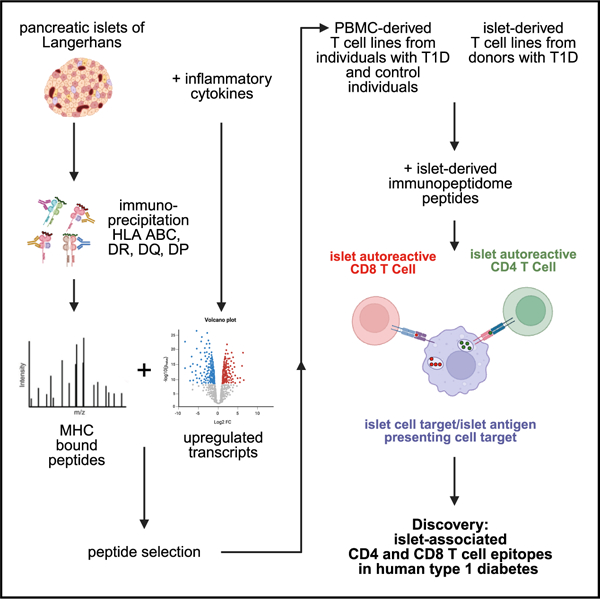

## INTRODUCTION

Type 1 diabetes (T1D) results from autoimmune destruction of insulin-secreting β cells by islet-infiltrating CD4 and CD8 T cells. Identification of the epitopes targeted by these pathogenic T cells is needed to understand disease pathogenesis, stage disease in clinical studies, and inform T1D immunotherapy efforts that have been major goals in the field. Previous efforts focused on proteins targeted by autoantibodies or by extrapolation from NOD mouse model. For most studies, including post-translational protein modification as a potential generator of autoimmune epitopes in T1D^1,2^ evidence of antigen presentation is lacking.^3^

Immunopeptidomic characterization of major histocompatibility complex (MHC)-bound peptides is an unbiased approach to identification of T cell epitopes^4,5^ proven efficient in identifying CD4 and CD8 T cell epitopes from viral pathogens^6,7^ and tumor neoantigens^8^ and has begun to be applied to autoimmune disease.^9^ While immunopeptidome studies have examined NOD mice islets,^10–12^ there are few studies with human samples. Direct mass spectroscopy of whole cytokine-stressed islets has detected T cell epitopes,^13^ and immunopeptidomes of a human pancreatic β cell line^14–16^ and whole islet protein-pulsed human monocyte-derived dendritic cells^17,18^ have yielded targets for auto-reactive T cells in T1D.

Here, we use an unbiased immunopeptidomic approach to characterize the full antigenic landscape of inflamed human islets, identifying thousands of self-peptides presented by MHC class I (MHC-I) and MHC-II molecules, including some well-characterized T1D-associated autoimmune epitopes, epitopes from previously characterized antigenic proteins, and hundreds of novel candidate autoantigens. Many of these were recognized by CD4 or CD8 T cells in peripheral blood or infiltrating the islets of T1D patients. Focusing on MHC-I and MHC-II alleles genetically associated with T1D, we identify a set of autoantigens enriched in islet cells, upregulated by exposure to inflammatory cytokines, or derived from β cell transcripts upregulated in T1D.^19^

## RESULTS

### Cytokine treatment of cultured human islets upregulates MHC-I and MHC-II antigen presentation pathways

We cultured cadaveric human islet samples with a cytokine cocktail to upregulate MHC expression and simulate *in vivo* inflammation, before transcriptomic and phenotypic analysis, isolation of MHC-bound peptides, and immunopeptidome characterization. Cadaveric islet samples were isolated from pancreata of six donors without diabetes (Table S1). For four, spleen tissue samples were available and processed in parallel. Cadaveric islet samples (4,000–70,000 islet equivalents) were cultured for 48 h with IFN-γ, IL-1β, and TNF-α.^19^ Samples before and after culture were analyzed by single-cell RNA sequencing (scRNA-seq), flow cytometry, and bulk transcriptome analysis of sorted endocrine cell populations.

**Figure 1.**
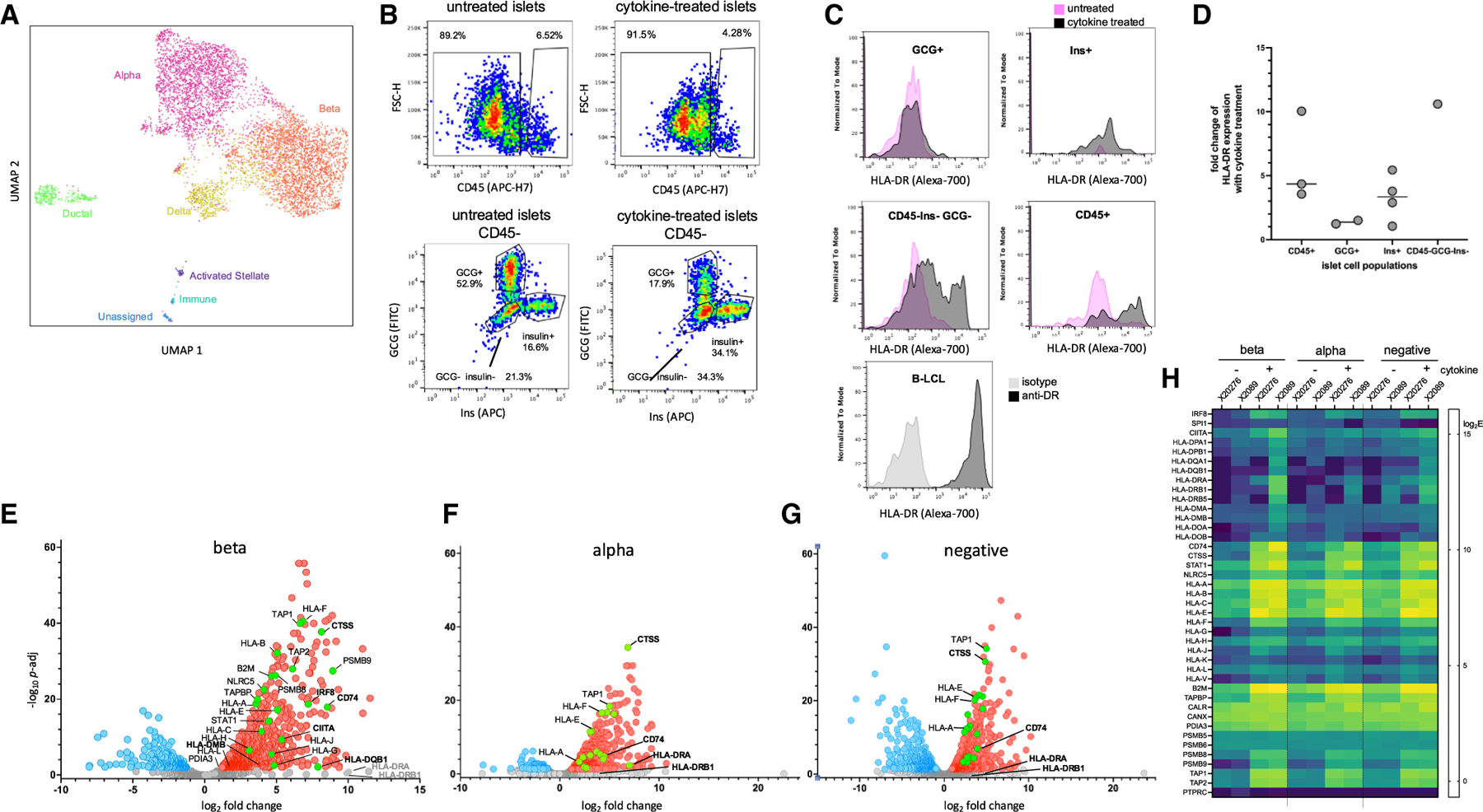
Cytokine treatment of cultured human islets upregulates MHC-I and MHC-II antigen presentation pathways (A) UMAP analysis of cultured islet RNA-seq data from two donors (HP-20289-1 and HP-20276, Table S1) showing α, β, and δ cells with smaller amounts of islet-associated ductal, acinar, and stellate cells, and low levels of immune cells. (B) Untreated and cytokine-treated islets (from donor HP-20289-1) were surface stained for CD45 and HLA-DR and intracellularly stained for insulin (Ins) and glucagon (GCG) on day 2 of culture. (C) HLA-DR surface staining of untreated and cytokine-treated samples with Ins^+^, GCG^+^+, Ins-GCG^−^, and CD45^+^ populations shown along with reference human B-lymphoblastoid cell line. (D) CD45^+^ immune cells, Ins+ β cells, and CD45-GCG-ins^−^ populations but not GCG^+^ α cells increased HLA-DR expression after cytokine treatment. (E and F) Volcano plots showing transcripts differentially expressed in bulk RNA sequencing of sorted E) β (CD45^−^, GCG^−^, Ins^+^), (F) α (CD45^−^, GCG^+^, Ins^+^−), and (G) “negative” (CD45^−^, GCG^−^, Ins^−^) populations before and after cytokine treatment from donor HP-20289–01. Red, genes upregulated >2-fold (*p*_adj_ < 0.05) by cytokine treatment, blue, genes downregulated by the same criteria. (H) Heatmap representing differential expression of MHC-I and MHC-II pathway components.

scRNA-seq analysis from two independent donors showed that islet preparations consisted of expected α, β, and δ cells with smaller amounts of islet-associated ductal, acinar, and stellate cells, and low levels of immune cells, with much variability between donors. Following cytokine treatment, most acinar cells were lost. A representative UMAP analysis of cultured islet preparations from two donors is shown in Figure 1A.

Islets samples were analyzed for hormone, CD45, and HLA-DR expression by flow cytometry after *in vitro* culture with and without the cytokine cocktail. Consistent with scRNA-seq analysis, samples were dominated by a large CD45^−^ population containing glucagon (GCG) ^+^ α cells, insulin (Ins) ^+^ β cells, and some GCG^−^/Ins^−^ cells, with a smaller frequency of CD45^+^ immune cells (Figure 1B). With cytokine treatment surviving populations shifted toward fewer CD45^+^ cells and more Ins^+^ cells. MHC-I expression increased with cytokine treatment for Ins^+^ populations as expected^20–22^ (data not shown). For Ins^+^ and CD45^+^ populations MHC-II (HLA-DR) was upregulated 1.3- to 3.2-fold and 2.1-to 7.7-fold, respectively, by the cytokine treatment (Figures 1C, 1D, and S1B). HLA-DR expression levels were lower than for professional antigen-presenting B-LCL cells. Upregulation of MHC-II also was observed for CD45^−^GCG^−^Ins cells but not GCG^+^ cells (Figures 1C and 1D).

To help understand these changes, we performed bulk RNA-seq and differential gene expression analysis of sorted β (CD45^−^, GCG^−^, Ins^+^), α (CD45^−^, GCG^+^, Ins^−^), and “negative” (CD45^−^, GCG^−^, Ins^−^) populations from two islet donors (Table S1) before and after cytokine treatment (Table S2). This analysis revealed upregulation of genes associated with MHC-I and MHC-II antigen presentation, with the largest changes on the Ins^+^ cells (Figures 1E–1G). For the β cells, cytokine treatment upregulated the MHC-I transcriptional co-activator *NLRC5*, classical (*HLA-A*, *B*, *C*, *B*_*2*_*M*) and non-classical (*HLA-E*, *F*, *G*, *H*) MHC-I genes, and MHC-I processing components (*PSMB8/9*, *TAP1/2*, *TAPBP*, *PDIA3*) (Figures 1E–1H), along with the MHC-II master transcriptional regulator *CIITA* and transcriptional activator *IRF8*, classical (*HLA-DP*,*-DQ*) and non-classical (*HLA-DM*,*-DO*) MHC-II genes, the MHC-II processing components (*CD74*, *CTSS*). *HLA-DRA* and *HLA-DRB1* were upregulated ∼1,000-fold but with high *p*_adj_ reflecting variability between the two samples (Figure 1H). Similar but smaller changes MHC-I and MHC-II antigen presentation genes were observed for GCG-secreting α and Ins^−^, GCG^−^, CD45^−^ populations (Figures 1F–1H). These results were corroborated by scRNA-seq analysis of dissociated islets with and without cytokine treatment, with upregulation of MHC-I-associated transcripts in most populations and upregulation of MHC-II-associated transcripts in β, δ, ductal, stellate, and immune cell populations (Figure S1A), again with substantial variability between samples, with one sample showing greater upregulation of the MHC-I pathway and the other showing greater upregulation of the MHC-II pathway (Figure S1A).

To relate the transcriptional changes induced *in vitro* by cytokine treatment of non-diabetic islets to those induced *in vivo* in the inflamed T1D pancreas, we compared β cell genes significantly upregulated by cytokine treatment (Figure 1E, red symbols) with those observed *ex vivo* for islet β cells from T1D versus non-diabetic pancreatic islet donors, selected by the same criteria, using previously published islet cell scRNA-seq data from two donors without diabetes and one donor with recent onset T1D^19^ combined with new data from islets of four additional donors with T1D and 17 additional donors without diabetes (Table S2). Similar sets of genes were upregulated (*p* < 0.001 by Fischer’s exact test). As an additional test we repeated this analysis using scRNA-seq data from six donors with T1D reported in the PancDB website (Human Pancreas Analysis Program [HPAP])^23–25^ (Table S2). As before, a similar set of genes was upregulated *in vivo* in islets from donors with T1D compared with cytokine treatment *in vitro* of islets from non-diabetic donors (*p* < 0.0001 by Fischer’s exact test). These results suggest that *in vitro* treatment of non-diabetic islet donors with inflammatory cytokines simulates aspects of the T1D milieu.

Overall, the *in vitro* cytokine treatment upregulated MHC-I and MHC-II surface expression along with components of the corresponding antigen presentation pathways.

### Isolation of MHC-bound peptides from cytokine-treated cadaveric pancreatic islets and spleen

**Figure 2.**
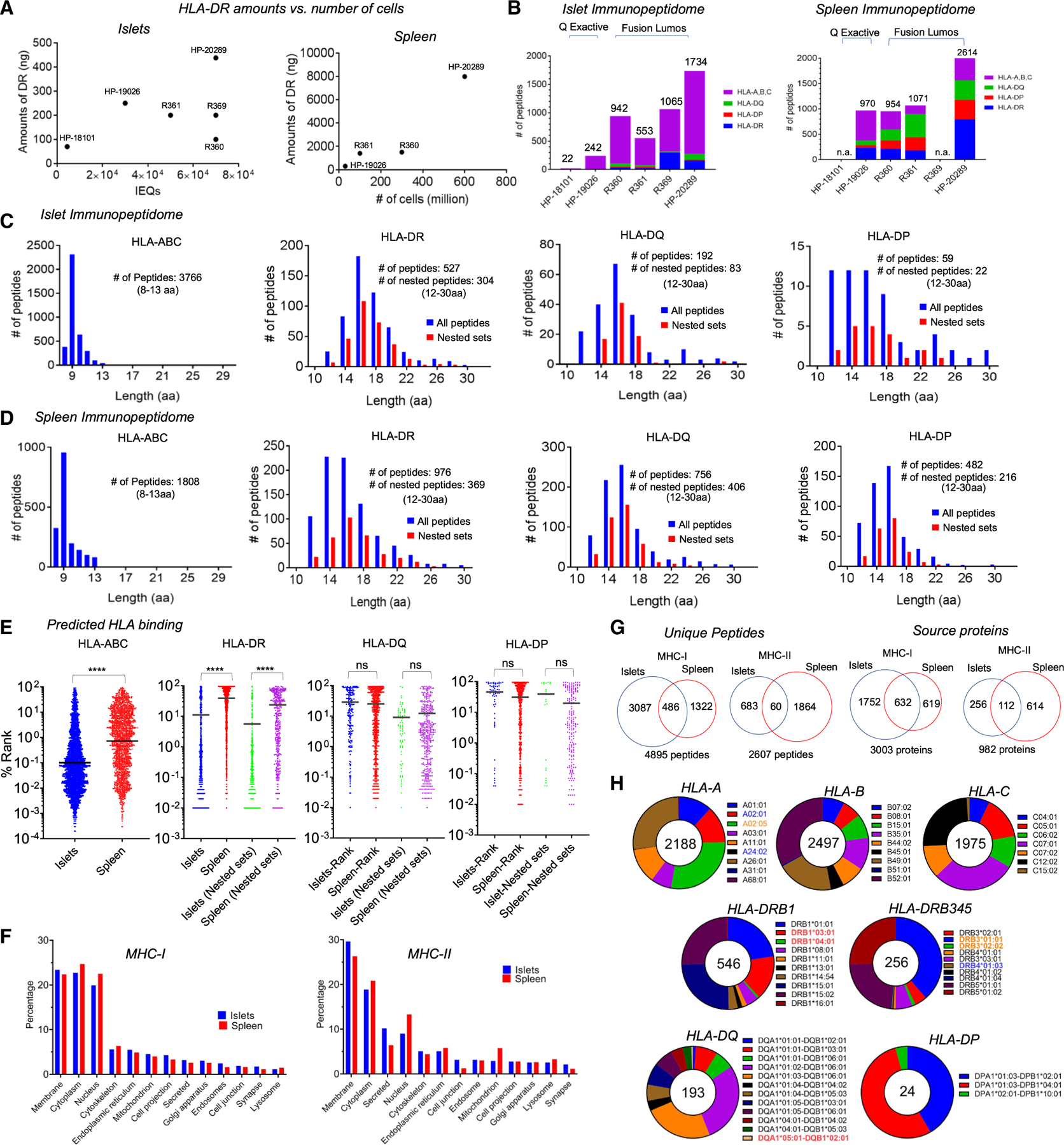
MHC-I and MHC-II immunopeptidomes from human islet and spleen samples (A) Estimated amounts of HLA-DR from each donor plotted against the number of islets procured, with a similar plot for spleen samples. (B) The number of HLA-DR, DQ, DP and HLA-A, -B, -C peptides identified from the six islet and four spleen samples used for immunopepidome analyses. Two of the six islet samples were analyzed on a Thermo Q Exactive Orbitrap Mass Spectrometer, whereas the other four were analyzed on a Thermo Orbitrap Fusion Lumos Tribid Mass Spectrometer. (C and D) Length distributions for (C) islet and (D) spleen immunopeptidomes, with peptides present in nested sets for MHC-II immunopeptidome shown in red. (E) Predicted rank percentile for HLA-ABC (NetMHCpan 4.1) and HLA-DR, HLA-DQ, HLA-DP (NetMHCIIpan 4.2) peptides compared between pancreatic islets and homologous spleen samples. Horizontal lines show geometric means (**** p < 0.0001). (F) Cellular distribution of the source proteins from GO annotation.^26,27^ (G) Overlap MHC-I and MHC-II islet and spleen immunopeptidome for unique peptides and their source proteins. (H) Assignment of the eluted islet peptides to HLA allotypes from tissue donors. HLA alleles most strongly associated with increased risk for T1D in red, alleles associated with increased risk for T1D after accounting for linkage disequilibrium with the major DR-DQ risk haplotypes in blue, HLA molecules in the same allele family as risk alleles and cases where only low-resolution HLA typing was available in orange, and other alleles of interest in T1D research in magenta. See Table S1 legend for details.

We analyzed the peptides carried by these MHC proteins using an immunopeptidomics workflow (Figure S2). We monitored the amount of HLA-DR present in lysates before and after immunoaffinity to assess the quality of the preparations. HLA-DR yields roughly scaled with the number of islet equivalents, although islet samples R369 and particularly R360 had lower than expected HLA-DR recovery (Figure 2A, left). Spleen samples yielded 5- to 20-fold more HLA-DR than the corresponding islet samples (Figure 2A, right), consistent with the greater number of cells analyzed and the expected increased HLA-DR expression.

In all of the eluted peptide samples, including those eluted from protein A-only and isotype-control antibody-conjugated beads, we observed many overlapping fragments of pancreatic secretory hormones. Fragments of insulin and glucagon were particularly prominent, but fragments of chromogranin A (CHGA), pancreatic prohormone (PPY), and secretogranins (SCG-1, -3, and -5) also were observed (Figure S3; Table S4), suggesting that these peptides could represent co-purifying secretory hormone processing intermediates and not MHC-bound peptides, even for samples eluted from MHC immunoaffinity columns. This background complicated confident identification of MHC-bound peptides, However, some peptides were found only in the MHC elutions and not control samples, away from the problematic regions with high densities of secretory hormone fragments, and appeared to represent bona fide naturally processed MHC-bound peptides (Figure S3), including the “WE14” CHGA epitope targeted by circulating T cells in patients with T1D^28^ and diabetogenic CD4 T cells in NOD mice^29^ and two insulin-derived peptides previously identified as T1D-associated autoimmune epitopes associated with β cell destruction^16,29,30^ (Table 1). In addition, 11 new peptides from previously identified T cell antigens insulin, CHGA, and SCG5,^3^ and 17 peptides from new autoantigens glucagon, pancreatic prohormone, SCG1 (also known as chromogranin-B), and SCG3, were included in subsequent analyses (Tables 1 and S4; Figure S3).

### Characterization of MHC-I and MHC-II peptidomes

After removal of background peptides, the total number of the MHC-associated peptides identified ranged from 22 to 1,734 for islet samples and 970 to 2,614 for spleen samples (Figures 2B; Tables S3 and S4). The spleen samples had a larger proportion of MHC-II-derived peptides (HLA-DR, -DQ, -DP), as expected from the larger fraction of conventional antigen-presenting cells. Overall, we identified 3,766 unique MHC-I peptides (HLA-ABC) in islet samples (Figure 2C), and 1,808 in spleen samples (Figure 2D), in both cases with median lengths of 9 amino acids. For MHC-II, we identified 778 unique MHC-II peptides in islet samples: 527 from HLA-DR, 192 from HLA-DQ, and 59 from HLA-DP, consistent with the relative abundance of these proteins. Yields from spleen samples followed the same pattern. Many MHC-II peptides were present as nested sets characteristic of MHC-II processing where peptide termini extending from the MHC-II binding site are variably trimmed by endosomal proteases. These represented ∼40% of the total MHC-II peptides, with no significant differences in proportion between islet or spleen samples or between HLA-DR, HLA-DQ, or HLA-DP. The median length of MHC-II peptides was 16, again with no significant differences between islets or spleen, or between the different MHC-II proteins. However, the islet HLA-DP peptide length distribution was skewed somewhat to shorter peptides indicative of possible background contamination in the non-nested set peptides.^31^ Predicted binding analysis for the MHC-I and HLA-DR peptides indicated stronger binding in islets compared with the splenic cells (Figure 2E). Although no significant differences were observed for HLA-DQ and HLA-DP. Finally, the distribution of source proteins between cytoplasm, membrane, extracellular and intracellular organelles was similar between islets and spleen for both MHC-I and MHC-II peptidomes (Figure 2F). Overall, the similarity of peptide-length distributions, MHC-II nested set distribution, and source protein localization for the islet and spleen peptidomes indicate that MHC-I and MHC-II antigen-processing pathways function similarly in the two tissue types.

Despite these broad similarities the particular peptides present in the two tissue types were considerably different, with <10% overlap of individual peptides, and only 10%–20% overlap of source proteins (Figure 2G). These differences likely reflect tissue-specific expression patterns together with effects of *in vitro* culture and cytokine treatment for the islet samples.

To facilitate subsequent analyses, we used a machine-learning-based MHC-peptide binding prediction algorithm^32^ (Figures 2H; Tables S5 and S6) to assign eluted peptides to HLA allotypes expressed by the tissue sample donors (Table S1). These include HLA alleles most closely associated with increased risk for T1D: DQA1*05:01-DQB1*02:01 (DQ2.5), DRB1*03:01 (DR3), and DRB1*04:01 (DR4),^33^ shown in red in Figure 2H; and HLA alleles associated with increased risk for T1D after accounting for linkage disequilibrium with the major risk haplotype: A*02:01 (A2), A*24:01 (A24), and DRB4*01:03 (DR53),^34^ shown in blue. HLA alleles in the same allele family as risk alleles (A*02:05) or very closely linked in the same HLA-DRB locus (DRB3*01:01, DRB3*02:01), with high sequence homology and similar MHC-peptide binding profiles, are shown in orange. For HLA-A2 (A*02:01) and HLA-DR3 (DRB1*03:01), these assignments were confirmed by direct MHC-peptide binding assays (Figure S4).

### Identification of post-translationally modified peptides

**Figure 3.**
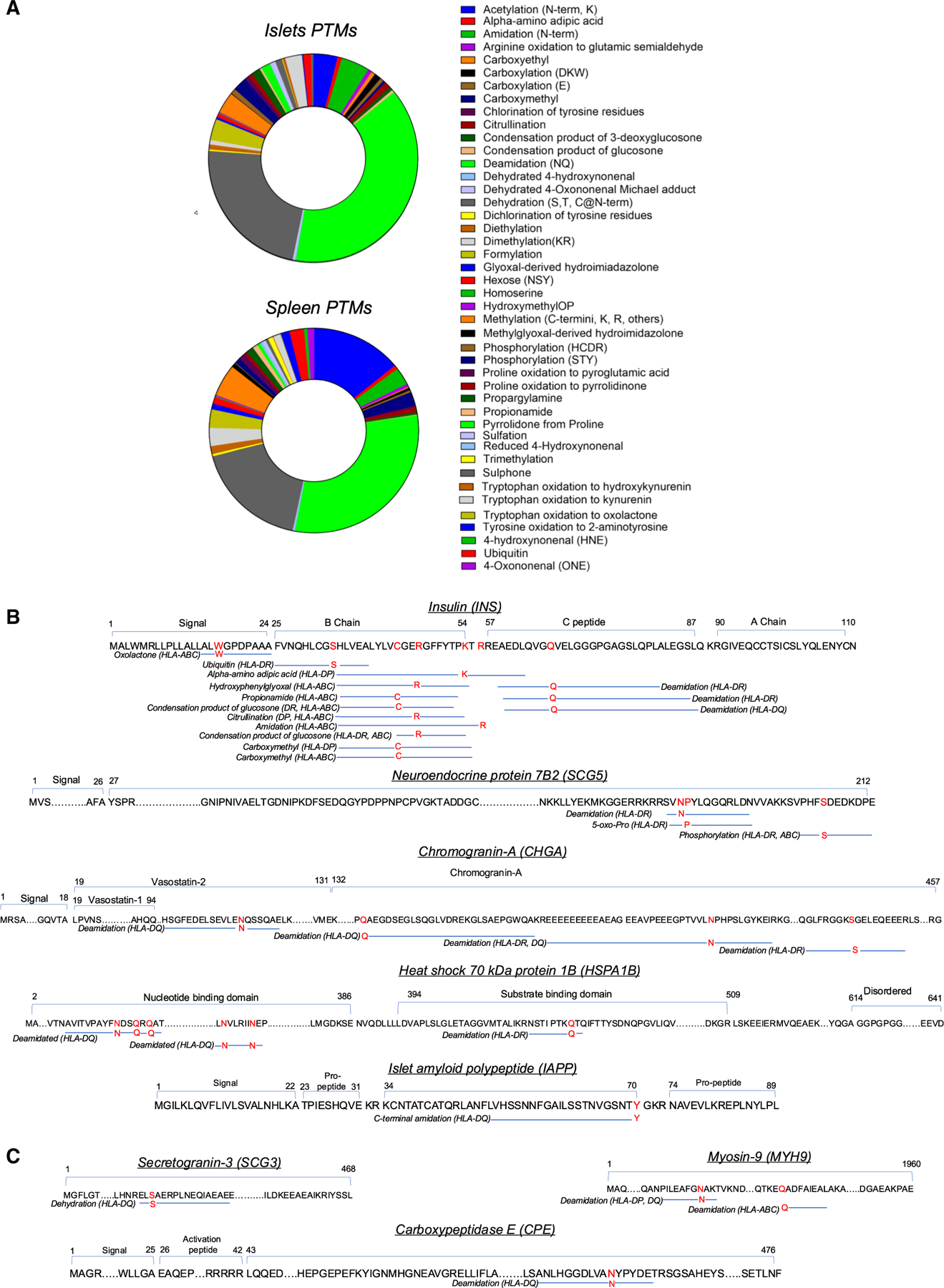
Identification of post-translationally modified peptides (A) MHC-I and MHC-II eluted immunopeptidomes from cytokine-treated human islets and matched spleen samples were analyzed for oxidative-stress-related PTMs. PTMs with A scores >13 (corresponding to statistical significance of *p* < 0.05 for probability of site localization) were summed for each modification based on the fraction of total intensity from peptides with modified as compared with non-modified residues. (B) PTMs identified on peptides derived from known autoantigens associated with T1D. (C) PTMs identified on peptides derived from new potential autoantigens.

Post-translationally modified (PTM) peptides add antigenic diversity and are emerging as potential autoantigens contributing to T1D autoimmunity.^1,35–37^ We searched for oxidative modifications, advanced glycation end-products, citrullination, acetylation, carbamylation, deamidation, phosphorylation, ubiquitination, and other PTMs in immunopeptidomes from cytokine-stressed human islets and matched spleen samples (Table S7). Many PTMs were observed in both samples, although with different distributions (Figure 3A). We identified deamidation of the two asparagines on HSPA1B-derived peptide LNVLRIINE presented by HLA-DQ both in islets and spleen samples. The other modifications reported in Figure 3B (previously characterized T1D-associated antigens) and Figure 3C (new candidate epitopes) are unique to islets. For insulin, the richest source of autoimmune epitopes in humans^3^ and mice,^11^ we observed deamidation, citrullination, amidation, ubiquitination, carboxymethylation, propionamide, α-amino adipic acid, oxolactone, and advanced glycation end-products such as glucosone on peptides presented by both MHC-I and MHC-II molecules (Figure 3B). We observed deamidation of insulin Q66 on three MHC-II-presented peptides (Figure 3B), this modification has been shown to increase binding to HLA-DQA1*05:01-DQB1*03:02.^38^ Asn/Gln deamidation was the most common PTM, with three such modifications in chromogranin A (CHGA) peptides. We observed additional PTMs including arginine amidation, cysteine carboxymethylation, serine dehydration and phosphorylation, and pyroglutamate formation on other previously characterized and new candidate antigens (Figures 3B and 3C).

### Spliced and hybrid peptides

Spliced peptides representing fusions between two fragments of the same polypeptide, and hybrid peptides formed by fusions between fragments of different peptide, have emerged as unconventional T cell epitopes, with hybrid peptides of particular interest in T1D.^39^ Following an approach used to identify hybrid insulin peptide epitopes recognized by diabetogenic CD4^+^ T cells in NOD mice,^39,40^ we searched the MHC-I and MHC-II islet peptidomes for hybrid peptides formed from fusions of fragments of abundant secretory granule components insulin, CHGA, islet amyloid polypeptide (IAPP), and SCG-1 (see STAR Methods). We did not confidently identify any fusion peptide sequences corresponding to MS fragmentation spectra in any of the samples that we analyzed. It is possible that such peptides are present in samples but below the limit of detection, or that they could be identified with relaxed search criteria or alternate search strategies.

### Previously identified MHC-I and MHC-II T cell epitopes and additional epitopes from previously reported T1D-associated antigens

To assess the potential utility of these results for identifying new autoimmune epitopes, we searched the islet peptidomes for known T1D-associated T cell epitopes. We identified six such cases (Table 1). The well-known insulin-derived CD8 T cell epitope HLVEALYLV presented by HLA-A*02:01^16,41^ and reported to be associated with β cell destruction^42^ was identified in the islet MHC-I peptidome of donors HP-20289 and HP-18101, where it was predicted to be presented by the HLA-A2 family allele A*02:05 and the T1D-associated risk allele A*24:02, respectively. Another insulin peptide derived from the signal sequence, ALWGPDPAAA, reported to elicit CD8^+^ T cell response in HLA-A*02:01^+^ T1D donors,^42^ was identified in the islet MHC-I peptidome of donors R361 and R369, where it was presented by HLA-A*02:01. Two overlapping peptides derived from CHGA, YGFRGPGPQL and FRFPFPQL, presented by HLA-B*49:01 and HLA-*02:05, respectively, were identical to those reported in an earlier study of peptides eluted from cytokine-treated human islets, where they were assigned to HLA-C alleles.^16^ Another autoimmune epitope, FTNKFLVEL from neuroendocrine convertase 2 (*PCSK2*), which processes proinsulin in islet β cell granules by cleaving between the C peptide and B chain, previously was identified in immunopeptidome studies of an islet β cell line and shown to be recognized by peripheral CD8 T cells.^16^ We identified this peptide in the islet peptidome from donor HP-20289 presented by A*02:05 (Table 1). Finally, eight nested peptides surrounding the predicted DQ6 (DQA1*03:01/DQB1*06:01) core epitope NQPGVLIQV corresponds to a partial overlap with the known CD4^+^ T1D-associated MHC-II epitope TKQTQIFTTYSDNQPGVLI, from HSPA1B (Table 1). Proliferative T cell responses previously have been mapped to this epitope in peripheral blood of children recently diagnosed with T1D, although no evidence of naturally processing and presentation were reported and presenting HLA molecules are unknown.^43^

In addition to these exact matches, we identified many cases in which eluted peptides derived from protein antigens known to contain other T1D-associated T cell epitopes (Table 1). Within the islet MHC-I peptidome, 18 additional epitopes from known autoantigens were identified. Two insulin-derived peptides were identified, one (residues 32–40) largely overlapping the classic HLA-A2-restricted MHC-I epitope, and a second (residues 22–30) partially overlapping the signal sequence. One peptide derived from CHGA, two from KIF1A, a kinesin-like protein, and three from secretogranin 5 (SCG5), the neuroendocrine protein 7B2, which is found in insulin granules where it activates proprotein convertase. Like insulin, CHGA, KIF1A, and SCG5 are known sources of CD8 T cell epitopes associated with T1D.^3^ Five peptides derived from HSPA1B, one from heat shock protein family A member 5 (HSPA5), the ER-chaperone also known as GRP78 or BiP, another from the 60 kDa heat shock protein Hsp60 (HSPD1), and four peptides from PTPRN, the receptor-type tyrosine-protein-phosphatase N, also known as IA-2, a target of autoantibodies used to stage presymptomatic T1D^44^; these proteins all contain previously characterized T1D-associated CD4 T cell epitopes.^3^ Within the MHC-II peptidomes, we identified 15 individual peptides (10 unique core epitopes) from 7 proteins previously identified as sources of T1D T cell epitopes: CHGA, HSPA1B, HSPA5, HSPD1, and SCG5, which each also provided MHC-I peptides as just described; IAPP and PCSK2, which cleaves insulin between the A chain and C peptide. CHGA, HSPA1B, HSPA5, HSPD1, and IAPP are sources of known CD4 T cell epitopes associated with T1D, and PCSK2 and SCG5 are sources of known CD8 T cell epitopes.^3^ The previously identified MHC-I or MHC-II epitopes within these proteins differ from the peptides we observed likely because of the binding preferences of the particular HLA alleles in our donors, which in several cases have been genetically associated with an increased risk of T1D.

### Candidate epitopes derived from source proteins enriched in pancreas, upregulated by inflammatory cytokines, or upregulated in β cells from donors with T1D

**Figure 4.**
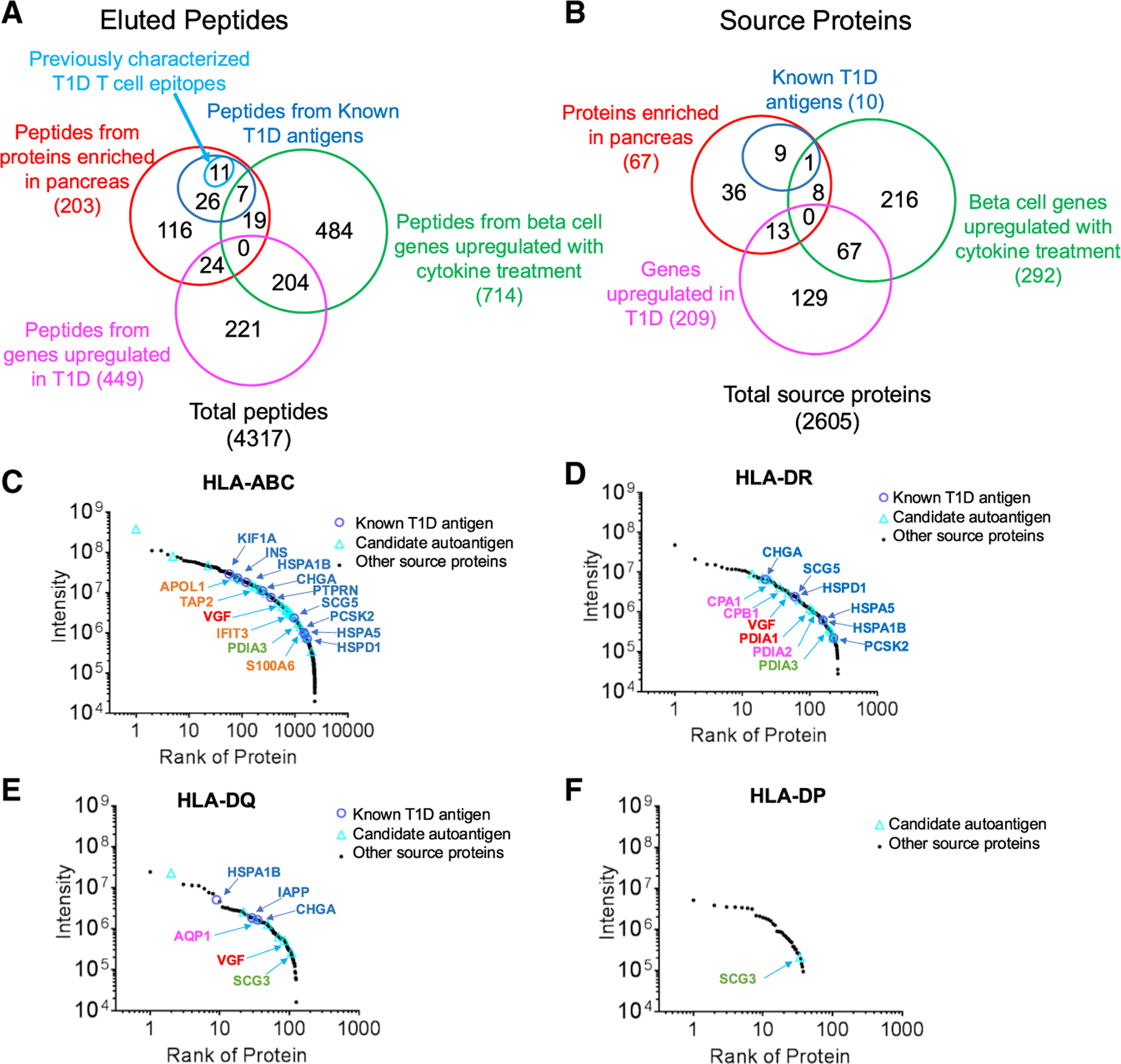
Candidate T1D-associated epitopes and their source proteins (A) Venn diagram showing overlap of eluted peptides deriving from proteins enriched in pancreas (red), genes upregulated >2-fold (*p*_adj_ < 0.05) in islet β cells from donors with T1D compared with non-diabetic controls (magenta), and genes upregulated >2-fold (*p*_adj_ < 0.05) in β cells from control donors after *in vitro* treatment with inflammatory cytokines (green). Previously characterized T1D cell epitopes (light blue) and new peptides identified from known T1D antigens (dark blue) are indicated. (B) Corresponding Venn diagram for source proteins of the eluted epitopes. (C–F) Rank distribution plots for the source proteins from HLA-ABC, HLA-DR, HLA-DQ, and HLA-DP immunopeptidomes. The precursor intensities for the peptides identified from each source proteins in ABC, or DR or DQ or DP elution were summed, ranked based on the total intensity and plotted. Known T1D antigens and the representative candidate auto-antigens are indicated for each graph.

As the set of peptides eluted from cytokine-stressed islet samples contained previously reported T1D-associated epitopes and many new epitopes from previously reported T1D-associated antigens, we considered that it might also contain novel T1D-related T cell epitopes and selected for further consideration peptides derived from proteins enriched in the pancreas, genes upregulated by *in vitro* cytokine treatment, and genes upregulated in T1D compared with control organ donors. We used the human protein atlas to identify proteins previously reported by western blot or mass spectrometry to be enriched in pancreas relative to other tissues,^45–48^ and identified 203 peptides (Figure 4A, red; Table S8). Using bulk RNA-seq analysis of cytokine-treated versus untreated islets (Table S3), we identified 714 peptides derived from genes upregulated in islet β cells with cytokine treatment (Figure 4A, magenta). Finally, we used the database of genes upregulated in islet β cells from T1D compared with control individuals (Table S3) and identified 449 peptides (Figure 4A, green). Overall there were 1068 new candidate epitopes identified by this procedure, along with 11 previously identified epitopes and 33 new epitopes from previously identified source proteins (Table S8). Of the total 2,605 immunopeptidome source proteins, 18% (479 proteins) were identified as pancreas enriched, T1D upregulated, or cytokine upregulated (Figures 4B; Table S8). No source protein met all three criteria.

To visualize the relative abundances of the known and candidate epitopes, we displayed them on rank abundance plots for the entire MHC-I and MHC-II immunopeptidomes, summing intensities of the peptides from each source protein (Figures 4C–4F), Peptides from known T1D antigens and new candidate autoantigens are present in similar ranges across the abundance spectrum.

### T cell recognition of selected peptides in PBMCs from individuals with T1D

We assessed whether these candidate epitopes were recognized by T1D and control donors focusing on the T1D risk haplotype *HLA-DR3/HLA-DQ2*.*5/HLA-A2*. Of the 1,117 new candidate epitopes in Table S8, 31 candidate epitopes were assigned to HLA-DRB1*03:01 (DR3), 66 to HLA-A*02:01 (A2), and a single one to HLA-DQA1*05:01/DQB1*02:01 (DQ2.5). Corresponding peptides were synthesized and distributed into pools of 5–10 peptides (Table S9) for screening using PBMCs from donors with T1D and controls. The DR3 group includes a new epitope from known autoantigen Hsp70 (gene *HSPA1B*) described above as peptide 15; all the other peptides represent new candidate autoantigens.

**Figure 5.**
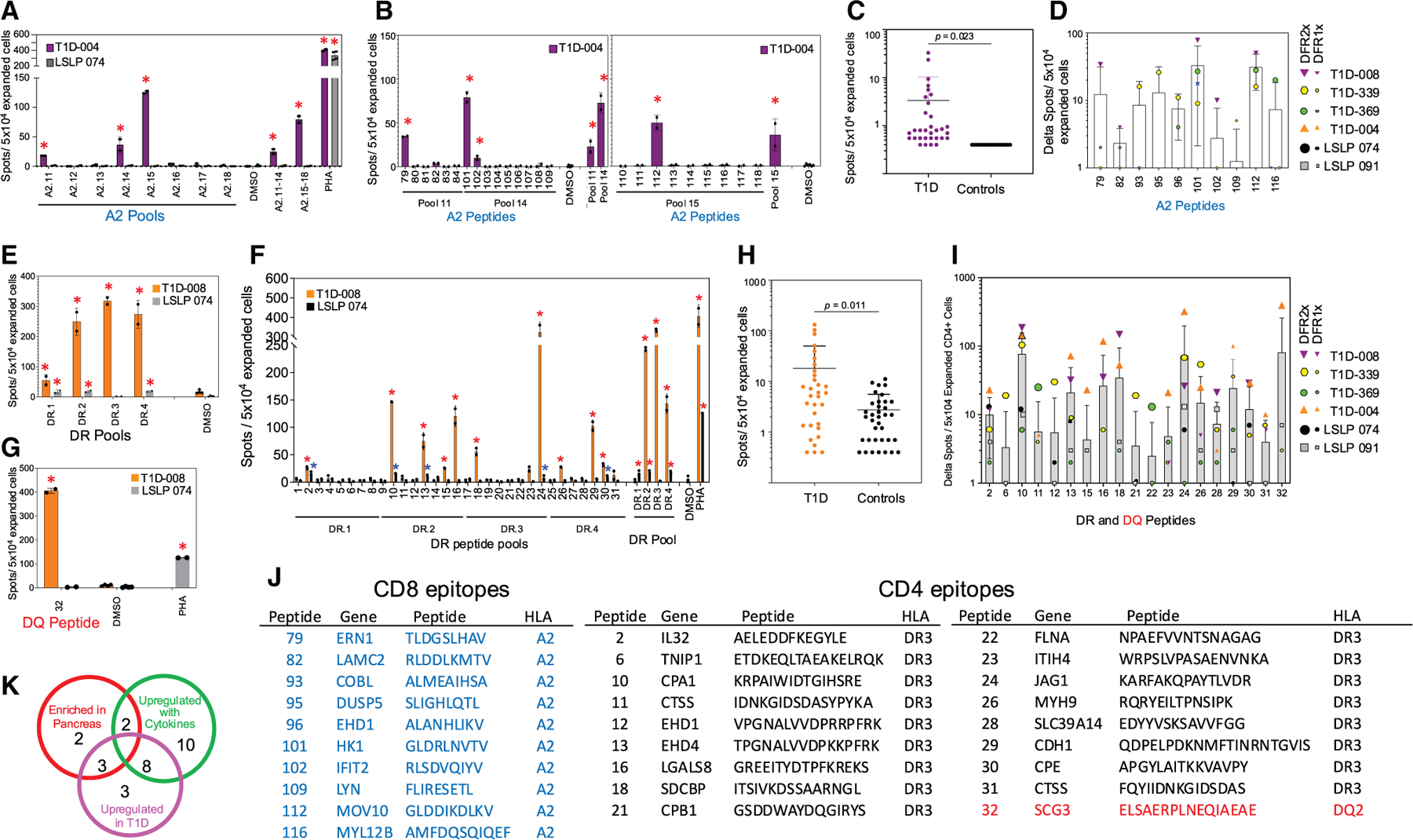
Validation of T cell responses to candidate epitopes in donors with T1D and controls (A) Response of PBMC-derived expanded CD8^+^ T cells for donor T1D-004 and control donor LSLP 074 to pools of candidate epitopes presented by HLA-A2 as measured by IFN-γ ELISpot. (B) Deconvolution of the response to positive pools. (C) Total response to individual peptides by expanded CD8^+^ T cells from five donors with T1D and two controls. (D) Summary of positive CD8 T responses observed in a total of four donors with T1D and two controls. No responses were observed for donors LSLP 074 and LSLP 095. (E) Response of PBMC-derived CD4^+^ T cells from donor T1D-008 and control donor LSLP 074, expanded *in vitro*, and tested for IFN-γ secretion in response to pools of candidate epitopes presented by HLA-DR3. (F) Deconvolution of the response to positive pools. (G) Response of same donors as € to a candidate epitope presented by HLA-DQ2.5. (H) Total response to individual peptides by expanded CD4^+^ T cells from five donors with T1D and two controls. (I) Summary of positive CD4 T responses observed in a total of five donors with T1D and two controls. (J) Sequence and source genes for candidate epitopes validated by PBMC responses in T1D donors. (K) Summary of validated epitopes grouped according to source protein characteristics. (A–H and I) Bars represent mean responses, and error bars the standard deviation. Peptides, PHA tested in duplicate; DMSO controls 4–10 replicates. Red asterisks and large symbols in (D and I) show statistically significant ELISpot responses at DFR2x level, blue asterisks and small symbols DFR1x. Significance of differences between T1D and control donors assessed by Student’s t test with Welch’s correction. For plots and statistical tests, wells for which no T cell response was observed were assigned an imputed minimum value equal to the assay detection limit of 0.3 spw. T cell lines were generated by a single *in vitro* expansion of CD4 and CD8 T cells with peptide pools.

Analysis of CD8 T cell response to the candidate epitopes is shown in Figures 5A–5D. CD8^+^ T cells were isolated from PBMC by magnetic bead positive selection and responding cells were expanded by culture with peptide pools and tested for reactivity by IFN-γ ELISpot. Pools exhibiting positive responses were rescreened to assess responses to individual peptides. Viral epitopes and self-peptide pools were used as positive and negative controls.^6,49^ Representative results for one donor with T1D (T1D-008D) and one control donor (LSLP-074) are shown in Figures 5A and 5B. For T1D-004, CD8^+^ cells expanded with pools A2.11–14 or pools A2.15–18 recognized A2 peptide pools 11, 14, and 15 (Figure 5A). The expanded cells were re-tested with individual peptides comprising the positive pools, revealing responses to peptides 79 in pool 11, 101 and 102 in pool 14, and 112 in pool 15 (Figure 5B). Expanded cells from control donor LSLP-074 did not respond to any of the A2 peptide pools, although strong positive control responses to PHA (Figure 5A) and the positive control CEF pool were observed (Figure S5A). Summary results from a total of five T1D donors and two control donors are shown in Figures 5C and 5D. Expanded CD8 T cells from donors with T1D exhibited a mean of 98 ± 64 spots per well (spw) over background for the A2 candidate epitopes, whereas neither control donor exhibited a positive response (Table S9). Individual peptides were recognized with an average of 4 spw for the T1D donors (Figure 5C). Eight new epitopes were recognized by strong CD8 T cell responses (statistically significant at 2-fold over the background, DFR2x^50^), with two additional epitopes recognized by weaker responses (significant over background, DFR1X^50^) (Figure 5D).

CD4^+^ T cell responses were assessed similarly. We observed a broader response in both the number of peptides recognized and number of donors responding to CD4^+^ cells expanded with the DR3/DQ2.5 peptide pools (Figures 5E–5H). Representative donor T1D-004 responded to all four peptide pools tested (Figure 5E) with responses to individual DR3 peptides 2, 10, 13, 16, 18, 24, and 30 (Figure 5F), and individual DQ2.5 peptide 32 (Figure 5G). Control donor LSLP-074 responded to three DR pools (Figure 5E), a control HHV-6B pool (Figure S5B), and individual peptides 2, 10, 13, and 24 (Figure 5F), although with frequencies of responding T cells ∼10-fold lower than for the T1D-004 (Figure 5E). Expanded CD4 T cells from donors with T1D exhibited a mean of 489 ± 370 spw over background for the DR3 candidate epitopes compared with 71 ± 1 for the control donors (Table S9). Individual peptides were recognized with an average of 19.4 spw for T1D donors compared 2.5 spw controls (Figure 5H). Overall, 17 individual DR3 peptides and the single DQ2.5 peptide were recognized (Figure 5I).

In total, 28 new candidate epitopes were validated by T cell responses in T1D donors, including 10 peptides presented by A2, 17 by DR3, and 1 by DQ2 (Figure 5J). A2 peptide 101 from hexokinase 1 (gene name *HK1*) and DR3 peptide 10 from carboxypeptidase A1 (*CPA1*) were recognized by all four donors tested. Five other peptides were recognized by three of the donors: A2 peptide 112 from *MOV10* and DR3 peptides 2, 16, 24, and 30 from *IL32*, *LGALS8*, *JAG1*, and *CPE*, respectively. Proteins enriched in the pancreas and from β cell genes upregulated with cytokines or in donors with T1D compared with controls all contributed validated epitopes (Figure 5K).

Using a similar approach we tested additional sets of candidate epitopes. First, we extended the analysis of Figure 4A using the HPAP database of genes enriched in T1D, identifying 295 genes for which eluted peptides were found in the islet immunopeptidomes (Tables S2 and S8), of which 19 were assigned to HLA-A2 or HLA-DR3 (pools HPAP-A2 and HPAP-DR3, Figure S5). These were tested in pool deconvolution IFN-γ ELISpot assays using PBMCs from four T1D donors (including two used in the analysis described above) and four controls. Peptide 151 from *CPE* exhibited a positive response in one of the control donors (Figure S5C; Table S9). Second, we evaluated T cell responses to eluted peptides from five islet secretory hormones for which mass spectrometry intensities were substantially above the background signal from processing intermediates (Figure S3; Table S4), using the same donors and controls. IFN-γ responses from CD4 T cells were observed for four peptides in control donor LSLP-153: 180 from *CHGA*, 184 from *SCG1*, and 186, 187, 188, and 189 from *3SCG3* (Figure S5C). Finally, we tested nine eluted peptides carrying post-translational modifications from Figure 3B for which synthetic versions were readily available, along with their corresponding non-modified native peptide sequences (Table S9). Eluted peptide 146–1 derived from insulin and carried a carboxymethyl cysteine modification (GSHLVEALYLVC*GERGF) that introduces a negative charge. This peptide as well as the native non-modified peptide 146 were recognized by IFN-γ-secreting CD4 T cells in expanded PBMCs from a T1D donor (Figure S6), with both peptides showing tight binding to HLA-DR3 with IC_50_ of 270–300 nM (Figure S4A).

### T cell recognition of selected peptides in T cell lines expanded from pancreatic islets of donors with T1D

**Figure 6.**
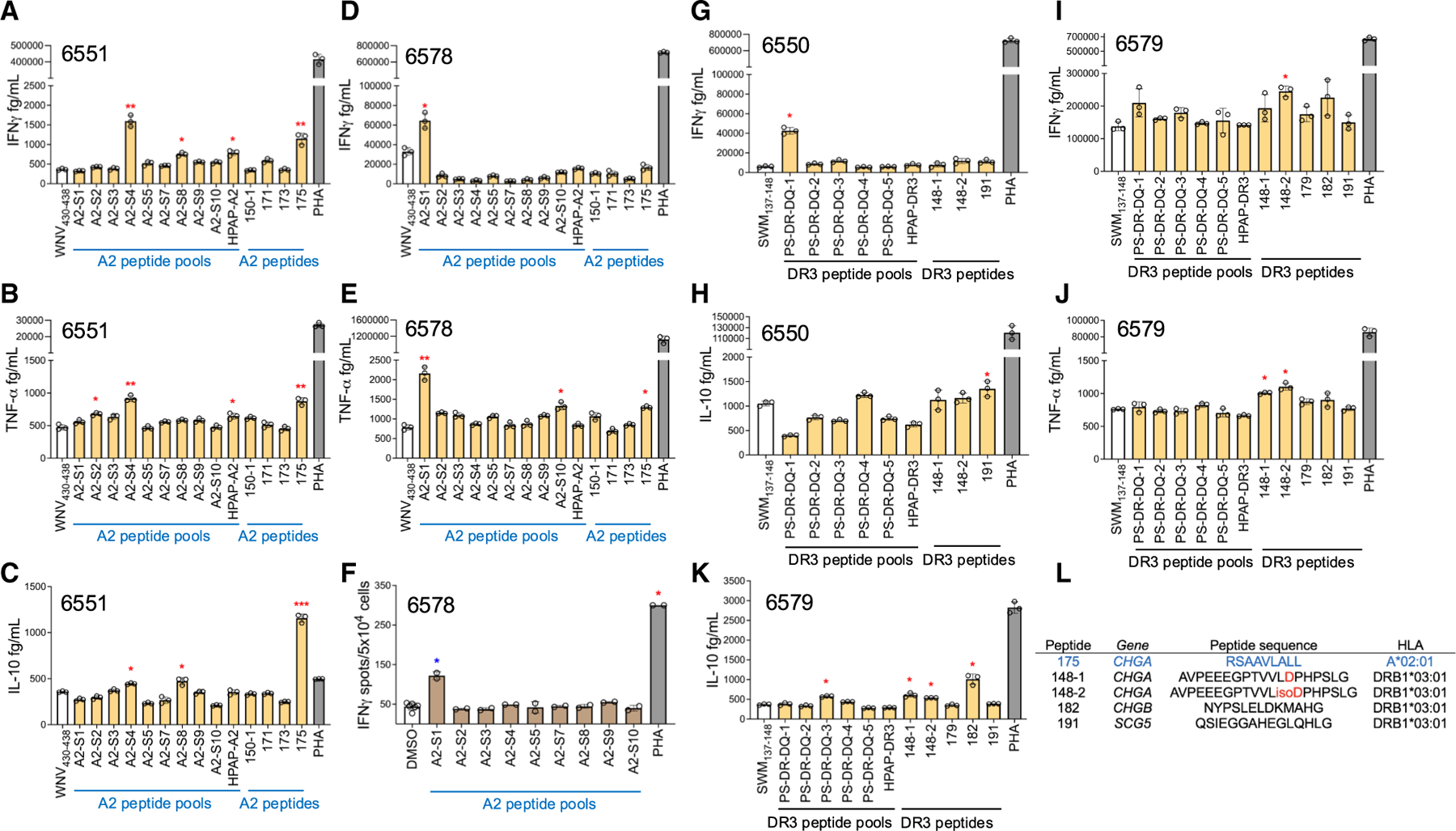
T cell lines derived from islets of donors with T1D recognize peptides from the immunopeptidome of inflammatory cytokine-treated islets A2-derived peptide pools and peptides (Table S9) recognized by islet-derived T cells lines from donor 6551 with IFN-γ, TNF-α, and IL-10 secretion are shown (A–C). A2-derived peptide pool recognized by islet-derived T cells lines from donor 6578 with IFN-γ and TNF-α secretion are shown (D and E) and in IFN-γ ELISpot; statistically significant responses are indicated by red and blue asterisks for DFR2x and DFR1x, as in Figure 5 (F). DR3-derived peptide pool and peptide recognized by islet-derived T cell lines from donor 6550 with IFN-γ and IL-10 secretion are shown (G and H). DR3 peptides and peptide pool recognized by islet-derived T cells lines from donor 6579 with IFN-γ, TNF-α, and IL-10 secretion are shown (I–K). Cytokines not detected are not shown. Sequence and source genes for candidate epitopes, modifications in red, validated by islet-derived T cell responses from donors with T1D are shown (L). Positive responses to PHA are indicated in each panel. Control peptides for A*02:01 reactivity is West Nile virus (WNV)_430–439_ and for DRB1*03:01 reactivity is sperm whale myoglobin (SWM)_137–148_, respectively, and were used as comparators in paired parametric Student’s t tests (GraphPad Prism version 10.2.1) and statistically significant responses are indicated (*p < 0.05, **p < 0.01, ***p < 0.001). Bars show mean responses and error bars show standard deviation.

Finally, we investigated the response to eluted peptides in T cell lines expanded from isolated islets of four donors with T1D^51^ (Table S9). Islet donors are nPOD slice donors 6551 (20.7-year-old male with 0.58-year duration of T1D), 6578 (11.95-year-old female with demise at T1D onset), 6550 (25.06-year-old male with demise at T1D onset), and 6579 (13.91-year-old female with 1-year duration of T1D) (Table S1). For each assay, 7–10 individual islet-derived T cell lines from each donor were combined and activation was assessed by secretion of IFN-γ, TNF-α, or IL-10 measured by a cytokine bead array assay. Islet-derived T cell lines from A*02:01^+^ donor 6551 recognized peptide pools A2-S2, S4, S8, and HPAP-A2 by cytokine secretion. Peptide 175 (from *CHGA*) was recognized by strong IFN-γ, TNF-α, and IL-10 secretion (Figures 6A–6C). Islet-derived T cell lines from A*02:01^+^ donor 6578 recognized peptide pool A2-S1 by secretion of IFN-γ and TNF-α (Figures 6D and 6E) and IFN-γ ELISpot (Figure 6F). Peptide pool A2-S1 contained six peptides (79, 80, 83, 89, 129, and 131) derived from source proteins enriched in pancreas that showed HLA-A2 binding with IC_50_ ∼130 nM to 1 μM (Figure S4). Responses to peptide 79 also were observed in patient T1D-008 (Figure 5C). Islet-derived T cell lines from DRB1*03:01^+^ donor 6550 recognized peptide pool PS-DR-DQ-1 with IFN-γ secretion and peptide 191 (from *SCG5*) with IL-10 secretion (Figures 6G and 6H). Islet-derived T cell lines from DRB1*03:01^+^ donor 6579 recognized peptide 148 (from *CHGA*) carrying post-translational modification of asparagine to aspartate (148–1) or isoaspartate (148–2) with secretion of IFN-γ, TNF-α, and IL-10, and peptide 182 (from *CHGB*) with secretion of IL-10 (Figures 6I–6K).

Peptide pools A2-S5 and HPAP-A2, recognized by islets from donor 6551, contained no previously characterized T1D-associated T cell epitope and so must each contain at least one new T1D-associated epitope. T cell responses to *CHGB* have not been previously reported so peptide 182 recognized by islets from donor 6579 is a new T1D-associated epitope. Peptides 175, 148–1, 148–2, recognized by islets from donor 6579, are new T1D-associated epitopes from the previously characterized antigen *CHGA*, and peptide 191, recognized by donor 6550, is a new T1D-associated epitope from the previously characterized T1D-associated antigen *SCG5* (Figure 6L; Table S9). Overall, these studies with four islet donors with <1 year of T1D duration, including two with demise at T1D onset, highlight a potential role in disease onset for CD4 and CD8 T cells recognizing peptides derived from secretory hormones *CHGA*, *CHGB*, and *SCG5*.

### Additional candidate epitopes

To help inform continuing T1D epitope identification studies, we prepared a list of candidate epitopes presented by HLA alleles other than HLA-A2, HLA-DR3, and HLA-DQ2.5. As before, we selected eluted peptides identified in cytokine-stressed islet preparations derived from proteins enriched in the pancreas or genes induced in β cells by cytokine stress or upregulated in T1D compared with control patients. We further selected for characteristics of the confirmed T cell epitopes reported in Figure 5, including source proteins involved in pathways previously implicated in T1D or related autoimmune diseases. In many cases these derived from the same source proteins as the validated epitopes. Overall, we identified 324 new candidate epitopes (Table 1), including 257 for HLA-ABC (from 87 source proteins), 59 for HLA-DR (from 34 source proteins), 8 for HLA-DQ (from 7 source proteins), and 1 for HLA-DP.

## DISCUSSION

To survey self-antigens potentially recognized by autoimmune T cells in T1D, we used a model system in which human pancreatic islets were cultured *in vitro* with inflammatory cytokines to simulate *in vivo* conditions in an inflamed pancreas. The treatment upregulated the expression of MHC-I and MHC-II proteins and their associated antigen-processing machinery. Single-cell and bulk transcriptomic analyses showed that the *in vitro* treatment induced similar β cell expression changes as previously observed *in vivo* for T1D patients compared with non-diabetic controls.^19^ Among the MHC-I and MHC-II bound peptides from treated islets, we found 6 epitopes already known to be targeted by T cells in T1D and 28 new epitopes from proteins previously reported to be sources of T1D epitopes (Table 1). We identified a set of >900 new potential autoimmune epitopes from >400 source proteins not previously shown to be targeted by autoimmune T cells in T1D, based on preferential or exclusive expression in pancreas, upregulation by cytokine treatment, or upregulation in T1D patients (Figures 4A and 4B; Table S8). To evaluate whether autoimmune T cells targeted these epitopes, we tested a subset for recognition by T cells in peripheral blood from partially HLA-matched T1D patients. Autoimmune CD4 or CD8 T cells from T1D donors recognized 28 of the peptides, validating these as new autoimmune epitopes (Figures 5 and 6). Studies with T cells found infiltrating human islets in organ donors with recent onset T1D identified an additional four new autoimmune T cell epitopes from previously identified T1D-associated antigens including one carrying post-translational modifications. We identified an additional 100 candidate T1D-associated epitopes, selecting for peptides derived from secretory hormones, hormone processing proteins, antigen-processing and presentation components, proteins induced by oxidative stress or unfolded protein response pathways, and proteins that are known antibody or T cell targets in other autoimmune or auto-inflammatory conditions (Table 1). These results identify novel naturally processed islet peptides targeted by autoimmune T cells in T1D and provide a resource for investigating T1D etiology and progression.

These studies support the idea that inflammatory processes in pancreatic islets can break tolerance to self-antigens. Nineteen of the 28 newly discovered T1D autoantigens for which we observed T cell responses correspond to genes upregulated by treatment *in vitro* with inflammatory cytokines. Nine new epitopes derived from proteins involved in sensing or responding to inflammation (*IFT2*, *MOV10*, *IL32*, *LGALS8*, *CTSS*, *TNIP1*, *ITIH4*, *LYN*, *DUSP5*). Two new epitopes derived from proteins involved in ER stress and the unfolded protein response and mitochondrial stress (*ERN-1*, also known as *IRE-1* and *HK1*). We identified previously unreported epitopes derived from proteins involved in β cell secretory hormone synthesis, processing, or secretion (*CHGA*, *CHGB* also known as *SCG1*, *SCG3*, *SCG5*, *IAPP*, *PCSK2*, *CPA1*, *CPB1*, *CPE*, *SLC39A14*). Four additional new epitopes targeted by T cells derived from related secretory pathways not specific to islet β cells (*EHD1*, *EHD4*, *SDCBP*, *CDH1*), and several new T1D epitopes derive from proteins with no known disease-related or autoimmune function (*MYH9*, *MYL12B*, *LAMC2*, *COBL*, *FLNA*, *JAG1*) (all detailed in Table S10).

We identified 90 naturally processed peptides presented by MHC proteins in the high-risk DR3/DQ2.5 or DR4/DQ8 haplotypes and 610 additional peptides presented by the other risk-associated alleles (Table S1). All of the newly identified autoimmune epitopes recognized by peripheral T cells or islet-derived T cell lines were presented in these high-risk haplotypes or other risk-associated alleles. Because of their different peptide binding specificities, particular HLA alleles will bind different peptides from the same source proteins. We found 46 cases where naturally processed peptides (28 independent core epitopes) derived from previously identified T1D antigens were presented by different HLA alleles than those originally observed (Table 1). Similarly, we expect that HLA alleles associated with increased T1D risk will bind peptides from the many candidate autoantigens for which we identified naturally processed peptides bound to non-risk alleles, and that HLA binding predictions for risk alleles applied to the candidate autoantigens might thus provide additional candidate T1D epitopes.

In addition to the recognition of peptides derived from the immunopeptidome of cytokine-stressed islets by T cells in the periphery, we were able to examine T cell reactivity from the site of pathology in human T1D. T cell lines were derived from the islets of donors with T1D, including two donors with demise at onset of T1D and two donors with ≤1 year duration of T1D. We detected reactivity to several pools of peptides from the stressed islet immunopeptidomes predicted to bind to A*02:01 or DRB1*03:01, to individual peptides from two islet secretory hormones (*SCG5*, *CHGB*), and to two PTM neo-epitopes derived from *CHGA*, an islet prohormone and insulin packaging factor already known as a target of T cells in T1D. Our data strongly support evidence that epitopes and neoepitopes are formed in the stressed β cells from hormone secretory proteins,^13,14,16,52–56^ and we show here that these are processed and presented in the MHC immunopeptidome to become islet-derived autoreactive T cell targets, which may be major targets early in the development of T1D in human subjects. Overall, results presented in this study identify human islet peptides naturally processed and presented by MHC-I and MHC-II molecules in an inflammatory environment as predominant targets of autoimmune T cells in T1D and provide a resource for investigating T1D etiology and progression.

### Limitations of the study

There are some limitations to this study. We cannot pinpoint which epitopes were presented by which cells. Immune cells such as macrophages and dendritic cells present peptides derived from material taken up from their environment, including soluble species and membrane fragments shed by other cells. Immune cells comprise a tiny fraction of the total islet cell populations but express high levels of MHC proteins and likely represent a substantial fraction of the overall antigen presentation capacity, particularly for MHC-II. In addition, we cannot rule out the minor contribution of carried-over acinar tissue. Because we needed to match HLA alleles between organ donors whose pancreata were used for immunopeptidome studies and patients with T1D whom PBMC were obtained, only five HLA alleles were evaluated in our T cell studies. Our immunopeptidome studies covered many other HLA alleles, and most of the candidate self-antigens identified in this study remain to be tested. We did not search comprehensively for defective ribosomal products, unconventional open reading frames, or spliced and hybrid peptides. Many aspects of the biology and immunology of a diseased pancreas likely were not captured by our *in vitro* model, which uses an artificial means of creating an inflammatory environment and only a single 48-h time point to model the changing inflammatory conditions during T1D development and progression. Sensitivity of the mass spectrometry analysis is limited, and it is possible that peptides able to elicit T cell responses were present in eluted peptide samples but not detected because of abundance below detection limits. Finally, a high background of peptide byproducts from physiological proteolytic processing of secretory hormones limited our ability to identify new antigens from these proteins.

## RESOURCE AVAILABILITY

### Lead contact

Requests for further information and resources should be directed to the lead contact author, Lawrence J. Stern (lawrence.stern@umassmed.edu).

### Materials availablility

This study did not generate new unique reagents.

### Data and code availability

- All bulk and single-cell RNA-seq data are available in the Gene Expression Omnibus (GEO) repository accession numbers GEO:GSE255255 and GEO:GSE254985. Mass spectrometry data are available from the MassIVE repository database identifier MassIVE:MSV000093843.
- This paper does not report original code.
- Any additional information required to reanalyze the data reported in this paper is available from the lead contact upon request.

## STAR★METHODS

### EXPERIMENTAL MODEL AND STUDY PARTICIPANT DETAILS

#### Pacreat islets

Islet equivalent cells (IEQ) cells or spleen tissues were procured from Prodo laboratories and Alberta Diabetes Institute (ADI) IsletCore.

#### Human subjects

Whole blood was obtained from donors in a study approved by the UMass Chan Institutional Review Board. Informed consent of each donor was obtained prior to participation. Demographics of the participants are provided in Table S1.

### METHOD DETAILS

#### *Ex vivo* islet culture and flow cytometric analyses of islet cell populations

Islet equivalents were cultured for 48hrs in AIM-V + 10% human serum with 50 ng/mL IFNγ, 5 ng/mL IL-1β, 10 ng/mL TNFα. The spleen tissue was dissociated into single-cell suspensions by treatment with collagenase type II enzyme (Sigma-C6885), in order to facilitate separation from extracellular matrix and other unwanted tissue-material. Aliquots of both non-treated and cytokine-treated islets were removed from cultures at 48 h and stained for CD45 and intracellularly for hormones.^19,58^ Briefly, islets were dispersed with recombinant trypsin (TryPLE Express, ThermoFisher) at 1X for 10 min at 37°C with occasional shaking. After washing, islet dispersed cells were stained with stained for viability (LIVE/DEAD Fixable Aqua Dead Cell Stain Kit; Thermo Fisher Scientific), blocked with 50% human sera, and surface stained for CD45-APC-H7 (clone 2D1; BD Biosciences) and anti-HLA-DR-Alexa Fluor 700 (clone L243, BioLegend). After washing, cells were fixed, permeabilized, and stained with anti-insulin Alex Fluor 647 (C27C9; Cell Signaling Technology), anti-glucagon (K79bB10; Sigma-Aldrich) labeled with Zenon Alexa Fluor 488 (Thermo Fisher Scientific). FMO (Fluorescence Minus One) controls were used to determine gates for each stain. Cells were run on a BD LSRII cytometer (BD Biosciences) and analyzed with FlowJo (version 10.3) software. Results were similar for islets from donors HP-20289-1 (Figure 1) and from donors HP-18081-01 and HP-18076-01 (not shown).

#### RNA-Seq library construction, sequencing, gene expression, and statistical analysis

Dissociated islets were stained with Zombie Violet (Biolegend), fixed, permeabilized, and stained for insulin, glucagon, somatostain, and CD45. RNA was isolated from sorted populations (RecoverAll, Thermo Fisher) as described.^19^ Bulk RNA-Seq libraries were constructed using the SMARTer Stranded Total RNA-Seq Kit (Pico Input Mammalian, Takara) and sequenced as 42bp paired-end reads on a NextSeq 500/550 (Illumina). We processed bulk sequence data essentially as described.^19^ Samples were processed for single cell RNA-Seq using 10X Genomics 3′ NextGEM 3′ Single Cell kits and Chromium controller per the manufacturer’s protocol. The cDNA libraries were prepared using the 10X Library prep kit (Next GEM Single Cell 3′ Library Kit v3) and sequenced with the Illumina NextSeq 500/550. Resulting paired-end fastq files were processed through DolphinNext^59^ single-cell RNA seq pipeline. Reads from cells with less than 500 reads were removed. The mRNA reads were aligned to human genome (hg38v34) using tophat2 (v2.0.12) with default settings. ESAT (https://github.com/garber-lab/ESAT) was used to quantify gene transcripts, using RefSeq transcript annotations. Individual samples were transformed into Seurat^60^ objects for cell type calling. Dead cells (>0.25 mitochondrial gene expression) and doublets (scDoubleFinder^61^) were removed before identifying cell types. Clusters were determined by following the protocols in the Seurat vignettes, with defaults changed to fit our datasets. The unique molecular identifier (UMI) cutoff was set to >500 to ensure we captured the less transcriptionally active immune cells in the islets.

To identify islet cell types, we used ClustifyR^62^ with the Baron et al. human islet scRNA-seq dataset^63^ as the reference. For identification of immune cells, we used CellTypist^64^ with their human immune cell model. We confirmed the identified cell types’ of gross features closely matched well-known cell type-restricted gene expression. This includes major secretory hormone genes (*GCG*, *INS*, *SST*, *PPY*, and *GHRL*), acinar specific genes (*PRSS1* and *PNLIP*), and ductal specific genes (*KRT19*, *SPP1*). Multiple donors were combined to create a more robust dataset to investigate. Once combined, we used Harmony^65^ to correct for donor-to-donor variability. CELLxGENE^66^ was used to further investigate the resulting dataset. The HPAP scRNA-Seq dataset^23–25^ was downloaded from PancDB (https://hpap.pmacs.upenn.edu). The beta cell transcripts from six donors with T1D were then isolated and compared to our current control samples’ beta cells for differentially expressed genes using Seurat’s FindMarkers function. HPAP donor samples used are in Table S3.

#### Isolation of MHC-Class I and II peptides from pancreatic islets and spleen tissue samples

MHC peptide complexes were isolated using immunoaffinity chromatography; donor samples used are designated in Table S3. Cell pellets were resuspended in 5% β-octylglucoside, 50 mM Tris-HCl pH 8.0, 150 mM NaCl, containing protease inhibitor cocktail (Sigma-P2714) freeze-thawed for 5–6 times. The lysate was spun at 4000×g for 5 min at 4°C. to remove the cellular debris. The supernatant was collected and further spun using ultracentrifuge at 100,000×g for 1 h at 4°C. The supernatant was used for the isolation of the MHC-I and MHCII-peptide complexes using series of antibody-conjugated immunoaffinity columns. The supernatant was precleared by two step process, firstly incubating the supernatant with protein-A agarose beads and then with isotype control conjugated beads slowly on shaker for 1 h at 4°C to prevent nonspecific binding of proteins to beads. This equilibrated lysate was sequentially incubated starting with LB3.1-conjugated beads (anti-HLA-DR) followed by SPVL3-conjugated beads (anti-HLA-DQ), B7/21-conjugated beads (anti-HLA-DP) and then W6/32-conjugated beads. W6/32 binds classical MHC-I molecules (HLA-A, -B, - C) as well as non-classical MHC-I molecules (HLA-D, -E, -F, - G, -H), but we were able only to confidently assign peptides to the much more abundant classical MHC-I molecules. The lysate was allowed to mix slowly for 2 h each on shaker at 4°C. The column was washed by passing several buffers in succession as follows: (1) 50 mM Tris-HCl, 150 mM NaCl, pH 8.0, containing protease inhibitors and 5% β-octylglucoside (5 times the bead volume); (2) 50 mM Tris-HCl, 150 mM NaCl, pH 8.0, containing protease inhibitors and 1% β-octylglucoside (10 times the bead volume); (3) 50 mM Tris-HCl, 150 mM NaCl, pH 8.0, containing protease inhibitors (30 times the bead volume); (4) 50 mM Tris-HCl, 300 mM NaCl, pH 8.0, containing protease inhibitors (10 times the bead volume); (5) 1X PBS (30 times the bead volume); and (6) HPLC water (100 times the bead volume). MHC-peptide concentration in the lysate and the flow-through post immunoaffinity was measured by ELISA. The flow through samples had no detectable HLA-DR, with limit of detection 0.5 ng. MHC-peptide complexes were eluted from the resin using 2% TFA (The Nest Group, USA). Eluted peptide mixtures were then separated from MHC proteins, residual detergent, and cellular material by binding to a Vydac C18 spin column and eluting with 30% acetonitrile containing 0.1% (v/v) TFA. Solvent was removed by Speed-Vac and the dried peptide extracts were stored at −80°C or used immediately.

#### Mass spectrometry

Peptide extracts were reconstituted in 7 μL 5% acetonitrile containing 0.1% (v/v) trifluoroacetic acid and separated on a nano-ACQUITY (Waters Corporation, Milford, MA) UPLC with technical triplicate injections. In brief, a 5.0 μL injection was loaded in 5% acetonitrile containing 0.1% formic acid at 4.0 μL/min for 4.0 min onto a 100 μm I.D. fused-silica precolumn packed with 2 cm of 5 μm (200Å) Magic C18AQ (Bruker-Michrom, Auburn, CA) and eluted using a gradient at 300 nL/min onto a 75 μm I.D. analytical column packed with 25 cm of 3 μm (100Å) Magic C18AQ particles to a gravity-pulled tip. The solvents were A) water (0.1% formic acid); and B) acetonitrile (0.1% formic acid). A linear gradient was developed from 5% solvent A to 35% solvent B in 90 min. Ions were introduced by positive electrospray ionization via liquid junction (two samples) using Q Exactive Orbitrap Mass Spectrometer (Thermo Fisher Scientific) and four samples were analyzed using Orbitrap Fusion^™^ Lumos^™^ Tribrid^™^ Mass Spectrometer (Thermo Fisher Scientific), with greater numbers of peptides detected by the more sensitive Lumos instrument, particularly for the lower-abundance islet samples.Mass spectra were acquired over m/z 300-1750 at 70,000 resolution (m/z-200), and data-dependent acquisition (DDA) selected the top 10 most abundant precursor ions in each scan for tandem mass spectrometry by HCD fragmentation using an isolation width of 1.6 Da, collision energy of 27, and a resolution of 17,500.

#### Data processing and identification of peptides

The raw data files were peak processed with Proteome Discoverer (version 2.1, Thermo Fisher Scientific) prior to database searching with Mascot Server against the database of UniProt_Human downloaded on 08/05/2016 and 04/09/2019. Scaffold was used to assign probabilities using PeptideProphet or the LDFR algorithm for peptide identification and the ProteinProphet algorithm for protein identification, allowing the peptide and protein identification to be scored on the level of probability. Search parameters included “no enzyme” specificity to detect peptides generated by cleavage after any residue. The variable modifications of oxidized methionine and pyroglutamic acid for N-terminal glutamine were considered. The mass tolerances were 10 ppm for the precursor and 0.05Da for the fragments. Search results were then loaded into the Scaffold Viewer (Proteome Software, Inc., Portland, OR) for peptide/protein validation and label-free quantitation. Scaffold assigns probabilities using Peptide Prophet or the LDFR algorithm for peptide identification and the Protein Prophet algorithm for protein identification, allowing the peptide and protein identification to be scored on the level of probability. Eluted peptide samples were subjected to liquid chromatography with tandem mass spectroscopy (LC/MS/MS) analysis using a data-dependent acquisition strategy with fragmentation patterns matched against those predicted for the human Swiss-Prot database. A false-discovery rate (FDR) threshold of 1% was adjusted for reliable identification of peptides. Likely contaminants such as keratins, immunoglobulins, and actin were removed from the list of the eluted peptides. We also removed the peptides eluted from protein A-only and isotype-control antibody-conjugated beads, as well as peptides overlapping with these with sequences by 6 or more amino acids. Peptides with 8–13 amino acid residues were considered for MHC-I epitopes and peptides from 12–30 residues for MHC-II. MHC-II peptides overlapping by at least 6 residues were examined to determine if they were presented at part of a nested set.

#### Spliced and hybrid peptides

We followed the hybrid insulin peptide library approach used previously for screening of the epitopes for diabetogenic murine CD4^+^ T cells,^39,40^ in which N- and C-terminal fragments of insulin granule peptides are combinatorially combined. The fusion database was created from N-terminal fragments hEGGG (FYTPKTRREAEDLQVGQVELGGGPGA), hEL (LQVGQVELGGGPGAGSLQPLALEGS), InsB1-23 (AAAFVNQHLCGSHLVEALYLVCGERGF), hChgA-1 (PMPVSQECFETLRGDERILSIL), hChgA-2 (ILSILRHQNLLKELQDLALQGA), and C-terminal fragments ChgA-WE14 (SKRWSKMDQLAKELTAEKRLEGQEEE), ChgA-LF19 (EKRLEGQEEEEDNRDRSSMKLSFRAR), C-peptide1 (TRREAEDLQVGQVELGGGPGAGSLQ), C-peptide2 (GQVELGGGPGAGSLQPLALEG), A-chain (QKRGIVEQCCTSICSLYQLENYCN), IAPP1 (LKATPIESHQVEKRKCNTATCATQR), IAPP2 (GKRNAVEVLKREPLNYLPL), SCG1 (EKRFLGEGHHRVQENQMDKARRHPQ), NPY (GKRSSPETLISDLLMRESTENVPRT). We created the fusion variants by trimming 1–6 residues from the C-terminal ends of the N-terminal fragments, and 1–6 residues from the N-terminal ends of the C-terminal fragments, before fusing each N-terminal variant to every C-terminal variant. This database of 2205 notional fusion sequences were then merged with the human database for immunopeptidome searches.

#### Identification of post-translationally modified peptides

The MHC-I and MHC-II eluted islets and spleen samples were analyzed for post-translationally modified peptides using Peaks Xpro and 11 software (Bioinformatics Solutions, Waterloo, Canada), by searching against the human (*Homo sapiens*) Swiss-Prot database (51,800 entries, October 2023) and enabling chimera and deep learning boost searches in the built-in algorithm. The following search parameters were applied for the MS/MS analysis: no enzyme digestion, a parent mass tolerance set to 15 ppm using monoisotopic mass, and fragment ion mass tolerance set at 0.05 Da. The analysis of amide redox PTMs and other PTMs related to oxidative stress focused on the reported chemical moieties which were specified as variable modifications. The reported proteins, peptides and modified-peptides with redox PTMs were validated using the false discovery rate (FDR) method built in PEAKS Xpro and PEAKS 11 where protein identifications were accepted if they could be identified with a confidence score (−10logP) > 15 for peptides and (−10logP) > 15 for proteins; a minimum of 1 peptide per protein after data were filtered for less than 3.0% FDR for peptides and less than 4% FDR for proteins identifications. Using the site localization built-in algorithm PEAKS PTM re-analyzed MS/MS spectra identified as modified peptides and calculated the “A-score” values and site localization probabilities to assess the level of confidence in each PTM localization. The identified PTM with A-scores ≥13 (corresponding to a statistical significance of *p* ≤ 0.05 for the probability of site localization) were used to construct pie charts describing the % frequency for each PTM from the total identified PTMs in each sample set. In addition, selected peptides having redox PTMs were manually inspected for their corresponding MS/MS spectra. The redox PTM-amide modified peptides were manually inspected as previously described^67–70^ using the following criteria: 1) missed cleavage at the modified residue; 2) the accurate mass increase corresponding to redox-PTM modification; 3) presence of fragment ions retaining modification; 4) if modification is at the N terminus, then presence of b-ions retaining modification and unmodified y-ions; 5) if modification is at the middle position, then presence of b- or y-ions retaining modification; 6) presence of at least a few (2 or 3) consecutive b- or y-ions; and 7) presence of complementary b- or y-ions.

#### Identification of candidate MHC presenting elements

The eluted peptides were further filtered for selecting the MHC peptides. Firstly, the peptide lists were filtered to remove contaminants such as keratins and IgG-derived peptides. Secondly, the peptides identified in the beads only or isotype antibody conjugated beads column, if present in HLA-DR, DQ, DP and HLA-ABC were removed from the peptidome. Also, any peptides overlapping with ≥6aa with the peptides eluted from beads only or isotype antibody conjugated beads are removed. Thirdly, the length cut off was used for the HLA-ABC peptides as 8–13aa and 12–30aa for HLA-DR, DQ and DP peptides.

#### Assigning MHC-I and MHC-II eluted peptides to alleles

HLA-peptide binding prediction was performed with NetMHCpan4.1 or NetMHCIIpan4.2 for peptides eluted from MHC-I (HLA-ABC) and MHC-II (HLA-DR, DQ and DP) proteins, respectively for each of the HLA alleles present in each sample (Table S1). Rank percentiles cutoffs of ≤2% and ≤5% were used for assigning MHC-I and MHC-II alleles, respectively. For peptides for which there was not an allele predicted with these thresholds, the cutoff was extended to ≤10%. The rest are left unassigned. Peptides eluted from non-classical MHC-I proteins HLA-E and HLA-G might be present in the eluted peptide mixtures but were not able to confidently assign these alleles to any of the eluted peptides.

#### HLA-A2:01 and HLA-DRB1*03:01 peptide bibding exchange asaay

A fluorescence polarization competition binding assay was used to calculate the binding affinity of the proposed peptides to HLA-A*02:01 or DRB1*03:01. HIV-RT (ILKEPVCGV) and were labelled with Alexa Fluor 488 C5-maleimide and used as the probe peptide for A*02:01 binding assay. Hsp65 (KTIAYDEEARR) labeled with Alexa Fluor 488 tetrafluorophenyl ester (Invitrogen, Carlsbad, CA) via the primary amine at K_1_ as probe peptide. Alexa 488 labelled HIV-RT peptide and unlabelled peptides for these studies were obtained from 21st Century Biochemicals (Marlborough, MA) and AssayQuant Technologies (Marlborough, MA). The binding reactions for A02:01 were carried at 4°C in buffer conditions of 1X PBS, 0.1% β-octylglucoside, 1X protease cocktail inhibitor and HLA-DRB1*03:01 were carried out at 37°C in 100 mM sodium citrate, 50 mM sodium chloride, 0.1% octyl β-D-glucopyranoside, 5 mM ethylenediaminetetraacetic acid, 0.1% sodium azide, 0.2 mM iodoacetic acid, 1 mM dithiothreitol. The empty DRB1*03:01 and A2*01:01 refolded with RT peptide were used to calculate the binding affinities. The DRB1*03:01 and A2*01:01 concentration used was selected by titrating the proteins against fixed labelled peptide concentration (25 nM) of Hsp65 or HIV-RT peptide respectively. We chose the concentration of the proteins for the assay that showed ∼50% of maximum binding. IC50 values were calculated by incubating 0.5μM of A2 protein and 0.2μM of HLA-DRB1*03:01 with their respective Alexa488 labelled peptides in presence of 5-fold dilution of the test peptides starting from 40μM to 0.5μM. The capacity of each test peptide to compete for binding of probe peptide was measured by FP after 16hrs at 4°C for HLA*A2:01°C and 37°C for HLA-DRB1*03:01. The assay was read using a SpectraMax plate reader (Molecular Devices). FP values were converted to fraction bound by calculating [(FP_sample - FP_free)/(FP_no_comp - FP_free)], where FP_sample represents the FP value in the presence of test peptide; FP_free represents the value for free Alexa 488-conjugated peptide; and FP_no_comp represents values in the absence of competitor peptide. We plotted fraction bound versus concentration of test peptide and fit the curve to the equation y = 1/(1 +[pep]/IC50), where [pep] is the concentration of test peptide, y is the fraction of probe peptide bound at that concentration of test peptide, and IC50 is the 50% inhibitory concentration of the test peptide.

#### Derivation of T cell lines from peripheral blood

Whole blood from HLA-typed T1D donors was collected under a protocol approved by the UMass Chan Medical School Institutional Review Board of the University of Massachusetts, and informed consent was obtained from all subjects. Peripheral blood mononuclear cells (PBMCs) were isolated using Ficoll-Paque (Cytiva, Marlborough, MA) density gradient centrifugation, washed twice with PBS, and resuspended in CST^™^ OpTmizer T cell medium (Gibco, Grand Island, NY). Cells required for assay were removed, and the remaining cells were cryopreserved in CryoStor CS10 media (SIGMA-ALDRICH). Peptide pool-specific T-cell lines (TCLs) were generated by a single *in vitro* expansion of CD4 and CD8 cells, which were positively selected from fresh PBMCs using EasySep Human CD4 and CD8 Positive Selection kit II (STEMCELL Technologies, Cambridge, MA), using pools of eluted MHC-I or MHC-II peptides. Autologous T-cell-depleted PBMCs were used as a source of antigenpresenting cells. Briefly, 2 × 10^6^ irradiated T-cell-depleted PBMCs were dispensed with 3–4 × 10^6^ positive-selected CD4 or CD8 cells per well (24-well plate) in 1 mL of cRPMI-1640 (RPMI 1640 supplemented with 10% AB + human serum (GeminiBio, West Sacramento, CA), 50 μM beta-mercaptoethanol, 1 mM non-essential amino acids, 1 mM sodium pyruvate and 100 U/mL penicillin and 100 mg/mL streptomycin (all Gibco, Grand Island, NY). Corresponding DR-DQ (CD4) and A2 (CD8) peptide pools were added to wells at a final concentration of 2.5 μg/mL of each peptide in the pool. After 3 days, CD4 TCLs were supplemented with recombinant human 100 U/mL IL-2 (Proleukin, Prometheus, San Diego, CA) and CD8 TCLs with 20 ng/mL IL-7 (Peprotech, Cranbury, NJ) and 100 U/mL IL-2. During the following 2–15 days, one-half of the medium was replaced with fresh CRPMI supplemented with 100 U/mL IL-2 every 3 days.

#### Human islet-derived T cell lines

Live pancreas slices (150–200 μm thick) were provided by nPOD. Donors with T1D 6550, 6551, 6578, and 6579 are described in Table S1; all donors had <1 year duration of T1D and with demise at T1D onset for donors 6550 and 6578. Ten slices were digested with 1 mg/mL collagenase-P (Sigma) in PBS at 37°C with shaking with a magnetic stir bar, with tissue dispersal visually monitored. After washing, islets were enriched by hand-picking, and single-islet-derived T-cell lines generated as described^51^ with modifications. Single islets were plated in wells of 96 well round bottom plates (CoStar) on irradiated (5000 rads) allogeneic PBMC with AIM-V media (Thermo Fisher Scientific) supplemented with 2 mM L-glutamine, 5 mM HEPES, and 100 U/ml penicillin and 100 μg/mL streptomycin, 0.1 mM each non-essential amino acids, 1 mM sodium pyruvate, and 5% heat-inactivated human male AB serum (Grifols Diagnostic) with 5 μg/mL each of anti-CD3, anti-CD28 (BioLegend), anti-PD-1 (ligand blocking clone EH12.1, BD Biosciences) and anti-Fas (clone SM1/23: Thermo Fisher Scientific) with 20 U/ml IL-2, and 10 ng/mL IL-15 (R&D), and 50 nM mifepristone (Thermo Fisher Scientific). The lymphocytic outgrowth from individual islets was collected, re-plated, allowed to expand with IL-2 and IL-15, and cryopreserved in early passage (P1-P3). For these experiments, 7–10 T cell lines derived from single islets from each donor were combined. Each combined T cell line contained either >60% CD4 or CD8 T cells. For some experiments in order to enriched for any reactive T cells, combined islet-derived T cell lines were stimulated with individual peptides pools with the inclusion of 5 μg/mL soluble anti-CD28 (BD Biosciences), fed with 20 U/mL recombinant IL-2 (Proleukin) and recombinant 10 ng/mL IL-15 (R&D Systems) on Day 1, allowed to rest for 4–6 days, and then re-tested for reactivity to that peptide pool.

#### T cell response to selected candidate epitopes

IFN-γ ELISpot were performed using Human IFN gamma ELISpot KIT (Invitrogen, San Diego, CA) and MultiScreen Immobilon-P 96 well filtration plates (EMD Millipore, Burlington, MA), following manufacturer’s instructions. Assays were performed in CST^™^ OpTmizer T cell medium (Gibco, Grand Island, NY). Peptides or peptides pools were used at a final concentration of 2.5 μg/mL per peptide; as negative controls were used DMSO (DMSO, Fisher Scientific, Hampton, NH) and a pool of human self-peptides,^6^ and as a positive PHA-M (Gibco, Grand Island, NY). For assay with peptide in-vitro expanded cells, 5x10^4^ cells per well were incubated with 1X10^5^ irradiated (3500 rads) autologous PBMCs in the presence of peptides or controls for ∼22 h. For assays with islet-derived T cell lines, 5 × 10^4^ T cells per well were incubated with 1x10^5^ irradiated K32 cells expressing HLA A*02:01 cells, respectively. Peptides and PHA were tested in duplicates and six to ten DMSO controls were included for each line. Secreted IFN-γ was detected using 3-Amino-9-ethylcarbazole (AEC)/H_2_O_2_ substrate (Alfa Aesar, Haverhill, MA). Plates were analyzed using the CTL ImmunoSpot Image Analyzer (ImmunoSpot, Cleveland, OH) and ImmunoSpot 7 software. For islet-derived combined T cellana lines, either irradiated A*02:01-Hmy.2B-LCL or DRB1*03:01-expressing QBL B-LCL (Millipore Sigma) were pulsed for 2 h with indicated pools of peptides (each peptide at 2.5 μg/mL), washed and then co-cultured at 2x10^4^/cells well with combined T cell lines at 5x10^4^/cell in 96-well round bottom plates (CoStar) in 5%, heat-inactivated 5% human male AB serum (Grifols Diagnostic) in AimV (ThermoFisher) supplemented with 10 mM HEPES buffer, 2 mM L-glutamine, and 100 U/100 μg/ml penicillin/streptomycin (ThermoFisher) and 2.5 μg/mL soluble anti-CD28 (BD BioSciences). Control peptides are indicated in the figure legend. On Day 1, 20 U/mL recombinant IL-2 (Proleukin) and recombinant and 10 ng/mL IL-15 (R&D Systems) was added to all conditions. Supernatants were collected at 48 h and analyzed with multiplexed enhanced sensitivity cytokine bead arrays (BD BioSciences) for IFNγ, TNFα, and IL-10, each with a sensitivity of 274 fg/mL.

### QUANTIFICATION AND STATISTICAL ANALYSIS

Peptide identifications were done by Scaffold by assigning probabilities using PeptideProphet or the LDFR algorithm and peptides were filtered with 1% false-discovery rate. In RNA sequence analysis, multiple corrections using Benjamini-Hochberg equation was used to calculate the adjusted *p* values to identify differentially upregulated and downregulated transcripts which are ≥ 2-fold and *p* ≤ 0.05. Unpaired T test was used to calculate the predicted binding affinity differences between islets and spleen immunopeptidome in Figure 2E. *p*-values less than 0.05 were considered significant. ELISpot statistical analysis was performed using a distribution-free resampling (DFR) algorithm described by Moodie et al.,^50^ available at https://rundfr.fredhutch.org, in which a permutation (bootstrap) test is combined with a method that introduces adjustment for multiple comparisons (Westfall-Young method). The null hypotheses tested are that the mean of experimental wells is less than or equal to the mean of control wells (DFR1x) or twice the mean of control wells (DFR2x). DFR2x is very sensitive to detect intermediate to high positive responses while maintaining a low false positive rate.^50^ Error bars in plots show standard deviations. Responses detected by cytokine bead array were analyzed with paired parametric Student’s t-tests (GraphPad Prism version 10.2.1).

## Supplementary Material

1

2

3

4

5

6

7

8

9

Supplemental information can be found online at https://doi.org/10.1016/j.celrep.2025.115927.

## Figures and Tables

**Table 1.** Islet eluted peptides that are previously reported T cell epitopes, new epitopes from known autoantigens, or new candidate and confirmed epitopes

Protein	Gene	Position^A^	Peptide^B^	HLA^C^	IEDB^D^	Notes
Previously reported epitopes						
Insulin (PPI)	*INS*	34–42	HLVEALYLV	A2.5^J^ (A2), A24^I^	100920	
		15–24	ALWGPDPAAA	A2^I^ (A2)	103041	
Chromogranin-A	*CHGA*	381–390	YGFRGPGPQL	B49 (C*03:04)	952613	
		383–390	FRGPGPQL	A2.5^J^ (C*07:01)	952110	
Neuroendocrine convertase 2 (PC2)	*PCSK2*	30–38	FTNHFLVEL	A2.5^J^ (A2), C7, C12.2	952113	
Heat shock 70 kDa protein 1B (Hsp70)	*HSPA1B*	430–447	TYSDNQPGVLIQVYEGER	DQ6 (N/A)	904584	
		430–446	TYSDNQPGVLIQVYEGE		-	
		431–446	YSDNQPGVLIQVYEGE		-	
		431–445	YSDNQPGVLIQVYEG		-	
		432–446	SDNQPGVLIQVYEGE		-	
		432–445	SDNQPGVLIQVYEG		-	
		433–447	DNQPGVLIQVYEGER		-	
		433–446	DNQPGVLIQVYEGE		-	
New MHC-I peptides from known autoantigens						
**Chromogranin-A** ^E,F^	***CHGA***	2–10	**RSAAVLALL** ^E,F^	A2.5^J^, C12.2	952358	
Heat shock 70 kDa protein 1B (Hsp70)	*HSPA1B*	129–137	EIAEAYLGY	A26	443449	
		162–170	GVIAGLNVL	A2.5, C12.2	466134	
		329–337	AQIHDLVLV	A2.5^J^, B49, B52	1001777	
		341–349	TRIPKVQKL	*C4*	435773	
		423–431	KQTQIFTTY	*A26, B15, B35*	506502	
Endoplasmic reticulum chaperone BiP (GRP78)	*HSPA5*	157–165	AEAYLGKKV	B49, B52	198721	
60 kDa heat shock protein (Hsp60)	*HSPD1*	63–74	IEQSWGSPKVT	-	1101645	
Insulin (PPI)	*INS*	22–30	AAAFVNQHL	A2.5^J^, B52, C7, C12.2	105639	
		*32–40*	GSHLVEALY	*A31, B35*	174524	
Kinesin-like protein	*KIF1A*	219–228	AVFNIIFTQK	A3, A11	614495	
		860–868	LLYPVPLVH	A3	621149	
Receptor-type N tyrosine-protein phosphatase-like N (IA-2)	*PTPRN*	75–83	VTSPVLQRL	A2.5^J^, B52, C7, C12.2	-	
		436–444	ARPPVTPVL	*C4*	-	
		437–445	RPPVTPVLL	B7.2^K^, C7.2	-	
		965–975	AVAEEVNAILK	A11	442966	
		96–107	AELTGDNIPKDF	B44.2	-	
Neuroendocrine protein 7B2 (Secretrogranin 5)	*SCG5*	90–98	NIPNIVAEL	A2.5^J^, C12.2	-	
New MHC-II peptides from known autoantigens						
**Chromogranin-A** ^E,G^	***CHGA***	198–222	QAEGDSEGLSQGLVDREKGLSAEPG	DR1	–	
		198–225	QAEGDSEGLSQGLVDREKGLSAEPGWQA	DQ6, DQ6.2	–	
		198–224	QAEGDSEGLSQGLVDREKGLSAEPGWQ	DQ6, DQ6.2	–	
		207–224	SQGLVDREKGLSAEPGWQ	DR1	–	
		243–261	AVPEEEGPTVVLNPHPSLG	DR, DQ	–	
		243–261	**AVPEEEGPTVVLXPHPSLG**^E,G^ (X=deamindated N=asp)		–	
		243–261	**AVPEEEGPTVVLXPHPSLG**^E,G^ (X=deamindated N=isoasp)		–	
Insulin (PPI)^E^	*INS*	32–48	GSHLVEALYLVCGERGF	DR3^H^	-	
		32–48	GSHLVEALYLVXGERGF (X=carboxymethylcysteine)	DR3^H^	-	
Heat shock 70 kDa protein 1B (Hsp70)^E^	***HSPA1B***	359–374	**LNKS** **INPDEAVAY** **GAA** ^E^	DR52^I^, DR3^H^	240005	
Endoplasmic reticulum chaperone BiP	*HSPA5*	61–77	ITPSYVAFTPEGERLIG	DR15.2, DR51, DR51.2	519299	
		566–581	ESYAYSLKNQIGDKEK	DR8, DR14.54	433887	
60 kDa heat shock protein (Hsp60)	*HSPD1*	63–74	IIEQSWGSPKVT	DR	1840318	
		75–95	KDGVTVAKSIDLKDKYKNIGA	DR1	1238365	
Islet amyloid polypeptide	*IAPP*	50–66	VHSSNNFGAILSSTNVG	DQ6	-	
		50–64	VHSSNNFGAILSSTN		-	
		51–66	HSSNNFGAILSSTNVG		-	
Neuroendocrine convertase 2 (PC2)	*PCSK2*	59–77	KLPFAEGLYHFYHNGLAKA	DR15, DR15.2	1212175	
**Neuroendocrine protein**	***SCG5***	72–87	**QS** **IEGGAHEGL** **QHLGP** ^E^	DR3	-	
**7B2 (Secretogranin 5**)^E^		96–107	AELTGDNIPKDF	DR52^I^	-	
Validated and Candidate MHC-I T cell epitopes						
**Serine/threonine-protein**	***ERN1***	37–45	**TLDGSLHAV** ^E^	A1^K^, A2^I^, C5^I^	156780	
**kinase/endoribonuclease IRE1** ^E^		808–815	ELIEKMIAM	A26, C6.2, C7.2	2011916	
**Hexokinase-1** ^E^	***HK1***	35–43	LVRLILVKM	B7.2 ^K^	899295	Diabetic macular edema,
		273–281	TLIDIMTRF	A26, C6.2, C7.2	449270	
		303–311	GELVRLILV	B49, B52	948983	Other autoimmune
						conditions
		388–397	ATLGAILNRL	A2.5^J^, A11	570946	
		850–858	**GLDRLNVTV** ^E^	A2^I^, C5^I^	193854	
**Interferon-induced protein with tetratricopeptide repeats 2** ^E^	***IFIT2***	109–117	**RLSDVQIYV** ^E^	A2^I^, C5^I^, C7	194294	Other autoimmune conditions
**Helicase MOV-10** ^E^	***MOV10***	407–416	QEDPITYKGF	B44.2	439670	
		533–541	TLVEAIKQV	A2^I^, C5^I^	459990	
		853–862	**GLDDIKDLKV** ^E^	A2^I^, C5^I^	193848	
**Dual specificity protein phosphatase 5** ^E^	***DUSP5***	337–345	**SLIGHLQTL** ^E^	A2^I^, A2.5^J^, B8, B52, C5^I^, C7, C12.2	59170	
**Isoform 2 of Protein cordon-bleu** ^E^	***COBL***	1112–1120	**ALMEAIHSA** ^E^	A2^I^, B8, C5^I^	541085	
**EH domain-containing protein 1** ^E^	***EHD1***	502–510	**ALANHLIKV** ^E^	A2^I^, C5^I^, C7	193634	
Tyrosine-protein kinase Lyn ^E^	*LYN*	153–161	FLIRESETL	A2^I^, B8, C5^I^	162212	
		313–321	EPIYIITEY	A26, B7.2^K^	615521	
		451–460	DVMTALSQGY	A26	614937	
		289–307	EEANLMKTL	B44.2	206594	
**Myosin regulatory light chain 12B** ^E, F^	***MYL12B***	23–33	**AMFDQSQIQEF** ^E, F^	*B15*	423992	
**Laminin subunit gamma-2** ^E^	***LAMC2***	243–251	AQRLDPVYF	*B15*	1189559	
		694–702	**RLDDLKMTV** ^E^	A1^K^, A2^I^, C5^I^, C7	484186	
		1107–1115	HLMDQPLSV	A2^I^, B8, C5^I^, C7	773425	
Aryl hydrocarbon receptor	*AHR*	140–148	STIQDYLGF	A26, C6.2, C7.2	884058	T1D development
Apolipoprotein L1	*APOL1*	91–100	NEAWNGFVAA	B45	950384	Genetic association with Nephropathy
		100–108	AHDLVIKSL	*C4*	698515	
		160–168	ALADGVQKV	A2.5^J^, B52, C7, C12.2	161927	
		243–251	LVIKSLDKL	A2.5^J^	162970	
Isoform 3 of Programmed cell death 1 ligand 1	*CD274*	10–18	MTYWHLLNA	A2.5^J^	635156	Cytokine stress
C-X-C motif chemokine 2	*CXCL2*	70–78	AQTEVIATL	A2^I^, B44.2, C5^I^, C7	1083741	Cytokine stress
Glutamate decarboxylase 1	*GAD1/GAD2*	573–581	FLIEEIERL	A2.5^J^, B52, C7, C12.2	104778	
Interferon-induced protein with tetratricopeptide repeats 3	*IFIT3*	109–117	RLSDAQIYV	*A2* ^J^	447475	Cytokine stress
		113–120	AQIYVDKV	B52	1001807	
Isoform 7 of Interleukin-32	*IL32*	90–100	AELEDDFKEGY	B44.2	176923	Cytokine stress
		171–179	GVLAWVKEK	A11^K^	505741	
Interleukin-1 receptor-associated kinase-like 2	*IRAK2*	604–612	KLMENILLY	A3^K^, A26, C6.2, C7.2	425831	Cytokine stress
Interferon regulatory factor 9	*IRF9*	382–390	TPEQQAAIL	*B35*	237448	Cytokine stress
Interferon-induced GTP-binding protein Mx1	*MX1*	134–141	HELITLEI	B49. B52	937354	Cytokine stress
		175–185	TLIDLPGITRV	A2.5^J^	156781	
		233–243	QEVDPEGDRTI	B49	223589	
		350–357	HQRITEEL	B52	438047	
		394–404	ETVGEEDIRLF	A26	563090	
		454–462	FETIVKQQI	B49, B52	437247	
		573–581	SMEEIFQHL	A2.5 ^J^, B52, C7, C12.2	637290	
Nuclear factor NF-kappa-B p105 subunit	*NFKB1*	520–528	YAVTGDVKM	*A26, B35*	603982	Cytokine stress
Nuclear factor NF-kappa-B p100 subunit	*NFKB2*	317–326	VSDSKQFTYY	A1^K^, C5^I^	163836	Cytokine stress
		460–468	SVIEQIVYV	A2.5^J^, A11^K^, B52, C7, C12.2	448983	
NF-kappa-B inhibitor alpha	*NFKBIA*	71–80	TEDGDSFLHL	B49	578529	Cytokine stress
		71–81	TEDGDSFLHLA	B45	1285425	
		89–97	LTMEVIRQV	A2.5^J^, B52, C7, C12.2	634562	
		169–176	TPHLHSIL	B8	487631	
		227–235	LQNPDLVSL	*A2* ^J^ *, B15*	1031318	
		242–251	DVNRVTYQGY	*A26*	953585	
		260–269	RPSTRIQQQL	B7.2^K^	576940	
NF-kappa-B inhibitor epsilon	*NFKBIE*	285–294	EVLDIQNNLY	A26*, B35*	2064579	Cytokine stress
Protein NLRC5	*NLRC5*	652–660	ATLTNILEH	A11^K^	442945	Cytokine stress
		1392–1402	DRARVLSLLEV	-	-	
		1394–1402	ARVLSLLEV	C7	452918	
Protein S100-A6	*S100A6*	66–74	QEVNFQEYV	B49, B52	483273	
Nuclear autoantigen Sp-100	*SP100*	132–140	QEYPDLIHI	A2.5^J^, B49, B52	439714	Cytokine stress
Antigen peptide transporter 2	*TAP2*	145–153	SRPDLPLLV	C6.2, C7.2	625879	Cytokine stress
		314–321	HQEVLREI	B52	1018938	
		334–343	EAVGGLQTVR	A26	759126	
		541–549	YLHSQVVSV	A2^I^, B8, C5^I^, C7	450199	
Neurosecretory protein	*VGF*	3–11	ALRLSASAL	B8	562095	Hormone processing
		197–206	LESPGPERVW	B44.2	-	
Protein disulfide-isomerase A3	*PDIA3*	16–24	LAAARLAAA	A2.5^J^	162824	Antigen processing and presentation
EH domain-containing protein 4	*EHD4*	337–345	RLPEIYIQL	A2.5^J^, B52, C7, C12.2	447461	
Syntenin-1	*SDCBP*	14–22	KVIQAQTAF	*A26, B15, B35*	446066	
		193–201	RPFERTITM	*A26, B35*	440094	
Myosin-9	*MYH9*	130–138	YSEEIVEMY	A1^K^, C5^I^, C7	474082	Other autoimmune conditions
		289–300	KTDLLLEPYNKY	-	430766	
		345–355	GVLQLGNIVFK	A11	437857	
		517–527	KPAGPPGILAL	B7.2^K^	438721	
		855–862	FTKVKPLL	B8	208959	
		877–885	QLMAEKLQL	A2^I^	51441	
		721–729	YEILTPNSI	B49, B52	885598	
		8144–1851	REGIEWNFI	B49	-	
Zinc transporter ZIP14	*SLC39A14*	60–67	QQLKALLN	ABC	-	Insulin processing
Carboxypeptidase E	*CPE*	71–79	TAISRIYTV	A2.5^J^, B52, C7, C12.2	765349	Proinsulin processing
		423–433	ASAPGYLAITK	A11^K^	934283	
Guanine nucleotide-binding protein G(s) subunit alpha isoforms XLas	*GNAS*	175–182	AQYFLDKI	B49, B52	1002102	
		98–106	NLKEAIETI	A2.5^J^, B52	635360	
		267–276	QEALNLFKSI	B49	439665	
		40–48	THRLLLLGA	ABC	728173	
		126–134	FRVDYILSV	C6.2, C7.2	419667	
		222–229	PKRLSDLL	ABC	-	
		331–339	ALDGPPIKV	A1^K^, A2^I^, C5^I^, C7	934070	
Gamma-interferon-inducible lysosomal thiol reductase	*IFI30*	213–221	HEYVPWVTV	B49, B52	1017742	Cytokine stress
		27–35	LLDVPTAAV	A1^K^, A2^I^, C5^I^	37182	
Polyadenylate-binding protein 4	*PABPC4*	631–639	KVDEAVAVL	A1^K^, A2^I^, C5^I^, C7	455883	
Isoform 3 of Protein quaking	*QKI*	217–228	APRIITGPAPVL	B7.2^K^	239455	
		44–52	LLDEEISRV	A1^K^, A2^I^, C5^I^	194143	
Multidrug resistance protein 1	*ABCB1*	442–453	RLYDPTEGMVSV	A2 ^I^	240266	
		239–247	FTDKELLAY	A1^K^, C5^I^, C7	478245	
Alpha-actinin-4	*ACTN4*	669–677	MEEIGRISI	B49	606839	
		397–405	YEEWLLNEI	B49, B52	1303377	
		555–563	FIVHTIEEI	A2.5^J^, C12.2	878268	
		531–539	HTIEEIEGL	A2.5^J^, C12.2	937670	
		782–790	AEFNRIMSL	A2.5^J^, B49, B52, C7, C12.2	474964	
		732–740	IRVGWEQLL	C6.2, C7.2	618790	
		118–127	FIASKGVKLV	A2^I^	489165	
		481–491	NELDYYDSHNV	B44.2	-	
Apolipoprotein L2	*APOL2*	213–221	ALAEEVEQV	A2^I^, A2.5^J^, C5^I^	436320	
Apolipoprotein L4	*APOL4*	128–136	VIANEIEKV	A2^I^, C5^I^	518626	
		267–275	VPINVVETL	B35	1058634	
Asparagine synthetase	*ASNS*	88–96	FEFEYQTKV	A2.5^J^, B49, B52, C7, C12.2	222302	
		221–231	KLFPGFEIETV	A2.5 ^J^	455364	
		86–94	QHFEFEYQT	ABC	720105	
		214–222	ALYDNVEKL	A2^I^, C5^I^, C7	193696	
Bromodomain adjacent to zinc finger domain protein 2A	*BAZ2A*	1024–1032	VLAFLVHEL	A2.5^J^, B52, C12.2	460311	
		1426–1435	STPLAGLAPK	A11^K^	448957	
		638–646	KQFPEVIKY	A26, B15, B35	481016	
Class E basic helix-loop-helix protein 40	*BHLHE40*	24–34	DLPGMYPAHMY	A26	562418	Cytokine Stress
		113–121	QQQQKIIAL	B8, C7	903034	
Cyclin-dependent kinase 17	*CDK17*	159–167	KLTENLVAL	A2^I^, C5^I^, C7	455571	
Guanylate-binding protein 4	*GBP4*	159–167	YVTELAELI	A2.5^J^, B52	944040	Cytokine Stress
		145–153	SVSTINHQA	A2.5^J^	967432	
		334–342	AQLENPAAV	*A24.2,* A2^I^, B15, C5^I^	504611	
		356–364	QLRLPTDTL	B7.2^K^	720413	
		377–385	EAIAVFMEH	A26, B35	641942	
		450–461	YLEEKKQVEWDY	A1^K^	433339	
GRIP and coiled-coil domain-containing protein 2	*GCC2*	532–540	ALLETVNRL	A2^I^, C5^I^, C7	442310	
		406–415	SELAGLNKQF	B44.2	458786	
Hypoxia-inducible factor 1-alpha	*HIF1A*	346–354	IQHDLIFSL	A2.5^J^, B49, B52, C7, C12.2	445219	
		58–66	SVMRLTISY	A11^K^, C7, C12.2	427588	
		766–774	MEQKTIILI	B49, B52	1257595	
		632–640	MEDIKILIA	B45	439286	
		684–695	HPRSPNVLSVAL	B7.2^K^	239787	
		804–812	APIQGSRNL	B7.2^K^	504518	
		479–487	EPNPESLEL	B35	615525	
		111–119	YISDNVNKY	A26, B15, B35	163931	
		705–713	ELNPKILAL	A2^I^, B8, C5^I^, C7	477777	
Interferon-induced protein with tetratricopeptide repeats 2	*IFIT2*	109–117	RLSDVQIYV	A2^I^, C5^I^, C7	194294	Cytokine Stress
Isoform 2 of Integrin-linked protein kinase	*ILK*	229–237	VLHEGTNFV	A2^I^, C5^I^, C7	441405	
Inositol 1,4,5-trisphosphate receptor type 2	*ITPR2*	805–813	TEIPTKITI	B49, B52	578600	
		744–752	RQYLAINQI	A2.5^J^, B49, B52, C12.2	577077	
		1976–1984	YINEKNVAL	A2^I^, B8, C5^I^, C7	732841	
Laminin subunit beta-3	*LAMB3*	1084–1092	KYAELKDRL	*C4*	1027907	
		939–947	AARLPNVDL	B7.2^K^	769963	
		1014–1022	RVAEVQQVL	A2, A26, B15, B35	458591	
		1010–1018	LIQDRVAEV	A2^I^, B8, C5^I^	775135	
Isoform 3 of Microtubule-actin cross-linking factor 1	*MACF1*	6144–6152	AEKFWYDMA	B45	496292	
		5587–5596	ALLDQALSNA	A2	452323	
		235–243	WTQKVTAGY	A26, B15, B35	652271	
		341–351	ATEVDSRWQEY	A1^K^, A44.2	162026	
		4471–4478	MSDVNLKY	A1^K^, C5	431374	
		543–551	RLQDELVTL	A2^I^, B8, C5^I^, C7	440024	
Moesin	*MSN*	131–139	TQRLFFLQV	A2.5^J^, B49, B52	-	
		554–562	HLKALTSEL	A2 ^I^, B8	211589	
Unconventional myosin-X	*MYO10*	553–560	VQYDVRGI	B52	-	
		102–110	SVNPYQPIA	*C15.2*	-	
		308–318	EVITAMDVMQF	A26	563276	
		35–44	RTDYGQVFTY	A1^K^, B44.2, C5	470157	
		1530–1538	NSDVVEQIY	A1^K^, C5	469095	
		243–251	YLLEKNRVV	A2^I^, B8, C5^I^, C7	488905	
Unconventional myosin-Ib	*MYO1B*	1008–1016	IIAEVVNKI	A2.5^J^, B52, C12.2	710780	
		288–296	AESVLAVVA	B45	933907	
		288–297	AESVLAVVAA	B45	-	
		546–555	FVDKNNDLLY	A1^K^, C5^I^	465402	
		950–958	FTDQQKLIY	A1^K^, C5^I^, C7	465358	
		269–277	YLLEKSRVV	A2^I^, B8, C5^I^, C7	17323934	
2’-5’-oligoadenylate synthase 3	*OAS3*	837–845	AEIISEIRA	B45	496288	
		494–502	AEILDEMRA	B45	933907	
		247–255	SLAEGLRTV	A2^I^, B8, C5^I^, C7	194318	
		760–769	SEFLQPNRQF	B44.2	163389	
		837–845	FVNIRPAKL	A2^I^, B8, C5^I^, C7	209055	
		494–502	DEMRAQLESW	B44.2	436821	
OTU domain-containing protein 4	*OTUD4*	568–576	HLTPSPVPV	A2^I^, C5^I^	-	
Putative transcription factor Ovo-like 1	*OVOL1*	173–181	SLLQGSPHL	A2^I^, C5^I^, C7	778146	
Proteasome subunit beta type-8	*PSMB8*	105–113	KVIEINPYL	A2.5^J^, B52, C7, C12.2, A2 ^I^, C5^I^, C7	176291	
		263–271	DVSDLLHQY	A26, C6.2	531364	
		108–117	EINPYLLGTM	A26	615421	
Proteasome activator complex subunit 1	*PSME1*	155–163	KVFELMTSL	A2.5^J^, B52, C7, C12.2	774899	Antigen processing and presentation
		224–232	AVLYDIILK	A11^K^	758463	
		135–142	LQLQIPRI	B52	1031298	
		43–52	FLKEPALNEA	A2^I^	1213294	
		190–201	KQPHVGDYRQLV	ABC	-	
Ran GTPase-activating protein 1	*RANGAP1*	205–213	RVIGTLEEV	A2^I^, A2.5^J^, B52, C5^I^, C12.2	440276	
Reticulocalbin-1	*RCN1*	21–29	VLAPRVLRA	A2^I^	489405	
Isoform 4 of Transcription factor p65	*RELA*	389–397	ALAPAPPQV	A2^I^, C5^I^, C7	452239	
E3 ubiquitin-protein ligase RNF213	*RNF213*	3446–3454	SVINEINKI	A2.5^J^, B52, C12.2	163561	
		2448–2455	NMLKILAI	B52	1035789	
		3763–3772	KIKDYLEELW	A2.5^J^	597656	
		3403–3411	TEYSFLKEV	A2.5^J^, B49, B52	578681	
		4962–4970	AEVTELHVI	B49, B52	607430	
		323–332	AVAEPANAVK	A11^K^	571005	
		2647–2654	VQRLVESI	B52	1059078	
		5228–5236	SQFPEEILL	A2.5^J^, B49, B52, C7, C12.2	821229	
		1491–1499	KVFVDLASI	A2.5^J^, B52	1106023	
		4211–4219	FITEDKTEL	A2.5^J^, C12.2	162201	
		1132–1140	AVADSVLTK	A11^K^, C7	203203	
		677–685	FVVEKIELL	A2.5^J^, B52, C7, C12.2	936560	
		2562–2570	AEDSGLHII	B49, B52	436107	
		3958–3965	AQAVIREV	A2.5^J^, B49, B52	1001591	
		5228–5237	SQFPEEILLA	A2.5^J^, B49, B52	-	
		4497–4504	IHAAAVLL	*C4*	1020629	
		4212–4220	ITEDKTELY	*B35*	162577	
		1670–1678	QAFIDLHSA	B35	719665	
		2633–2641	QLSDVAEKL	A2^I^, C5^I^	576047	
		4964–4972	VTELHVISY	A1^K^, C5^I^	163855	
Ribosome-binding protein 1	*RRBP1*	155–163	SVVNSIQVL	A2.5^J^, B52, C7, C12.2	1284095	
		975–983	ERIRSIEAL	ABC	877413	
		168–177	AILETAPKEV	A2^I^	193623	
		695–705	SEKAGIIQDTW	B44.2	219054	
Serine/threonine-protein kinase SMG1	*SMG1*	3136–3144	FTADFVRQL	A2.5^J^, B52, C7, C12.2	616550	
		36–44	SADPDNLKY	B35, C4	163373	
		515–523	LPSSFVEKL	B35	224854	
		3561–3569	KLIQKNLAT	A2^I^	-	
		1954–1962	ELRRVTVLW	B44.2	-	
Isoform 4 of Superoxide dismutase [Mn], mitochondrial	*SOD2*	80–88	AQIALQPAL	A2^I^, B15	783992	Reduced activity in T1D
Signal transducer and activator of transcription 1-alpha/beta	*STAT1*	265–273	IVAESLQQV	A2.5^J^, B52, C7, C12.2	455004	Cytokine Stress
		224–232	TELTQNALI	B49, B52	884534	
		704–712	TELISVSEV	B49, B52	1053649	
		701–709	YIKTELISV	A2.5^J^, B52, C12.2	163926	
		642–652	VTFPDIIRNYK	A11^K^	488486	
		24–33	DSFPMEIRQY	A26	562475	
		26–34	FPMEIRQYL	A26, B7.2^K^, C6.2, C7.2	437389	
		24–33	DSFPMEIRQY	A26, B35	562475	
		350–358	KLQELNYNL	A2^I^	194091	
		281–289	EELEQKYTY	B44.2	162121	
		350–358	KLQELNYNL	A2^I^, C5^I^, C7	194091	
Signal transducer and activator of transcription 2	*STAT2*	303–311	QVTELLQRL	A2.5^J^, B52, C12.2	1585180	
		698–706	YLKHRLIVV	A2^I^, B8	579866	
Signal transducer and activator of transcription 3	*STAT3*	13–21	RYLEQLHQL	*C4*	191728	T1D development
		219–227	SELAGLLSA	B45	941281	
		14–22	YLEQLHQLY	A1^K^, C5 ^I^, C7	163941	
Signal transducer and activator of transcription 6	*STAT6*	56–64	LLSDTVQHL	A2^I^, A2.5^J^, B52, C5^I^, C7, C12.2	162878	
		433–441	SEMDRVPFV	A2.5^J^, B49, B52, C7	612241	
		89–97	QRDPLKLVA	*C4*	720711	
		127–134	ELKFKTGL	B8	207358	
Transgelin-2	*TAGLN2*	142–153	GLFSGDPNWFPK	A3, A11^K^	419684	
		88–96	KQMEQISQF	A2^I^, A26, B15, B35	438790	
		88–97	KQMEQISQFL	A2.5^J^, B52	438791	
		90–99	MEQISQFLQA	B45	1257594	
		192–199	YGMPRQIL	B8	120335	
Antigen peptide transporter 1	*TAP1*	744–752	LLYESPERY	*B35*	162881	Antigen processing and presentation
		653–661	RSLQENIAY	B15, B35	440232	
		155–163	ALKPLAAAL	A2^I^, B8, C5^I^	770240	
Isoform C2 of Tight junction protein ZO-2	*TJP2*	404–412	HEGDIILKI	B49, B52	211445	
		609–617	QEGDQILKV	B49, B52	881905	
		200–210	EVMDEFDGRSF	A26	443663	
		848–857	ALLDVTPKAV	A2^I^, C5^I^	442305	
E3 ubiquitin-protein ligase TRIM22	*TRIM22*	386–394	FAFDPSVNY	A2.5^J^, A11^K^, B52, C7, C12.2	478006	
		73–81	HLANIVERV	A2^I^, C5^I^	193906	
Utrophin	*UTRN*	251–260	FEVLPQQVTI	A2.5^J^, B49, B52	936272	
		2285–2293	YVFTLAQNL	A2.5^J^, B52, C7, C12.2	1307321	
		226–237	KLLDPEDVAVQL	A2^I^	239898	
		2346–2355	EETEELMRKY	A1^K^, B44.2	206862	
mRNA decay activator protein ZFP36L1	*ZFP36L1*	63–71	HQNQLLSSL	B15	573315	
ATP-dependent DNA/RNA helicase DHX36	*DHX36*	400–408	DVIEKIRYV	A26, C6.2, C7.2	562597	
Histone-lysine N-methyltransferase, H3 lysine-79 specific	*DOT1L*	486–493	KLLESFKI	B52	-	
Glutaminyl-peptide cyclotransferase	*QPCT*	8–16	RVVGTLHLL	A2.5^J^, B52, C7, C12.2	1273764	
Ubiquitin carboxyl-terminal hydrolase 15	*USP15*	404–412	IIVDIFHGL	A2.5^J^, B52, C12.2	567793	
Glucagon	*GCG*	20–27	KEFIAWLV	B49, B52	-	Impaired release in T1D (hypoglycemia)
		2–10	KSIYFVAGL	A2.5^J^		
Pancreatic prohormone	*PPY*	95–105	AVPRELSPLDL	ABC	-	
Secretogranin-1	*CHGB*	388–396	NYPSLELDK	ABC	-	
Validated and Candidate MHC-II T cell epitopes						
**Carboxypeptidase A1** ^E^	***CPA1***	168–183	SKRPAIWIDTGIHSRE	DR3^H^, DR52^I^	-	Hormone processing
		169–183	**KRPA** **IWIDTGIHS** **RE** ^E^		-	
		168–181	KRPAIWIDTGIHSR		-	
**EH domain-containing protein 4** ^E^	***EHD4***	112–127	**TPGNA** **LVVDPKKPF** **RK** ^E^	DR3^H^, DR52^I^	556944	
		112–126	TPGNALVVDPKKPFR		-	
**Galectin-8** ^E^	***LGALS8***	87–102	**GREE** **ITYDTPFKR** **EKS** ^E^	DR3^H^, DR52^I^	773011	Immune disorders, Inflammation
		87–101	GREEITYDTPFKREK		773010	
		87–100	GREEITYDTPFKRE		1606193	
		88–102	REEITYDTPFKREKS		1268881	
**Syntenin-1** ^E^	***SDCBP***	12–30	VDKVIQAQTAFSANPANPA	DR1	233921	
		12–29	VDKVIQAQTAFSANPANP		233920	
		12–28	VDKVIQAQTAFSANPAN		233919	
		12–27	VDKVIQAQTAFSANPA		233918	
		218–233	ITSIVKDSSAARNGLL	DR3^H^, DR52^I^	429064	
		218–231	**ITS** **IVKDSSAAR** **NGL** ^E^		418843	
		25–43	NPANPAILSEASAPIPHDG	DR1, DR3^H^	462713	
**Inter-alpha-trypsin inhibitor heavy chain H4** ^E^	***ITIH4***	322–337	**WRPS** **LVPASAENV** **NKA** ^E^	DR52^I^, DR1	766589	Immune disorders
		323–337	RPSLVPASAENVNKA		764432	
		731–745	APSAILPLPGQSVER	DR1	152422	
**Myosin-9** ^E^	***MYH9***	119–133	VVINPYKNLPIYSEE	DR15, DR15.2	593264	Other autoimmune conditions
		717–731	FRQRYEILTPNSIPK	DR52^I^, DR1	531749	
		718–731	**RQR** **YEILTPNS** **IPK** ^E^		533568	
		1758–1774	QIDQINTDLNLERSHAQ	DR3^H^, DR52^I^	592789	
		1758–1773	QIDQINTDLNLERSHA		1616279	
		1759–1775	IDQINTDLNLERSHAQK		920551	
		1759–1774	IDQINTDLNLERSHAQ		518364	
		1759–1773	IDQINTDLNLERSHA		1101476	
		1759–1772	IDQINTDLNLERSH		773650	
**Cadherin-1** ^E^	***CDH1***	178–203	IKSNKDKEGKVFYSITGQGADTPPVG	DR1	-	Other autoimmune conditions
		180–203	SNKDKEGKVFYSITGQGADTPPVG		1279715	
		181–202	NKDKEGKVFYSITGQGADTPPV		-	
		183–202	DKEGKVFYSITGQGADTPPV		1196396	
		307–327	**QDPELPDKNM** **FTINRNTGV** **IS** ^E^	DR52.2	1265902	
**Carboxypeptidase E** ^E, G^	***CPE***	101–118	EPGEPEFKYIGNMHGNEA	DR15, DR15.2, DR51, DR51.2, DR1, DR1.11	423275	Autoantibodies in T1D
		425–442	APGYLAITKKVAVPYSPA	DR14.54, DR8, DR52.2	875316	
		425–439	**APGY** **LAITKKVAV** **PY** ^E, G^		10900048	
**Filamin-A** ^E^	***FLNA***	2446–2460	**NPAE** **FVVNTSNAG** **AG** ^E^	DR1.11, DR1.13, DR52^I^, DR52.2, DR52.3	152862	
		1666–1683	IGEETVITVDTKAAGKGK	DR52^I^, DR3^H^	592406	
**Cathepsin S** ^E^	***CTSS***	156–169	TGKLVSLSAQNLVD	DR1	153057	
		189–203	**FQ** **YIIDNKGID** **SDAS** ^E^	DR8, DR52.2, DR3^H^, DR52^I^	433924	
		193–208	**DNKG** **IDSDASYPY** **KA** ^E^		518336	
		193–207	IDNKGIDSDASYPYK		518335	
		194–208	DNKGIDSDASYPYKA		513309	
**Carboxypeptidase B** ^E^	***CPB1***	359–374	AGGSDDWAYDQGIRYS	DR3^H^, DR52^I^	11452201	Hormone processing
		361–373	GSDDWAYDQGIRY		1225161	
		361–374	**GSDD** **WAYDQGIRY** **S** ^E^		1225162	
		362–374	SDDWAYDQGIRYS		1274652	
**TNIP1 Isoform 7** ^E^	***TNIP1***	378–394	**ETDKEQ** **LTAEAKELR** **QK** ^E^	DR3^H^	1139654	
**EH domain-containing protein 1** ^E^	***EHD1***	109–124	**VPGNA** **LVVDPRRPF** **RK** ^E^	DR3^H^, DR52^I^	779358	
**Isoform 3 of Zinc transporter ZIP14** ^E^	***SLC39A14***	217–230	**ED** **YYVSKSAVV** **FGG** ^E^	DR52^I^, DR1	1884743	
**Interleukin-32** ^E^	***IL32***	90–102	**AE** **LEDDFKEGY** **LE** ^E^	DR3^H^	1855364	Cytokine stress
**Protein jagged-1** ^E^	***JAG1***	1167–1181	**KAR** **FAKQPAYTL** **VDR** ^E^	DR1, DR52^I^	1238064	
Apolipoprotein L1	*APOL1*	389–404	DVVYLVYESKHLHEGA	DR15, DR15.2, DR51, DR51.2	-	Genetic association with nephropathy
C-X-C motif chemokine 2	*CXCL2*	12–29	GIHLKNIQSVKVKSPGPH	DR1	-	Cytokine stress
Interferon-induced GTP-binding protein Mx1	*MX1*	442–459	GRELPGFVNYRTFETIVK	DR15, DR15.2	11322925	Cytokine stress
Protein disulfide-isomerase	*P4HB (PDIA1)*	159–170	GFFKDVESDSAK	DR	16579066	Stress response
Protein disulfide-isomerase A2	*PDIA2*	78–92	APEYSKAAAVLAAES	DR1	-	Stress response
Protein disulfide-isomerase A3	*PDIA3*	449–465	GFPTIYFSPANKKLNPK	DR51, DR51.2	496511	Stress response
Neurosecretory protein	*VGF*	64–80	NSEPQDEGELFQGVDPR	DR	12068320	Hormone processing
		405–418	LFAEEEDGEAGAED	DR	-	
		405–417	LFAEEEDGEAGAE	DR	-	
		452–468	IDSLIELSTKLHLPADD	DR14.54	11334446	
Secretogranin-3	*SCG3*	234–252	AAIQDGLAKGENDETVSNT	DR	-	Secretory hormone
		35–50	ELSAERPLNEQIAEAE	DR	12119615	
		40–52	RPLNEQIAEAEED		-	
		96–113	SSPLDNKLNVEDVDSTKN		11524397	
		96–111	SSPLDNKLNVEDVDST		-	
		133–149	DDPDGLHQLDGTPLTAE	DR	7105359	
		133–150	DDPDGLHQLDGTPLTAED		-	
Fractalkine	*CX3CL1*	59–73	GKRAIILETRQHRLF	DR3^H^	1099148	Autoimmune conditions
Stromelysin-2	*MMP10*	40–56	KYYNLEKDVKQFRRKDS	DR3^H^	-	
		40–55	KYYNLEKDVKQFRRKD		-	
		41–56	YYNLEKDVKQFRRKDS		-	
		42–54	YNLEKDVKQFRRK		-	
		42–55	YNLEKDVKQFRRKD		-	
		42–56	YNLEKDVKQFRRKDS		-	
Nucleobindin-2	*NUCB2*	25–43	VPIDIDKTKVQNIHPVESA	DR3^H^	18437134	
Reticulocalbin-1	*RCN1*	179–192	DLNGDLTATREEFT	DR3^H^	550810	
		179–191	DLNGDLTATREEF		-	
Annexin A2-like protein	*ANXA2P2*	316–330	YYYIQQDTKGDYQKA	DR3^H^, DR52^I^	593327	
		316–329	YYYIQQDTKGDYQK		779875	
		154–169	DLEKDIISDTSGDFRK	DR3^H^, DR52^I^	16530135	
		155–169	LEKDIISDTSGDFRK		553612	
		34–53	DAERDALNIETAIKTKGVDE	DR1	591946	
		35–53	AERDALNIETAIKTKGVDE		841770	
		36–53	ERDALNIETAIKTKGVDE		233521	
		36–50	ERDALNIETAIKTKG		152509	
		37–53	RDALNIETAIKTKGVDE		648879	
		37–52	RDALNIETAIKTKGVD		648878	
		37–51	RDALNIETAIKTKGV		-	
Annexin A4	*ANXA4*	166–182	LDDALVRQDAQDLYEAG	DR1, DR3^H^, DR52^I^	1384211	
		167–182	DDALVRQDAQDLYEAG	-		
Amphiregulin	*AREG*	93–106	IPGYIVDDSVRVEQ	DR52^I^, DR52.2, DR52.3	462663	
Complement C1r subcomponent	*C1R*	305–319	KQGYQLIEGNQVLHS	DR1, DR52^I^	1243943	
Laminin subunit gamma-2	*LAMC2*	860–880	GVSDQSFQVEEAKRIKQKADS	DR1, DR52^I^	206574	
		860–879	GVSDQSFQVEEAKRIKQKAD		-	
		860–878	GVSDQSFQVEEAKRIKQKA		-	
		860–876	GVSDQSFQVEEAKRIKQ		-	
		861–876	VSDQSFQVEEAKRIKQ		-	
		862–878	SDQSFQVEEAKRIKQKA		1275060	
**Secretogranin-1** ^E^	***SCG1***	459–471	HPQGAWKELDRNY	DR	12141291	
		421–437	GRGGEPRAYFMSDTREE		1224681	
		366–385	GSEEYRAPRPQSEESWDEED		11341517	
		388–400	**NYPSLELDKMAHG** ^E^		1263477	
**Secretogranin-3** ^E^	***SCG3***	35–50	**ELSAERP** **LNEQIAEAE** ^E^	DQ2^H^	12119615	Secretory hormone
Aquaporin-1	*AQP1*	67–80	VGHISGAHLNPAVT	DQ6	-	
Neurosecretory protein	*VGF*	405–418	LFAEEEDGEAGAED	DQ2^H^	-	
Myosin-9	*MYH9*	1280–1296	VELDNVTGLLSQSDSKS	DQ6	1176523	
		1280–1295	VELDNVTGLLSQSDSK		1176522	
Cadherin-1	*CDH1*	25–41	DTRDNVYYYDEEGGGEE	DQ5.3, DQ4.4	-	
		326–340	ISVVTTGLDRESFPT	DQ3, DQ6	11344479	
Carboxypeptidase E	*CPE*	243–260	LSANLHGGDLVANYPYDE	DQ6	-	
		244–260	SANLHGGDLVANYPYDE		-	
		244–259	SANLHGGDLVANYPYD		-	
		244–258	SANLHGGDLVANYPY		-	
		245–259	ANLHGGDLVANYPYD		-	
		246–260	NLHGGDLVANYPYDE		-	
		246–259	NLHGGDLVANYPYD		-	
Secretogranin-1	*SCG1*	540–558	DNMNDNFLEGEEENELTLN	DQ	12116544	
Secretogranin-3	*SCG3*	35–50	ELSAERPLNEQIAEAE	DP	12119615 Secretory hormone	

A Position in full-length protein with signal sequence using UniProt numbering.

B For MHC-II peptides, predicted core epitopes are underlined.

C The HLA allele(s) likely to present each eluted peptide were identified from donor HLA types using NetMHCII pan 4.0 (30). For previously identified epitopes the known presenting alleleis shown in parentheses (N/A, not reported). A2 is HLA-A*02:01, A2.4 is HLA-A*02:04, A2.5 is A*02:05, A24 is HLA-A*24:01, A26 is A*26:01, A3 is A*03:01, B49 is B*49:01, B52 is B*52:01, B44.2 is B*44:02, C5 is C*05:01, C12.2 is C*12:02, C7 is C*07:01, C7.2 is C*07:02, DR1 is DRB1*01:01, DR52 is DRB3*01:01, DR52.2 is DRB3*0201 and DRB3*0202, DR52.3 is DRB3*0301, DR3 is DRB1*03:01, DR15 is DRB1*15:01, DR15.2 is DRB1*15:02, DR51 is DRB5*01:01, DR15.2 is DRB5*01:02, DR8 is DRB1*08:01, DR14.54 is DRB1*14:54, DR1.11 is DRB1*11:01, DQ6 is DQA1*01:02-DQB1*06:01, DQ6.2 is DQA1*01:02-DQB1*06:02, DQ5.3 is HLA-DQA10104-DQB10503 and HLA-DQA10401-DQB10503, DQ4.4 is HLA-DQA10104-DQB10402, HLA-DQA10401-DQB10402. Italics show alleles for which only low-resolution typing information is available.

D Immune epitope database identifier

E T cell recognition by PBMC from a T1D donor was tested and verified; see Figure 5. The validated peptide, source protein, and gene are shown in bold.

F For the peptide verified using T cell response with C1R-A2 cells expressing A*02:01, B*35:03, C*04:01:01, DPB1*04:01, DPB1*04:02.

G For the peptide verified using T cell response with QBL cells expressing A*26:01, B*18:01, Cw:05:01, DRB1*03:01, DRA1*01:01, DRB3*02:02 (DR52), DQA1*05:01 DQB1*02:01, DPA1*01, DPB1*02:02.

H HLA molecules in haplotypes most strongly associated with risk for T1D. These alleles are shown in red in Figure 2 and Supplemental Table 1.

I HLA alleles also associated with increased risk for T1D after accounting for linkage disequilibrium with the major DR-DQ risk haplotypes. These alleles are shown in blue in Figure 2 and Supplemental Table 1.

J HLA molecules in the same allele family as risk alleles (ex. A*02:01 and A*02:02) or very closely linked in the same HLA-DRB locus (ex. DRB1*03:01 and DRB3*01:01), having high sequence homology and similar MHC-peptide binding profiles to the risk alleles. These alleles are shown in orange in Figure 2 and Supplemental Table 1.

K Other alleles of interest in T1D research. These alleles are shown in magenta in Figure 2 and Supplemental Table 1

**Table:** KEY RESOURCES TABLE

REAGENT or RESOURCE	SOURCE	IDENTIFIER
Antibodies		
Anti-CD45-APC-H7 (clone 2D1)	BD Biosciences	Cat#340943; RRID:AB_40055
Anti-HLA-DR-Alexa Fluor 700 (clone L243)	Biolegend	Cat#307625; RRID:AB_493770
Anti-insulin Alex Fluor 647	Cell Signaling Technology	Cat#C27C9 RRID: unavailable
Anti-glucagon	Sigma-Aldrich	Cat#K79bB10 RRID: unavailable
Anti-HLA-DR (LB3.1)	Produced in-house	N/A
Anti-HLA-DQ (SPVL3)	Produced in-house	N/A
Anti-HLA-DP (B7/21)	Produced in-house	N/A
Anti-MHC-I (W6/32)	Produced in-house	N/A
Anti-CD3 (UCHT1)	BD Biosciences	Cat#550368; RRID: AB_393639
Anti CD28 (CD28.2)	BD Biosciences	Cat#556620; RRID: AB_396492
Anti-PD-1 (EH12.1)	BD Biosciences	Cat#562138; RRID:AB_10897007
Anti-Fas (clone SM1/23)	Enzo Life Sciences	Cat#ALX-805-008; RRID:AB_10555690
Biological samples		
Live pancreas slices	Network of Pancreatic Donors with Diabetes (nPOD)	RRID:SAMN15879420, SAMN15879467, SAMN25652261, SAMN25652262, SAMN33284295, SAMN33284296, SAMN15879467, SAMN33284295
Whole blood from HLA-typed T1D donors	UMass Chan Medical School Institutional Review Board of the University of Massachusetts	N/A
Islet equivalent cells (IEQ) cells	Prodo laboratories	RRID:SAMN22781921, SAMN16373925, SAMN16450833
Islet equivalent cells (IEQ) cells	Alberta Diabetes Institute (ADI) IsletCore	RRID:SAMN14146088, SAMN14173687, SAMN15532324
Islet equivalent cells (IEQ) cells	HPAP	RRID:SAMN19776444
Islet equivalent cells (IEQ) cells	IIDP	RRID:SAMN08783913, SAMN08783909, SAMN08773851, SAMN08769825, SAMN08769061, SAMN08768796, SAMN08768972
Spleen Tissues	Prodo laboratories and Alberta Diabetes Institute (ADI) IsletCore	N/A
Chemicals, peptides, and recombinant proteins		
AIM-V media	Gibco	Cat#12055091
DMEM	VWR	Cat#45000-324
CST^™^ OpTmizer T cell medium	Gibco	Cat#A1048501
RPMI-1640	Gibco	Cat#11875093
MEM Non-Essential Amino Acids	Gibco	Cat#11140-050
GlutaMAX^™^	Gibco	Cat#35050061
Sodium pyruvate	Gibco	Cat#11360-070
HEPES	Gibco	Cat#15630130
		
Streptomycin, Penicillin and l-glutamine	Gibco	Cat#10376-016
Antimycotic (Ambotericin B)	Gibco	Cat#15290-026
Sodium Pyruvate	Gibco	Cat#11360-070
Beta-mercaptoethanol	Gibco	Cat#21985-023
CryoStor CS10 media	Sigma Aldrich	Cat#C2874-100ML
DMSO	Gibco	Cat#21985-023
Ficoll Paque	Cytiva	Cat#17144003
NaCl	Millipore Sigma	Cat#S7653-1KG
EDTA	Millipore Sigma	Cat#EDS-500G
Human Male AB + Serum	Fisher Scientific (Grifols)	Cat#NC2620837
Human AB + Serum	GeminiBio	Cat#100-512
Recombinant Human IL-2	Proleukin Prometheus	Cat#73776-0022-01
Recombinant Human IL-7	Peprotech	Cat#200-07-10UG
Recombinant Human IL-15	Thermo Fisher Scientific	Cat#200-15-10UG
Recombinant Human IFNγ	Thermo Fisher Scientific	Cat#300-02-100UG
Recombinant Human IL-1β	R&D Systems	Cat#201-LB-010-CF
Recombinant Human TNFα	R&D Systems	Cat#10291-TA-050
Mifepristone	Thermo Fisher Scientific	Cat#H11001
Protease inhibitor cocktail	Roche	Cat#04693116001
Protease Inhibitor 2X	Thermo Fisher Scientific	Cat#A32963
Pepstatin	Millipore Sigma	Cat#10253286001
Leupeptin	Millipore Sigma	Cat#L2884-1MG
Phenylmethanesulfonyl fluoride	Millipore Sigma	Cat#P7626-1G
Phosphate Buffered Saline (PBS) Ph 7.4 (1X)	Gibco	Cat#10010023
PBS 10X	Fisher Scientific	Cat#BP3994
Tris-HCl	Fisher Scientific	Cat#BP153-500
Sodium azide	Millipore Sigma	Cat#S2002-5G
LIVE/DEAD Fixable Aqua Dead Cell Stain Kit	Thermo Fisher Scientific	Cat#L34965
Zombie Violet	Biolegend	Cat#423113
Fixation/Permeabilization Solution Kit	BD Pharmingen	Cat#554714
BD Perm/Wash	BD Biosciences	Cat#51-2091KZ
BD Cytofix/Cytoperm	BD Biosciences	Cat#51-2090KZ
PHA-M	Gibco	Cat#10576015
EasySep Human CD4 Positive Selection kit II	STEMCELL Technologies	Cat#17852
EasySep Human CD8 Positive Selection kit II	STEMCELL Technologies	Cat#17853
Zenon Alexa Fluor 488	Thermo Fisher Scientific	Cat#Z25402
Collagenase type II	Sigma	Cat#6885
Collagenase P	Fisher Scientific	Cat#11213857001
ABTS substrate solution	Roche	Cat#11-684 302 001
Octyl β-D-glucopyranoside	Millipore Sigma	Cat#O8001
NaCl 150mM pH 8.0	Millipore Sigma	Cat#S7653-1KG
CNBr activated sepharose	GE Healthcare	Cat#17-0981-01
HPLC-grade water	Thermo Fisher Scientific	Cat#TS-51140
Vydac C18 Microspin Column	The Nest Group	Cat#SEM SS18V
Acetonitrile Optima^™^ LC/MS	Fisher Scientific	Cat#A955-4
Trifluoroacetic acid	Sigma-Aldrich	Cat#T6508
Sodium chloride	Millipore Sigma	Cat#S7653-250G
Formic Acid Optima^™^ LC/MS	Fisher Scientific	Cat#A11710X1-AMP
HEPES Buffer	Fisher Scientific	Cat#BP299-100
Methanol Optima^™^ LC/MS	Fisher Scientific	Cat#A456-500
EDTA Ultrapure 0.5 M Solution, pH 8.0	Thermo Fisher Scientific	Cat#15575020
Formic Acid Optima^™^ LC/MS	Fisher Scientific	Cat#A11710X1-AMP
96 well round bottom plates	Nunc	Cat#268200
MultiScreen Immobilon-P 96 well filtration plates	EMD Millipore	Cat≠MAHAS4510
Amino-9-ethylcarbazole (AEC)/H2O2 substrate	Sigma Aldrich	Cat#132-32-1
Immobilized rProtein A 50% slurry	Repligen	Cat#IPA400HC
Peptides	21^st^ Century Biochemicals, Inc.	Custom
Peptides	AssayQuant	Custom
Recombinant HLA-DRB1*03:01protein	Produced in house	N/A
Recombinant HLA-A2*01:01	Produced in house	N/A
Critical commercial assays		
Chromium Single cell 3′ GEM,	10X Genomics	Cat#PN- 1000075
Library & Gel Bead kit v3		
Chromium Next GEM Single cell 3′ GEM, Library & Gel Bead kit v3.	10X Genomics	Cat#PN-1000121
SMARTer Stranded Total RNA-Seq Kit v.1	Pico Input Mammalian, Takara	Cat# #635007
TryPLE Express	Thermo Fisher Scientific	Cat#12604013
Human IFN-γ ELISPOT Kit	Invitrogen	Cat#88-7386-88
BD^™^ Cytometric Bead Array (CBA)	BD Biosciences	Cat#561515
Human IFN-γ Enhanced Sensitivity Flex Set		
BD^™^ Cytometric Bead Array (CBA) Human	BD Biosciences	Cat#561516
TNF Enhanced Sensitivity Flex Set		
BD^™^ Cytometric Bead Array (CBA) Human	BD Biosciences	Cat#561514
IL-10 Enhanced Sensitivity Flex Set		
BD^™^ Cytometric Bead Array (CBA) Human	BD Biosciences	Cat#561521
Enhanced Sensitivity Master Buffer Kit		
Deposited data		
Bulk and single cell RNA-Seq data	Gene Expression Omnibus	GEO:GSE255255, GSE254985
Mass Spectrometry data	MassIVE repository database	MassIVE:MSV000093843
Experimental models: Cell lines		
QBL- QBL-B-LCL	Sigma-Aldrich	Cat#94070713-1VL
A*02:01-Hmy.2B-LCL- parent cell line	ATCC	Cat#CRL-1993
K32 HLA-A* 02:01	Anadon et al.^57^	N/A
Software and algorithms		
Scaffold Q + S (version 4.6.2)	Proteome Software	https://www.proteomesoftware.com/
Scaffold PTM 3.1.0 and later versions	Proteome Software	https://www.proteomesoftware.com/
PEAKS X+ and PEAKS 11 Studio/Online	Bioinformatics Solutions	www.bioinfor.com/peaks-software/
Gibbs cluster analysis	DTU Health Tech	www.cbs.dtu.dk
Windows GraphPad Prism 7.0–9.0, 10.2.1	GraphPad Software LLC.	https://www.graphpad.com/scientific-software/prism/
NetMHCIIpan-4.0	DTU Health Tech	https://services.healthtech.dtu.dk/services/NetMHCIIpan-4.0/
NetMHCpan 4.1	DTU Health Tech	https://services.healthtech.dtu.dk/services/NetMHCpan-4.1/
Proteome Discoverer version 2.1	ThermoFisher-Scientific	https://thermo.flexnetoperations.com/control/thmo/login
Scaffold Viewer (Proteome Software, Inc)	ThermoFisher-Scientific	https://www.proteomesoftware.com/
FlowJo (version 10.3)	FLOWJO LLC	https://www.flowjo.com/
ESAT	GitHub	https://github.com/garber-lab/ESAT
ClustifyR	Bioconductor	https://doi.org/10.18129/B9.bioc.clustifyr
CellTypist	Wellcome Sanger Institute	https://www.celltypist.org
ImmunoSpot 7	Immunospot	https://immunospot.com/immunospot-software.html
DolphinNext	UMass Chan Medical School	https://github.com/UMMS-Biocore/dolphinnext
CELLxGENE	Chan Zuckerberg Cell Science Program	https://cellxgene.cziscience.com/
CTL ImmunoSpot Image Analyzer	ImmunoSpot	N/A
BD FACSDiva v.8	BD Biosciences	N/A
Other		
MilliQ water	Water Purification System, Millipore	Cat#ZRXQ010US
0.2 mm pore membrane sterile filter units	Millipore Sigma	Cat#GSWP01300
Amicon Ultra 0.5 ml centrifugal filters	Millipore Sigma	Cat#UFC501024
Ultracentrifugation tubes	Beckman Coulter	Cat#326819
Q Exactive hybrid mass spectrometer	Thermo Fisher Scientific	N/A
nano-ACQUITY UHPLC	Waters Corporation	N/A
Orbitrap Fusion^™^ Lumos^™^ Tribrid^™^	Thermo Fisher	N/A
Magic C18AQ	Bruker-Michrom	N/A
NextSeq 500/550	Illumina	N/A
BD LSRII cytometer	BD Biosciences	N/A

## Highlights

- Naturally processed MHC peptides from human pancreatic islets are recognized by T cells
- T cells targeting new epitopes were found in PBMC and infiltrating islets of T1D patients
- Inflammation, unfolded protein response, and secretory hormone processing create T1D epitopes
