# Supplementary material for: The antigen presentation landscape of cytokine-stressed human pancreatic islets
Source: Cell Rep. Author manuscript; Available in PMC 2025 Nov 18. (PMC12624573; doi:10.1016/j.celrep.2025.115927)
Supplement: 1 [file NIHMS2107237-supplement-1.pdf]

Cell Reports, Volume *44*

Supplemental information

The antigen presentation landscape of cytokine-stressed human pancreatic islets

Padma P. Nanaware, J. Mauricio Calvo-Calle, Sambra D. Redick, Mason W. Tarpley, John Cruz, Cristina C. Clement, Anthony Manganaro, Erandi E. Velarde de la Cruz, Khaja Muneeruddin, Melissa Faulkner, Jennifer P. Wang, Scott A. Shaffer, David M. Harlan, Laura Santambrogio, Sally C. Kent, and Lawrence J. Stern

Cell Reports, Volume ■ ■

Supplemental information

The antigen presentation landscape of cytokine-stressed human pancreatic islets

Padma P. Nanaware, J. Mauricio Calvo-Calle, Sambra D. Redick, Mason W. Tarpley, John Cruz, Cristina C. Clement, Anthony Manganaro, Erandi E. Velarde de la Cruz, Khaja Muneeruddin, Melissa Faulkner, Jennifer P. Wang, Scott A. Shaffer, David M. Harlan, Laura Santambrogio, Sally C. Kent, and Lawrence J. Stern

List of Supplemental Information

Supplemental Figure 1. Single-cell analysis of MHC-I and MHC-II expression before and after treatment with inflammatory cytokines

Supplemental Figure 2: Workflow for isolation of MHC-I and MHC-II immunopeptidome from cytokine-stressed human cadaveric islets

Supplemental Figure 3. Fragments from pancreatic endocrine hormone processing in MHC and control elutions.

Supplemental Figure 4. Binding affinity assays for DRB1*03:01 and HLA-A2*01:01.

Supplemental Figure 5. T cell recognition of candidate epitopes from secretory hormone eluted peptides and control peptides and pools.

Supplemental Table 1. Donor demographics.

Supplemental Table 10. Overview of newly identified T cell epitope source proteins, gene name, functions, disease associations, and function.

A

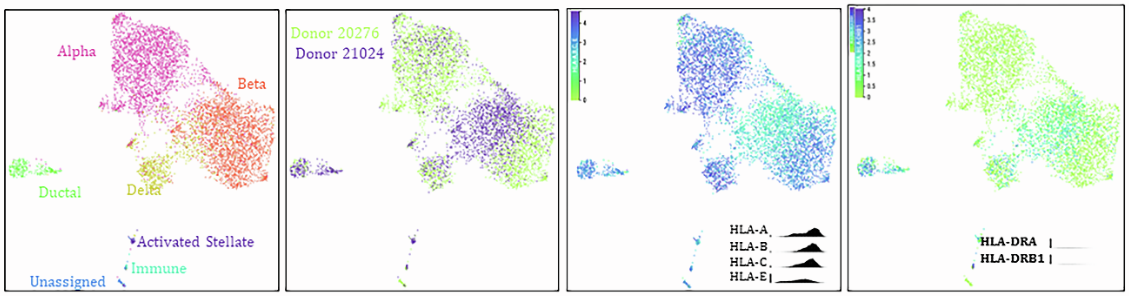

B

Islet ID	% CD45+ of total islet cells after cytokine treatment	MFI of HLA-DR on CD45+ cells with/without cytokine treatment	Fold change of HLA-DR on CD45+ cells with cytokine treatment	%glucagon + of total islet cells after cytokine treatment	MFI of HLA-DR on glucagon+ cells with/without cytokine treatment	Fold change of HLA-DR on glucagon+ cells with cytokine treatment	% insulin+ of total islet cells after cytokine treatment	MFI of HLA-DR on insulin+ cells with/without cytokine treatment	Fold change of HLA- DR on insulin+ cells with cytokine treatment
HP-20289-01	4.28	13038/1298	10.04	22.8	104.6/69.6	1.50	28.9	3210/590	5.44
HP-18081-01	6.72	2673/752	3.56	ND	ND	ND	6.70	5948/2061	2.89
HP-18076-01	2.20	735/169	4.34	ND	ND	ND	3.92	487/129	3.78
HP-19026-01	ND	ND	ND	ND	ND	ND	9.07	397/380	1.05

Supplemental Figure 1. Single-cell analysis of MHC-I and MHC-II expression before and after treatment with inflammatory cytokines A) UMAP analysis from bulk RNA sequencing for islets from two donors showing differential expression of DRA, DRB1, HLA-A, HLA-B, HLA-C and HLA-E in alpha, beta, delta, ductal, immune and activated stellate cells. B) The HLA-DR levels detected in four donors with/without cytokine treatment on CD45+, insulin positive and glucagon positive cells. ND, not done.

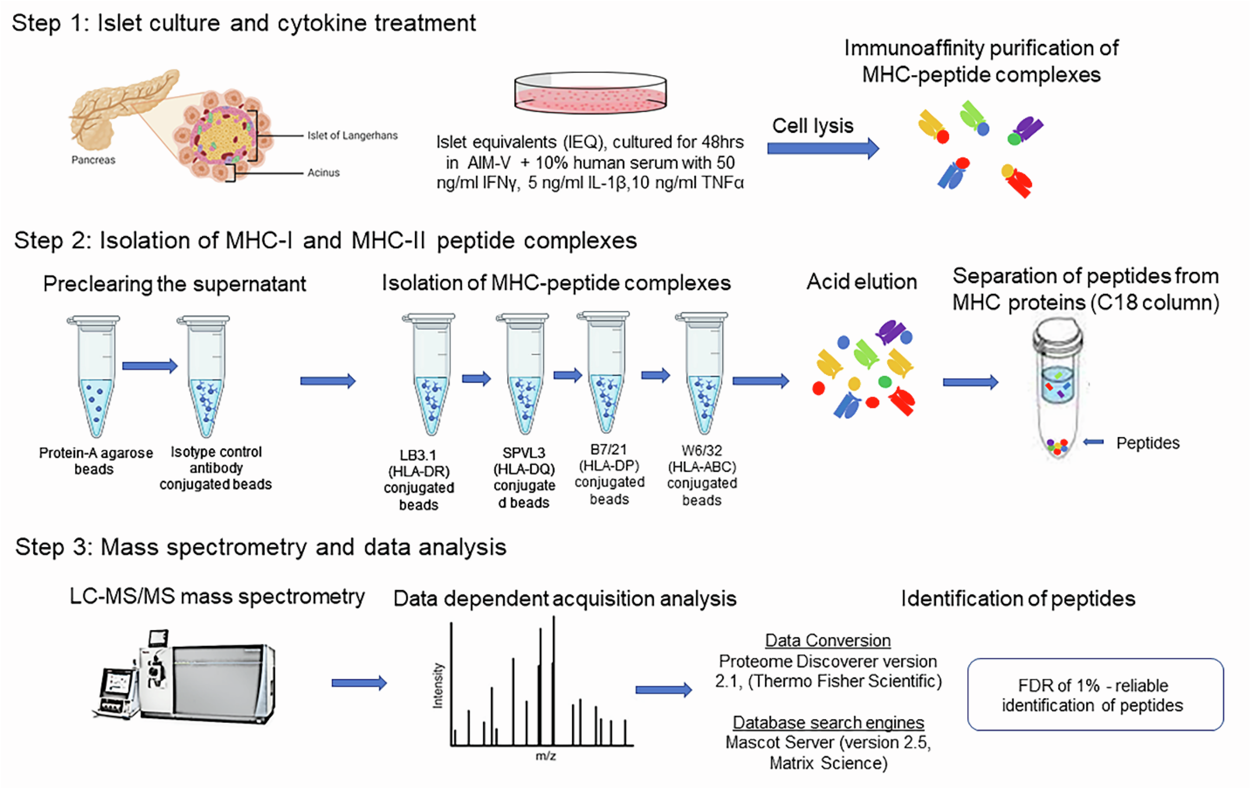

Supplemental Figure 2: Workflow for isolation of MHC-I and MHC-II immunopeptidome from cytokine-stressed human cadaveric islets. Step 1 includes islet culture and cytokine treatment for 48hrs followed by cell lysis for immunoaffinity purification. Step 2 includes isolation and MHC-I bound peptides and MHC-II (HLA-DR, DQ and DP) complexes by immunoaffinity purification followed by acid elution and separation of peptides using Vydac C4 columns. Step 3 includes identification of peptides using data dependent acquisition (DDA) method.

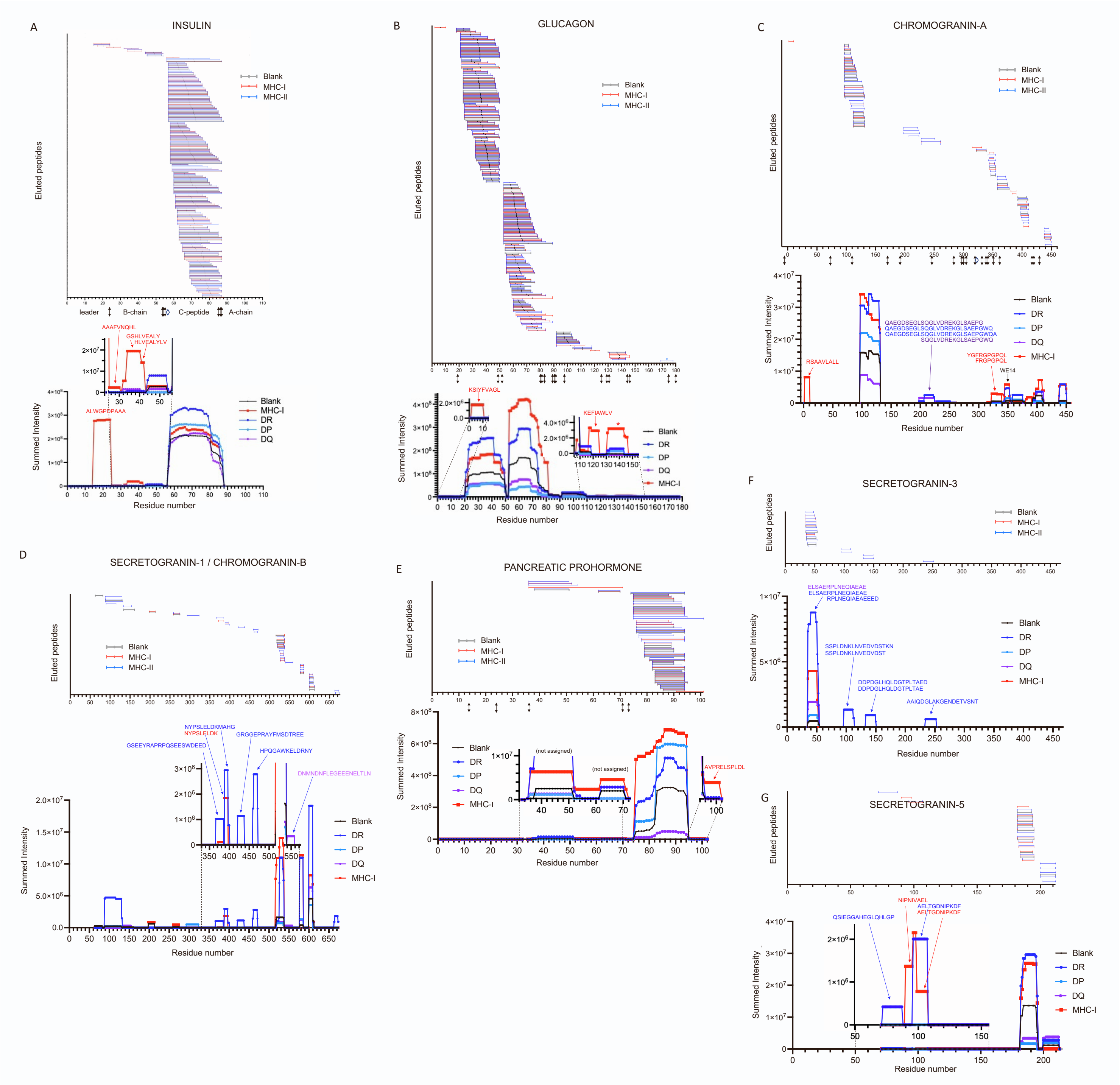

Supplemental Figure 3. Fragments from pancreatic endocrine hormone processing in MHC and control elutions. *Upper plots* show length and position of each eluted peptide along the full-length protein sequence. *Double-sided arrows* below x-axis show positions of cleavage sites for peptide hormone processing enzymes. *Diamonds* show positions of splice sites for previously characterized insulin and chromogranin hybrid peptides. *Bottom plots* show integrated intensity for all eluted peptides along the same scale. Sequences are shown for peptides considered to be specifically eluted from anti-MHC columns. Some peptides falling outside length limits are indicated with an (*). A) insulin (*INS*), B) glucagon (*GLC*), C) chromogranin-A (*CHGA*), D), secretogranin-1/chromogranin-B (*SCG1*), E), pancreatic polypeptide (*PPY*), F) secretogranin-3 (*SCG3*), G) secretogranin-5 (*SCG5*).

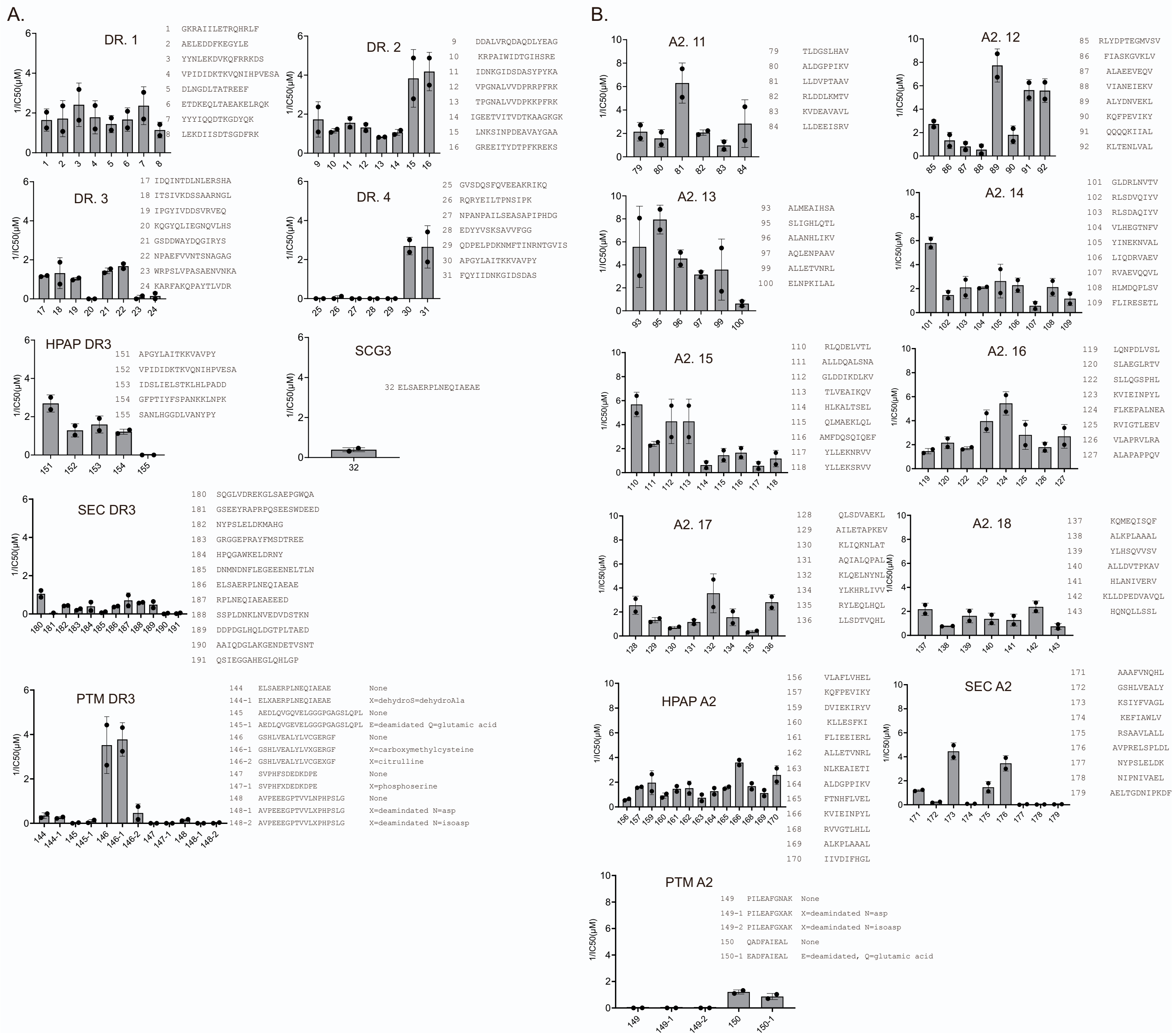

Supplemental Figure 4. Binding affinity assays for DRB1*03:01 and HLA-A2*01:01. The candidate peptides selected to test T cell responses in T cell lines derived from islets of donors and T1D/non-T1D PBMCs were characterized for their potential to bind HLA-DRB1*03:01 or HLA-A2*01:01. Binding affinities calculated for each test peptide in each pool of HLA-DRB1*03:01 (A) and HLA-A2*01:01 using fluorescence-based polarization. The 1/IC50 (μM) values are plotted for each peptide and the standard deviation is shown for two independent experiments.

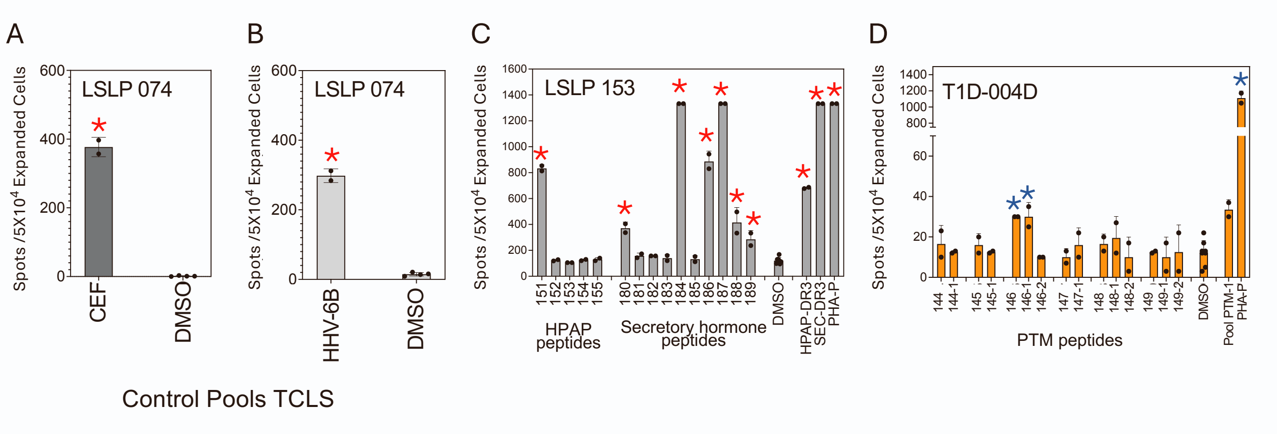

Supplemental Figure 5. T cell recognition of candidate epitopes from secretory hormone eluted peptides and control peptides and pools (A,B) T cell lines from non-diabetic control donor LSLP-074 were expanded using (A) CEF pool of previously characterized CD8 T cell epitopes from cytomegalovirus, Epstein-Barr virus, and influenza A virus, or (B) HHV-6 pool of previously characterized CD4T cell epitopes from HHV-6B virus, and then tested for reactivity with the same pool using an IFN-g ELISpot assay. Both pools contain peptides recognized by donors with a variety of HLA alleles including HLA-A*01:01 (CEF) or HLA-DRB1*03:01 (HHV6). (C) T cell line from non-diabetic control donor LSLP-153 expanded with a megapool composed of peptide pool HPAP-DR3 (Supplemental Table 9) containing candidate T1D epitopes selected using the HPAP database as source of information on genes upregulated in donors with T1D as compared to non-diabetic controls and peptide pool SEC-DR3 (Supplemental Table 9), containing candidate T1D epitopes derived from islet secretory hormones (Supplemental Figure 3). Expanded cells were tested for reactivity with the individual peptides using an IFN-g ELISpot assay. (D) T cell line from donor T1D-004D, expanded with pool PTM1 (Supplemental Table 9) containing post-translationally modified peptides and unmodified parent peptides and then tested for reactivity with the individual peptides using an IFN-g ELISpot assay. Statistically significant responses are indicated by asterisks, red for DFR2X or blue for DFR1X, as in Figure 5.

Supplementary Table 1. Donor demographics

**Sample ID**<br>**Source**<br>**RRID**	**age of demise**<br>**sex**<br>**diabetes status**	**BMI (kg/m^2)**<br>**HbA1c (%)**	**HLA[B]**<br>**Class I**	**HLA[B]**<br>**Class II**	**Experiment**
**HP-18076-01**<br>Prodo Labs<br>SAMN22781921	33<br>F<br>no diabetes	32.37<br>4.9	A2, A69<br>B35, B48<br>C4, C8	DR4, DR4<br>DR53<br>DQB1*8, DQB1*8<br>DQA1*03, DQA1*03<br>DPB1*04:01, DPB1*14:01	flow cytometry
**HP-18081-01**<br>Prodo Labs<br>A	46<br>M<br>no diabetes	33.23<br>5.4	**A2**, **A2**<br>B44, B48<br>C8, C16	**DR4**, DR15<br>DR51, **DR53**<br>DQB1*8, DQB1*6<br>DQA1*01, DQA1*03<br>DPB1*04:02, DPB1*04:02	flow cytometry<br>immunopeptidome
**HP-18101-01**<br>Prodo Labs<br>SAMN08934018	43<br>F<br>no diabetes	30.86<br>5.5	**A24**, A68<br>B61, B61<br>C10, C10	DR8, DR16<br>**DR51**<br>DQB1*7, DQB1*4<br>DQA1*04:01, DQA1*05<br>DPB1*04:02, DPB1*05:01	immunopeptidome
**HP-19026-01**<br>Prodo Labs<br>A	50<br>F<br>no diabetes	33.15<br>4.5	**A2**, A31<br>B35, B51<br>C4, C15	**DR4**, DR8<br>**DR53**<br>DQB1 4, **DQB1 8**<br>DQA1*03, DQA1*04<br>DPB1*04:02, DPB1*04:02	flow cytometry<br>islet and spleen<br>immunopeptidome
**HP-20276**<br>Prodo Labs<br>SAMN16373925	25<br>M<br>no diabetes	31.4<br>5.9	A1, A2<br>B13, B57<br>C6, C6	DR7, DR7<br>DQB1 2, DQB1 9<br>DQA1 2, DQA1 2<br>DPB1*02:01, DPB1*04:01	bulk transcriptome
**HP-20289-01**<br>Prodo Labs<br>SAMN16450833	55<br>M<br>no diabetes	30.86<br>5.6	**A*02:05**, **A*11:01**<br>B*49:01, B*52:01<br>C*07:01, C*12:02	DRB1*15:01, DRB1*15:02<br>DRB5*01:01, DRB5*01:02<br>DQB1*06:01, -<br>DQA1*01:02, DQA1*01:03<br>**DPB1*02:01**, DPB1*04:01<br>DPA1*03:01, -	flow cytometry<br>islet and spleen<br>immunopeptidome<br>transcriptome
**HP-21024-01**<br>Prodo Labs<br>A	51<br>M<br>no diabetes	20<br>5.2	**A24**, A29<br>**B7**, B44<br>C7, C16	DR7, DR15<br>DQB1 2, DQB1 6<br>DQA 1, DQA 2<br>DPB1*04:01, DPB1*04:01	transcriptome
**R360**<br>ADI IsletCore<br>SAMN14146088	51<br>F<br>no diabetes	26.1<br>4.9	**A*03:01**, A*26:01<br>**B*07:02**, B*45:01<br>C*07:02, C*06:02	DRB1*14:54, DRB1*08:01<br>**DRB3*02:02**<br>DQB1*05:03, DQB1*04:02<br>DQA1*01:04, DQA1*04:01<br>**DPB1*02:01**, DPB1*04:01<br>**DPA1*01:03**, -	islet and spleen<br>immunopeptidome
**R361**<br>ADI IsletCore<br>SAMN14173687	65<br>F<br>no diabetes	20.8<br>5.3	**A2**, A26<br>B62, B35<br>C no entry	DRB1*11, DRB1*13<br>**DR52**<br>DQB1*03:01, DQB1*06<br>DQA1* 1, DQA1* 5<br>DPB1*04:01, DPB1*10:01<br>DPA1*01:03, DPA1*02:01	islet and spleen<br>immunopeptidome
**R369**<br>ADI IsletCore<br>SAMN15532324	66<br>male<br>no diabetes	25.6<br>4.9	**A*01:01**, **A*02:01**<br>B*08:01, B*44:02<br>C*07:01, **C*05:01**	**DRB1*03:01**, DRB1*01:01<br>**DRB3*01:01**<br>**DQB1*02:01**, DQB1*05:01<br>**DQA1*05:01**, DQA1*01:01<br>**DPB1*02:01**, DPB1*04:01<br>**DPA1*01:03**	immunopeptidome
**6367**<br>nPOD<br>SAMN15879420	24<br>M<br>T1D-2 yr	25.7<br>8.8	A*02:01, A*29:02<br>B*18:01, B*44:03<br>C*07:01, C*16:01	DRB1*04:01, DRB1*07:01<br>DQA1*02:01, DQA1*03:01<br>DQB1*02:02, DQB1*03:02<br>DPB1*02:01, DPB1*11:01<br>DPA1*01:03, DPA1*06:01	transcriptome
**6414**<br>nPOD<br>SAMN15879467<br>GSE121863	23<br>M<br>T1D-0.43 yr	28.4<br>14	A*01:01, A*23:01<br>B*07:02, B*08:01<br>C*07:01, C*07:02	DRB1*03:01, DRB1*09:01<br>DQA1*05:01, DQA1*03:03<br>DQB1*02:01, DQB1*02:02<br>DPB1*04:01, DPB1*15:01<br>DPA1*01:03, DPA1*01:04	transcriptome
**6550**<br>nPOD Slice<br>SAMN25652261	25.06<br>M<br>demise at T1D onset	16.4<br>14	A*33:03, A*68:01<br>B*08:01, B*50:01<br>C*06:02, C*07:01	**DRB1* 03:01**, **DRB1*03:01**<br>DQA1*05:01, DQA1*05:01<br>DQB1*02:01, DQB1*02:01<br>DPB1*01:01, DPB1*01:01<br>DPA1*02:01, DPA1*02:01	islet-derived<br>T cell line reactivity
**6551**<br>nPOD Slice<br>SAMN25652262	20.7<br>M<br>T1D-0.58 yr	23.1<br>6.4	**A*02:01**, A*29:02<br>B*44:03, B*57:01<br>C*06:02, C*16:01	DRB1*04:01, DRB1*07:01<br>DQA1*03:03, DQA1*02:01<br>DQB1*03:01, DQB1*02:02<br>DPB1*04:01, DPB1*04:01<br>DPA1*01:03, DPA1*01:03	islet-derived<br>T cell line reactivity
**6578**<br>nPOD Slice<br>SAMN33284295	11.95<br>F<br>demise at T1D onset<br>IA2A+, ZnT8A+	22.5<br>13.6	**A*02:01**, A*03:01<br>B*18:01, B*35:01<br>C*04:01, C*07:01	DRB1*04:02, DRB1*11:01<br>DQA1*03:01, DQA1*05:05<br>DQB1*03:02, DQB1*03:01<br>DPB1*01:01, DPB1*20:01<br>DPA1*01:01; DPA1*02:01	islet-derived<br>T cell line reactivity

**6579**<br>nPOD Slice<br>SAMN33284296	13.91<br>F<br>T1D- 1 yr	18.4<br>15	A*01:01, A*01:01<br>B*08:01, B*15:17<br>C*07:01, C*07:01	**DRB1*03:01,** DRB1*13:02<br>DQA1*05:01, DQA1*01:02<br>DQB1*02:01, DQB1*06:04<br>DPB1*01:01, DPB1*02:01<br>DPA1*01:03, DPA1*02:01	islet-derived<br>T cell line reactivity
**13254**<br>IIDP<br>SAMN08783913	30<br>M<br>no diabetes	37<br>5.9	N/A	N/A	transcriptome
**13281**<br>IIDP<br>SAMN08783909	53<br>M<br>no diabetes	27.2<br>5.1	N/A	N/A	transcriptome
**14223.1**<br>Vanderbilt Univ.<br>A	20<br>M<br>T1D-7 yr	25.4<br>N/A	A2, -<br>B13, B8<br>C6, C7	DR17, DR4<br>DR52, DR53<br>DQ2, DQ8	transcriptome
**14236**<br>Prodo Labs<br>A	61<br>F<br>no diabetes	30.8<br>4.6	A1, A12<br>B51, B82<br>C10, C16	DR17, DR7<br>DR52, DR53<br>DQ2, DQ2	transcriptome
**14289**<br>Prodo Labs<br>A	39<br>F<br>no diabetes	25.8<br>5	A2, A24<br>B7, B51<br>C7, C14	DR12, DR13<br>DR52<br>DQ7, DQ7	transcriptome
**15049**<br>Prodo Labs<br>A<br>GSE121863	23<br>M<br>no diabetes	21.5<br>5.5	A2, A25<br>B7, B18<br>C7, C12	DR15, DR15<br>DQA1*01, DQA1*01<br>DP2, DP4<br>DPA1*01, DPA1*01	transcriptome
**15275**<br>IIDP<br>SAMN08773851	38<br>M<br>no diabetes	30.8<br>N/A	A3, A3<br>B7, B35<br>C4, C7	DR5, DR6<br>DQ1, DQ13	transcriptome
**15290**<br>Prodo Labs<br>A	52<br>F<br>no diabetes	39.1<br>6	A2, -<br>B18, B35<br>C4, C5	DR17, DR4<br>DR52, DR53<br>DQ2, DQ8	transcriptome
**15309**<br>Prodo Labs<br>A	41<br>F<br>no diabetes	23.6<br>5.6	N/A	N/A	transcriptome
**16054**<br>IIDP<br>SAMN08769825	63<br>M<br>no diabetes	22<br>5.8	A24, A25<br>B18, B49<br>C07, C12	DR15, DR16<br>DR51<br>DQ5, DQ6	transcriptome
**16083**<br>Prodo labs<br>A	37<br>F<br>no diabetes	25.1<br>5.5	A1, A2<br>B27, B52<br>C1, C12	DR13, DR15<br>DR51, DR52<br>DQ6, DQ6<br>DQA1*01, DQA1*01<br>DPB1*02:01, DPB1*04:01<br>DQA1*01, DQA1*01	transcriptome
**16231**<br>Prodo Labs<br>A	32<br>M<br>no diabetes	30.4<br>5.4	A1, A3<br>B7, B13<br>C6, C7	DR4,-<br>DQ7, -<br>DR53<br>DPB1*04:02, -<br>DQA1*03, -	transcriptome
**16292**<br>Prodo Labs<br>A	49<br>M<br>no diabetes	26.5<br>5.1	A1, A3<br>B65, B60<br>C10, C8	DR8, DR13<br>DQ4, DQ6<br>DR52<br>DPB1*08:01, DPB1*04:01<br>DQA1*01, DQA1*04	transcriptome
**16356**<br>IIDP<br>SAMN08769061	59<br>M<br>no diabetes	30.9<br>N/A	A1, A33<br>B15, B50<br>C6, C6	DR2, DR2<br>DQ17, DQ7	transcriptome
**17103**<br>Vanderbilt Univ.<br>A<br>GSE121863	19<br>M<br>no diabetes	N/A	N/A	N/A	transcriptome
**17180**<br>IIDP<br>SAMN08768796	44<br>F<br>no diabetes	27<br>6.1	A1, A23<br>B8, B44<br>C4, C7	DR17, DR7<br>DQ2, DQ5<br>DR52, DR53	transcriptome
**17192**<br>**(HPAP-012)**<br>HPAP<br>SAMN19776444	18<br>F<br>no diabetes	29.6<br>4.5	A*01:01, A*24:02<br>B*15:01, B*44:02<br>C*03:04, C*02:02	DRB1*11:02, DRB1*04:01<br>DRB3*02:02, DRB4*01:03<br>DQB1*03:03, DQB1*03:19<br>DQA1*03:03, DQA1*05:05<br>DPB1*04:01, DPB1*13:01<br>DPA1*01:03, -	transcriptome
**17262**<br>IIDP<br>SAMN08768972	27<br>M<br>no diabetes	30<br>5.4	A1, A2<br>B7, B50<br>C6, C7	DR103, DR7<br>DR53<br>DQ2, DQ5<br>DPB1*02:01, DPB1*13:01<br>DPA1*01, DPA1*02	transcriptome

**17346**<br>Prodo Labs<br>A	27<br>M<br>no diabetes	25.62<br>5.4	N/A	N/A	transcriptome
**18024**<br>Vanderbilt Univ.<br>A	49<br>F<br>T1D-27 yr	26.5<br>N/A	A3, A25<br>B7. B39<br>C*07, C*07	DR4, DR103<br>DR53<br>DQ7, DQ8<br>DPB1*04:02, DPB1*04:02<br>DQA1*03, DQA1*05	transcriptome
**18065**<br>IIAM/Vanderbilt Univ.<br>A	28<br>F<br>T1D-15 yr	22.2<br><br>N/A	A2, A23<br>B35, B41<br>C4, C17	DRB1*08, DRB1*09<br>DQB1*02, DQB1*06<br>DQA1*01, DQA1*03<br>DPA1*02, DPA1*03	transcriptome
**18076**<br>Prodo Labs<br>A	33<br>F<br>no diabetes	32.37<br>4.9	A2, A69<br>B35, B48<br>C4, C8	DR4, DR4<br>DR53<br>DQ8, DQ8<br>DPB1*04:01, DPB1*14:01<br>DQA1*03, DQA1*03	transcriptome
**004D**<br>UMass Chan Diabetes Center<br>A	74<br>male<br>T1D diagnosed in 1983	N/A<br>N/A	**A*02:01**, A*29:02<br>B*18:01, B*44:02	DRB1*01:01, **DRB1*03:01**	PBMC-derived T cell line reactivity
**008D**<br>UMass Chan Diabetes Center<br>A	55<br>female<br>T1D diagnosed in 1996	N/A<br>N/A	**A*02:01**, **A*02:01**<br>B*08:01, B*44:01	**DRB1*03:01**, DRB1*04:01<br>DQB1*02:01, DQB1*03:02	PBMC-derived T cell line reactivity
**042D**<br>UMass Chan Diabetes Center<br>A	45<br>male<br>T1D diagnosed in 2008	N/A<br>N/A	**A*02:01**, A*30:02<br>B*18:01, B*40:07	**DRB1*03:01**, DRB1*13:02<br>DQB1*02:01, DQB1*06:04	PBMC-derived T cell line reactivity
**339**<br>UMass Chan Diabetes Center<br>A	47<br>male<br>T1D diagnosed in 2017	N/A<br>N/A	**A*02:01**, **A*02:05**	**DRB1*03:01**, DRB1*13:02<br>DQB1*02:01, DQB1*06:09	PBMC-derived T cell line reactivity
**369**<br>UMass Chan Diabetes Center<br>A	21<br>female<br>T1D diagnosed in 2018	N/A<br>N/A	**A2**, A30	**DRB1*03**, DRB1*04<br>DQB1*02, DQB1*03	PBMC-derived T cell line reactivity
**LSLP0074**<br>New York Biologics, Inc.<br>A	N/A<br>healthy donor	N/A<br>N/A	**A*02:01**, A*33:02<br>B*44:03, B*50:01<br>C*04:01, C*06:02	**DRB1*03:01**, DRB1*15:01<br>DRB3*02:02; DRB5*01:01<br>DQB1*02:01, DQB1*06:02<br>DQA1*01:02, DQA1*05:01<br>DPB1*05:01<br>DPA1*01:03	PBMC-derived T cell line reactivity
**LSLP0091**<br>New York Biologics, Inc.<br>A	N/A<br>healthy donor	N/A<br>N/A	A*01:01, **A*02:01**<br>B*07:02, B*08:01<br>C*07:01, C*07:02	**DRB1*03:01**, DRB1*10:01<br>DRB3*01:01<br>DQB1*02:01, DQB1*05:01<br>DQA1*01:05, DQA1*05:01<br>DPB1*01:01, DPB1*04:01<br>DPA1*01:03, DPA1*02:01	PBMC-derived T cell line reactivity
**LSLP0153**<br>New York Biologics, Inc.<br>A	N/A<br>healthy donor	N/A<br>N/A	**A*03:01**, A*11:01<br>**B*07:02**, B*35:01<br>C*04:01, C*07:02	**DRB1*03:01**, DRB1*13:01<br>**DRB3*01:01 DRB3*01:01**<br>**DQB1*02:01**, DQB1*06:03<br>DQA1*01:03, **DQA1*05:01**<br>DPA1*01:03, DQA1*01:03	PBMC-derived T cell line reactivity

Supplemental Table 1. Donor demographics. The sample ID, source, RRID, age at demise, sex, disease status, BMI, HbA1C, HLA, and type of experiment sample was used are shown. HPAP donors with T1D are described in **Supplemental Table 3** and available transcriptome data from donors with T1D and with detectable beta cells was used. Databases of previously published data are shown (GSE designations). Duration of T1D is shown when available. Sample designated as nPOD Slice were provided as live, 150 -200µ slices of pancreas tissue and islets were isolated as described in methods. N/A: not available.

[A] RRID not available.

[B] Color scheme for donors for immunopeptidome and T cell reactivity experiments. **Red**: HLA molecules in haplotypes most strongly associated with risk for T1D: DR4-DQ8 (DRB1*04:01/02/04-DQA1*03:01-DQB1*03:02) and DR3-DQ2 (DRB1*03:01-DQA1*05:01-DQB1*02:01). **Blue**: HLA alleles also associated with increased risk for T1D after accounting for linkage disequilibrium with the major DR-DQ risk haplotypes. **Orange**: HLA molecules in the same allele family as risk alleles (ex. A*02:01 and A*02:02) or very closely linked in the same HLA-DRB locus (ex. DRB1*03:01 and DRB3*01:01), having high sequence homology and similar MHC-peptide binding profiles to the risk alleles, and cases where only low-resolution HLA typing was available and it was not clear whether risk allele or other member of allele family was present. **Magenta**: Other alleles of interest in T1D research A*01:01, A*03:01, A*11:01, B*07:02.

Supplemental Table 10. Overview of newly identified T cell epitope source proteins, gene name, functions, disease associations, and function.

Protein (*gene*)	Description of function	Disease associations	References
**Sensing or responding to inflammation**			
Interferon-induced protein with tetratricopeptide repeats 2<br>*IFT2*	interferon-induced<br>RNA-binding protein	anti-viral immunity<br>systemic lupus erythematosus Sjogren's syndrome<br>psoriasis<br>rheumatoid arthritis	71
Mov10 RNA Helicase<br>*MOV10*	RNA helicase<br>suppression of LINE1 retro-transposition	anti-viral immunity	72
IL-32<br>*IL32*	proinflammatory cytokine<br>induced by viral infection<br>induced by inflammatory cytokines from islets, sc-beta cells, and activated islet-infiltrating T cells<br>transcript upregulated in PBMC of children who later developed T1D<br>overlapping peptide presented by MHC-I and -II from PBMC originating B-LCL from patients with T1D<br>9 known isoforms	granulomatosis with polyangiitis<br>pancreatic cancer	73-75
Galectin-8<br>*LGALS8*	sensor of infection-induced membrane damage-inducing autophagy<br>interactions with mTOR<br>signaling via NFκB and p38 MAPK	emerging role in immune response and inflammation<br>early prognostic autoantibodies in multiple sclerosis<br>autoantibodies in antiphospholipid syndrome<br>lymphopenia in systemic lupus erythematosus	76-78
Cathepsin S<br>*CTSS*	upregulated by interferon-γ in immune and epithelial cells<br>lysosomal cysteine protease is part of the MHC-II antigen presentation pathway	dysregulated in vitiligo, RR-MS, IgA nephropathy, type 1 diabetes, other autoimmune diseases	79
TNFAIP3 Interacting Protein 1<br>*TNIP1*	A20-binding protein which plays a role in autoimmunity and tissue homeostasis through the regulation of MAPK and NFkB activation downstream of cytokine and toll-like receptors	psoriatic arthritis, rheumatoid arthritis, and systemic lupus erythematosus	80-82
Inter-alpha-trypsin inhibitor heavy chain H4<br>*ITIH4*	acute-phase response to infection, inflammation, trauma, and sepsis<br>possible biomarker for primary biliary cholangitis and primary sclerosing cholangitis	possible biomarker for primary biliary cholangitis and primary sclerosing cholangitis	83-85
LYN proto-oncogene<br>*LYN*	SRC-family protein kinase<br>cytosolic protein kinase signaling cascades induced by immune stimulation	systemic autoinflammatory disease with vasculitis (SAIDV)<br>Behçet's disease	86, 87
Dual Specificity Phosphatase 5<br>*DUSP5*	cytosolic protein kinase signaling cascades induced by immune stimulation<br>pro-survival in T cells	not reported as associated with autoimmune disease	88
**ER stress and the unfolded protein response**			
Inositol-requiring enzyme 1<br>ERN1 (a.k.a. IRE1)	primary sensor of the ER unfolded protein response<br>neoepitope formation	potential role in many autoimmune diseases	89-91

a.k.a. Endoplasmic Reticulum-To-Nucleus Signaling 1 *ERN1*			
Hexokinase 1 *HK1*	mitochondria-associated form of the enzyme catalyzing the first committed step in glucose metabolism is highly expressed in macrophages after pro-inflammatory stimulation convert between mitochondrial and cytoplasmic forms, regulating a switch between catabolic and anabolic metabolism through inhibition of GAPDH	autoantibodies associated with primary biliary cholangitis	92
**Beta cell secretory hormone processing or secretion**			
Chromogranin A *CHGA*	found in secretory vesicles of neurons and endocrine cells	target of autoreactive T cells in T1D	16, 93, 94
Islet Amyloid Polypeptide *IAPP*	hormone is released from pancreatic beta cells following food intake to regulate blood glucose<br>target of autoreactive T cells in T1D	target of autoreactive T cells in T1D	95
Proprotein Convertase Subtilisin/Kexin Type 2 a.k.a. neuroendocrine convertase 2 *PCSK2*	subtilisin-like proprotein convertase family, which includes proteases that process protein and peptide precursors trafficking through regulated or constitutive branches of the secretory pathway<br>target of autoreactive T cells in T1D	target of autoreactive T cells in T1D	16, 96
Neuroendocrine protein 7B2 a.k.a. Secretogranin V *SCG5*	secreted chaperone protein that prevents the aggregation of other secreted proteins	target of autoreactive T cells in T1D	16
Carboxypeptidase A1 *CPA1*	processing of insulin and other secretory hormones<br>pancreatic secretory protease produced primarily in acinar cells	anti-CPE antibodies associated with latent subset of adult-onset T1D	97, 98
Carboxypeptidase B1 *CPB1*	pancreatic secretory protease produced primarily in acinar cells	a highly tissue-specific protein and is a useful serum marker for acute pancreatitis and dysfunction of pancreatic transplants<br>Misfolding variants a strong, causative risk factor for chronic pancreatitis	99
Carboxypeptidase E *CPE*	M14 family of metallocarboxypeptidases<br>biosynthesis of peptide hormones and neurotransmitters, including insulin	effects in models of inflammatory bowel disease and type 2 diabetes	100-102
Secretogranin III *SCG3*	secretory granule biogenesis and hormone secretion, vascular permeabilty	model of diabetic retinopathy	103, 104
Solute Carrier Family 39 Member 14 a.k.a. ZIP14 *SLC39A14*	upregulated in beta cells by increased glucose concentrations<br>solute carrier, divalent metal transporter	related to zinc transporter ZnT8 (SLC30A8) which is an autoantibody and T cell target in T1D	105, 106
**Secretory pathways (general)**			
EH-domain containing protein 1 *EHD1*	involved in vesicle trafficking	not reported as associated with autoimmune disease	N/A
EH-domain containing protein 4 *EHD4*	involved in early endosome vesicle trafficking	not reported as associated with autoimmune disease	N/A

Syndecan binding protein<br>*SDCBP*	exosome release	not reported as associated with autoimmune disease	N/A
Cadherin-1<br>a.k.a. E-cadherin<br>*CDH1*	ubiquitous cell adhesion protein	polymorphisms in CDH1 have been associated with susceptibility to autoimmune vitiligo	107, 108
**No known disease-related or autoimmune function**			
Myosin heavy chain 9<br>*MYH9*	widely-expressed non-muscle myosin involved in cytokinesis, cell migration, and polarization	autoantibodies to MYH9 have been observed in autoimmune polyendocrine syndrome	109
Myosin Light Chain 12B<br>*MYL12B*	widely-expressed non-muscle myosin involved in cytokinesis, cell migration, and polarization<br>assembly of myosin II filaments	not reported as associated with autoimmune disease	N/A
Laminin subunit gamma 2<br>*LAMC2*	extracellular non-collagenous matrix glycoprotein<br>cell adhesion, differentiation, migration, signaling, neurite outgrowth and metastasis	autoantibodies in bullous pemphigoid	110, 111
Cordon-blue WH2 repeat protein<br>*COBL*	interacts with actin	not reported as associated with autoimmune disease	N/A
Filamin A<br>*FLNA*	crosslinks actin filaments and links actin filaments to membrane glycoproteins	targeted by T cells and antibodies in rheumatoid arthritis	112
Jagged Canonical Notch Ligand 1<br>a.k.a. CD339<br>*JAG1*	notch ligand important in development of many cell types	not reported as associated with autoimmune disease	N/A

Supplemental Table 10. Overview of newly identified T cell epitope source proteins, *gene* name, functions, disease associations, and references separated by function: sensing or responding to inflammation, ER stress and the unfolded protein response, beta cell secretory hormone processing or secretion, and secretory pathways (general). a.k.a.- also known as; N/A- not applicable. The GeneCards database was used for this overview[113].

References for Supplemental Material

16. Gonzalez-Duque, S., Azoury, M. E., Colli, M. L., Afonso, G., Turatsinze, J. V., Nigi, L., Lalanne, A. I., Sebastiani, G., Carré, A., Pinto, S., et al. (2018). Conventional and Neo-antigenic Peptides Presented by β Cells Are Targeted by Circulating Naïve CD8+ T Cells in Type 1 Diabetic and Healthy Donors. Cell Metab. *28*, 946-960.e946. https://doi.org/10.1016/j.cmet.2018.07.007
71. Wu, Y. Y., Xing, J., Li, X. F., Yang, Y. L., Shao, H. and Li, J. (2023). Roles of interferon induced protein with tetratricopeptide repeats (IFIT) family in autoimmune disease. Autoimmun Rev. *22*, 103453. https://doi.org/10.1016/j.autrev.2023.103453.
72. Nawaz, A., Shilikbay, T., Skariah, G. and Ceman, S. (2022). Unwinding the roles of RNA helicase MOV10. Wiley Interdiscip Rev RNA. *13*, e1682. https://doi.org/10.1002/wrna.1682.
73. de Albuquerque, R., Komsi, E., Starskaia, I., Ullah, U. and Lahesmaa, R. (2021). The role of Interleukin-32 in autoimmunity. Scand J Immunol. *93*, e13012. https://doi.org/10.1111/sji.13012.
74. Dettmer, R., Niwolik, I., Cirksena, K., Yoshimoto, T., Tang, Y., Mehmeti, I., Gurgul-Convey, E. and Naujok, O. (2022). Proinflammatory cytokines induce rapid, NO-independent apoptosis, expression of chemotactic mediators and interleukin-32 secretion in human pluripotent stem cell-derived beta cells. Diabetologia. *65*, 829-843. https://doi.org/10.1007/s00125-022-05654-0.
75. Nishida, A., Andoh, A., Inatomi, O. and Fujiyama, Y. (2009). Interleukin-32 expression in the pancreas. J Biol Chem. *284*, 17868-17876. https://doi.org/10.1074/jbc.M900368200.
76. Massardo, L., Metz, C., Pardo, E., Mezzano, V., Babul, M., Jarpa, E., Guzmán, A. M., André, S., Kaltner, H., Gabius, H. J., et al. (2009). Autoantibodies against galectin-8: their specificity, association with lymphopenia in systemic lupus erythematosus and detection in rheumatoid arthritis and acute inflammation. Lupus. *18*, 539-546. https://doi.org/10.1177/0961203308099973.
77. Pardo, E., Cárcamo, C., Uribe-San Martín, R., Ciampi, E., Segovia-Miranda, F., Curkovic-Peña, C., Montecino, F., Holmes, C., Tichauer, J. E., Acuña, E., et al. (2017). Galectin-8 as an immunosuppressor in experimental autoimmune encephalomyelitis and a target of human early prognostic antibodies in multiple sclerosis. PLoS One. *12*, e0177472. https://doi.org/10.1371/journal.pone.0177472.
78. Sarter, K., Janko, C., André, S., Muñoz, L. E., Schorn, C., Winkler, S., Rech, J., Kaltner, H., Lorenz, H. M., Schiller, M., et al. (2013). Autoantibodies against galectins are associated with antiphospholipid syndrome in patients with systemic lupus erythematosus. Glycobiology. *23*, 12-22. https://doi.org/10.1093/glycob/cws120.
79. Smyth, P., Sasiwachirangkul, J., Williams, R. and Scott, C. J. (2022). Cathepsin S (CTSS) activity in health and disease - A treasure trove of untapped clinical potential. Mol Aspects Med. *88*, 101106. https://doi.org/10.1016/j.mam.2022.101106.
80. Allanore, Y., Saad, M., Dieudé, P., Avouac, J., Distler, J. H., Amouyel, P., Matucci-Cerinic, M., Riemekasten, G., Airo, P., Melchers, I., et al. (2011). Genome-wide scan identifies TNIP1, PSORS1C1, and RHOB as novel risk loci for systemic sclerosis. PLoS Genet. *7*, e1002091. https://doi.org/10.1371/journal.pgen.1002091.
81. Bowes, J., Orozco, G., Flynn, E., Ho, P., Brier, R., Marzo-Ortega, H., Coates, L., McManus, R., Ryan, A. W., Kane, D., et al. (2011). Confirmation of TNIP1 and IL23A as susceptibility loci for psoriatic arthritis. Ann Rheum Dis. *70*, 1641-1644. https://doi.org/10.1136/ard.2011.150102.
82. Shamilov, R. and Aneskievich, B. J. (2018). TNIP1 in Autoimmune Diseases: Regulation of Toll-like Receptor Signaling. J Immunol Res. *2018*, 3491269. https://doi.org/10.1155/2018/3491269.
83. Laursen, T. L., Bossen, L., Pihl, R., Troldborg, A., Sandahl, T. D., Hansen, A. G., Folserass, T., Vesterhus, M., Grønbæk, H. and Thiel, S. (2022). Highly Increased Levels of Inter-α-inhibitor Heavy Chain 4 (ITIH4) in Autoimmune Cholestatic Liver Diseases. J Clin Transl Hepatol. *10*, 796-802. https://doi.org/10.14218/jcth.2021.00515.
84. Piñeiro, M., Andrés, M., Iturralde, M., Carmona, S., Hirvonen, J., Pyörälä, S., Heegaard, P. M., Tjørnehøj, K., Lampreave, F., Piñeiro, A., et al. (2004). ITIH4 (inter-alpha-trypsin inhibitor heavy chain 4) is a new acute-phase protein isolated from cattle during experimental infection. Infect Immun. *72*, 3777-3782. https://doi.org/10.1128/iai.72.7.3777-3782.2004.
85. Zhuo, L., Hascall, V. C. and Kimata, K. (2004). Inter-alpha-trypsin inhibitor, a covalent protein-glycosaminoglycan-protein complex. J Biol Chem. *279*, 38079-38082. https://doi.org/10.1074/jbc.R300039200.
86. de Jesus, A. A., Chen, G., Yang, D., Brdicka, T., Ruth, N. M., Bennin, D., Cebecauerova, D., Malcova, H., Freeman, H., Martin, N., et al. (2023). Constitutively active Lyn kinase causes a cutaneous small vessel vasculitis and liver fibrosis syndrome. Nat Commun. *14*, 1502. https://doi.org/10.1038/s41467-023-36941-y.
87. Papadopoulou, C., Omoyinmi, E., Standing, A., Pain, C. E., Booth, C., D'Arco, F., Gilmour, K., Buckland, M., Eleftheriou, D. and Brogan, P. A. (2019). Monogenic mimics of Behçet's disease in the young. Rheumatology (Oxford). *58*, 1227-1238. https://doi.org/10.1093/rheumatology/key445.
88. Kutty, R. G., Xin, G., Schauder, D. M., Cossette, S. M., Bordas, M., Cui, W. and Ramchandran, R. (2016). Dual Specificity Phosphatase 5 Is Essential for T Cell Survival. PLoS One. *11*, e0167246. https://doi.org/10.1371/journal.pone.0167246.

89. Bettigole, S. E. and Glimcher, L. H. (2015). Endoplasmic reticulum stress in immunity. Annu Rev Immunol. *33*, 107-138. https://doi.org/10.1146/annurev-immunol-032414-112116.
90. Eizirik, D. L., Pasquali, L. and Cnop, M. (2020). Pancreatic β-cells in type 1 and type 2 diabetes mellitus: different pathways to failure. Nat Rev Endocrinol. *16*, 349-362. https://doi.org/10.1038/s41574-020-0355-7.
91. Junjappa, R. P., Patil, P., Bhattarai, K. R., Kim, H. R. and Chae, H. J. (2018). IRE1α Implications in Endoplasmic Reticulum Stress-Mediated Development and Pathogenesis of Autoimmune Diseases. Front Immunol. *9*, 1289. https://doi.org/10.3389/fimmu.2018.01289.
92. De Jesus, A., Keyhani-Nejad, F., Pusec, C. M., Goodman, L., Geier, J. A., Stoolman, J. S., Stanczyk, P. J., Nguyen, T., Xu, K., Suresh, K. V., et al. (2022). Hexokinase 1 cellular localization regulates the metabolic fate of glucose. Mol Cell. *82*, 1261-1277.e1269. https://doi.org/10.1016/j.molcel.2022.02.028.
93. Stadinski, B. D., Delong, T., Reisdorph, N., Reisdorph, R., Powell, R. L., Armstrong, M., Piganelli, J. D., Barbour, G., Bradley, B., Crawford, F., et al. (2010). Chromogranin A is an autoantigen in type 1 diabetes. Nat Immunol. *11*, 225-231. https://doi.org/10.1038/ni.1844.
94. Gottlieb, P. A., Delong, T., Baker, R. L., Fitzgerald-Miller, L., Wagner, R., Cook, G., Rewers, M. R., Michels, A. and Haskins, K. (2014). Chromogranin A is a T cell antigen in human type 1 diabetes. J Autoimmun. *50*, 38-41. https://doi.org/10.1016/j.jaut.2013.10.003.
95. Denroche, H. C. and Verchere, C. B. (2018). IAPP and type 1 diabetes: implications for immunity, metabolism and islet transplants. J Mol Endocrinol. *60*, R57-R75. https://doi.org/10.1530/jme-17-0138.
96. Päth, G., Perakakis, N., Mantzoros, C. S. and Seufert, J. (2022). PCSK9 inhibition and cholesterol homeostasis in insulin producing β-cells. Lipids Health Dis. *21*, 138. https://doi.org/10.1186/s12944-022-01751-6.
97. Uhlig, R., Contreras, H., Weidemann, S., Gorbokon, N., Menz, A., Büscheck, F., Luebke, A. M., Kluth, M., Hube-Magg, C., Hinsch, A., et al. (2022). Carboxypeptidase A1 (CPA1) Immunohistochemistry Is Highly Sensitive and Specific for Acinar Cell Carcinoma (ACC) of the Pancreas. Am J Surg Pathol. *46*, 97-104. https://doi.org/10.1097/pas.0000000000001817.
98. Yang, L., Zhou, Z. G., Tan, S. Z., Huang, G., Jin, P., Yan, X., Li, X., Peng, H. and Hagopian, W. (2008). Carboxypeptidase-H autoantibodies differentiate a more latent subset of autoimmune diabetes from phenotypic type 2 diabetes among Chinese adults. Ann N Y Acad Sci. *1150*, 263-266. https://doi.org/10.1196/annals.1447.037.
99. Sahin-Tóth, M. (2017). Genetic risk in chronic pancreatitis: the misfolding-dependent pathway. Curr Opin Gastroenterol. *33*, 390-395. https://doi.org/10.1097/mog.0000000000000380.
100. Bär, F., Föh, B., Pagel, R., Schröder, T., Schlichting, H., Hirose, M., Lemcke, S., Klinger, A., König, P., Karsten, C. M., et al. (2014). Carboxypeptidase E modulates intestinal immune homeostasis and protects against experimental colitis in mice. PLoS One. *9*, e102347. https://doi.org/10.1371/journal.pone.0102347.
101. Chen, Y. C., Taylor, A. J., Fulcher, J. M., Swensen, A. C., Dai, X. Q., Komba, M., Wrightson, K. L. C., Fok, K., Patterson, A. E., Klein Geltink, R. I., et al. (2023). Deletion of Carboxypeptidase E in β-Cells Disrupts Proinsulin Processing but Does Not Lead to Spontaneous Development of Diabetes in Mice. Diabetes. *72*, 1277-1288. https://doi.org/10.2337/db22-0945.
102. Ji, L., Wu, H. T., Qin, X. Y. and Lan, R. (2017). Dissecting carboxypeptidase E: properties, functions and pathophysiological roles in disease. Endocr Connect. *6*, R18-R38. https://doi.org/10.1530/ec-17-0020.
103. Herold, Z., Doleschall, M. and Somogyi, A. (2021). Role and function of granin proteins in diabetes mellitus. World J Diabetes. *12*, 1081-1092. https://doi.org/10.4239/wjd.v12.i7.1081.
104. LeBlanc, M. E., Wang, W., Chen, X., Caberoy, N. B., Guo, F., Shen, C., Ji, Y., Tian, H., Wang, H., Chen, R., et al. (2017). Secretogranin III as a disease-associated ligand for antiangiogenic therapy of diabetic retinopathy. J Exp Med. *214*, 1029-1047. https://doi.org/10.1084/jem.20161802.
105. Maxel, T., Smidt, K., Petersen, C. C., Honoré, B., Christensen, A. K., Jeppesen, P. B., Brock, B., Rungby, J., Palmfeldt, J. and Larsen, A. (2019). The zinc transporter Zip14 (SLC39a14) affects Beta-cell Function: Proteomics, Gene expression, and Insulin secretion studies in INS-1E cells. Sci Rep. *9*, 8589. https://doi.org/10.1038/s41598-019-44954-1.
106. Søgaard, K. L., Ellervik, C., Svensson, J. and Thorsen, S. U. (2017). The Role of Iron in Type 1 Diabetes Etiology: A Systematic Review of New Evidence on a Long-Standing Mystery. Rev Diabet Stud. *14*, 269-278. https://doi.org/10.1900/rds.2017.14.269.
107. Almasi-Nasrabadi, M., Amoli, M. M., Robati, R. M., Rajabi, F., Ghalamkarpour, F. and Gauthier, Y. (2019). CDH1 and DDR1 common variants confer risk to vitiligo and autoimmune comorbidities. Gene. *700*, 17-22. https://doi.org/10.1016/j.gene.2019.03.026.
108. Tarlé, R. G., Silva de Castro, C. C., do Nascimento, L. M. and Mira, M. T. (2015). Polymorphism of the E-cadherin gene CDH1 is associated with susceptibility to vitiligo. Exp Dermatol. *24*, 300-302. https://doi.org/10.1111/exd.12641.
109. Lindh, E., Brännström, J., Jones, P., Wermeling, F., Hässler, S., Betterle, C., Garty, B. Z., Stridsberg, M., Herrmann, B., Karlsson, M. C., et al. (2013). Autoimmunity and cystatin SA1 deficiency behind chronic mucocutaneous candidiasis in autoimmune polyendocrine syndrome type 1. J Autoimmun. *42*, 1-6. https://doi.org/10.1016/j.jaut.2012.10.001.

110. Izumi, R., Fujimoto, M., Yazawa, N., Nakashima, H., Asashima, N., Watanabe, R., Kuwano, Y., Kurokawa, M., Hashimoto, T. and Tamaki, K. (2007). Bullous pemphigoid positive for anti-BP180 and anti-laminin 5 antibodies in a patient with graft-vs-host disease. J Am Acad Dermatol. *56*, S94-97. https://doi.org/10.1016/j.jaad.2006.10.986.

111. Bekou, V., Thoma-Uszynski, S., Wendler, O., Uter, W., Schwietzke, S., Hunziker, T., Zouboulis, C. C., Schuler, G., Sorokin, L. and Hertl, M. (2005). Detection of laminin 5-specific auto-antibodies in mucous membrane and bullous pemphigoid sera by ELISA. J Invest Dermatol. *124*, 732-740. https://doi.org/10.1111/j.0022-202X.2005.23646.x.

112. Pianta, A., Arvikar, S. L., Strle, K., Drouin, E. E., Wang, Q., Costello, C. E. and Steere, A. C. (2017). Two rheumatoid arthritis-specific autoantigens correlate microbial immunity with autoimmune responses in joints. J Clin Invest. *127*, 2946-2956. https://doi.org/10.1172/jci93450.

113. Stelzer, G., Rosen, N., Plaschkes, I., Zimmerman, S., Twik, M., Fishilevich, S., Stein, T. I., Nudel, R., Lieder, I., Mazor, Y., et al. (2016). The GeneCards Suite: From Gene Data Mining to Disease Genome Sequence Analyses. Curr Protoc Bioinformatics. *54*, 1.30.31-31.30.33. https://doi.org/10.1002/cpbi.5.
